# A “One-Stone-Three-Birds” Inspired Nanoplatform for Multitargeted Ulcerative Colitis Therapy via Combined Aryl Hydrocarbon Receptor Activation and Reactive Oxygen Species Scavenging

**DOI:** 10.34133/bmr.0356

**Published:** 2026-05-12

**Authors:** Kai Dong, Zelin Guan, Danyang Wang, Jinyao Sun, Ying Zhang, Cuiyu You, Yuanming Xing

**Affiliations:** ^1^ Department of Pharmacy, The First Affiliated Hospital of Xi’an Jiaotong University, Xi’an, Shaanxi, China.; ^2^ School of Pharmacy, Xi’an Jiaotong University, Xi’an, Shaanxi, China.; ^3^ Department of Cardiovascular Medicine, The First Affiliated Hospital of Xi’an Jiaotong University, Xi’an, Shaanxi, China.

## Abstract

In the pathogenesis of ulcerative colitis, inflammatory responses, mucosal injury, and gut dysbiosis form a mutually reinforcing network that perpetuates disease progression. Current clinical interventions can rarely modulate these pathological modules simultaneously, underscoring the urgent need for more effective therapeutic strategies. Here, we report a dual-functional nanotherapy that couples aryl hydrocarbon receptor (AhR) activation with on-site reactive oxygen species (ROS) scavenging. An amphiphilic copolymer, HA-TK-LA (HTL), was synthesized by grafting lipoic acid (LA) to hyaluronic acid (HA) via a ROS-cleavable thioketal (TK) linker. The copolymer self-assembles into ~130-nm nanoparticles that encapsulate the natural AhR ligand indole-3-acetic acid (IAA) with a high loading efficiency of 73%. The resulting IAA@HTL nanoparticles exhibit ROS/glutathione-triggered drug release and are actively taken up by inflamed intestinal epithelial cells and M1 macrophages through CD44-mediated endocytosis, leading to suppressed epithelial apoptosis and macrophage repolarization toward an anti-inflammatory phenotype. In dextran sulfate sodium-induced murine colitis, IAA@HTL specifically accumulates in the inflamed colon and markedly alleviates disease activity. Mechanistically, IAA and LA released from the nanoparticles synergistically activate aryl hydrocarbon receptor/cytochrome P450 family 1 subfamily A member 1 and nuclear factor erythroid 2-related factor 2/heme oxygenase-1 signaling, skew macrophages toward an anti-inflammatory state, dampen proinflammatory cytokine release, and reinforce the mucosal barrier by up-regulating tight-junction proteins and interleukin-22 secretion. As inflammation subsides and mucosal integrity is restored, the gut microbiota gradually returns to homeostasis. Collectively, this study establishes a ROS-scavenging and AhR-activating nanodelivery system that achieves a “one-stone-three-birds” outcome—attenuating inflammation, repairing mucosa, and rebalancing microbiota—providing new experimental evidence and theoretical support for ulcerative colitis therapy.

## Introduction

Ulcerative colitis (UC) is a chronic, nonspecific inflammatory disorder confined to the rectal and colonic mucosa, clinically manifesting as diarrhea, abdominal pain, and bloody stools [1]. The pathogenesis of UC is multifactorial, wherein genetically susceptible hosts exposed to environmental stressors exhibit compromised epithelial-barrier function, facilitating bacterial translocation and sustained immune activation [2]. During this process, dysbiosis, mucosal injury, and inflammatory responses mutually reinforce one another, forming a dynamic interactive network that chronically drives disease progression [3]. Current clinical therapy relies mainly on anti-inflammatory and immunosuppressive agents, which are often ineffective in restoring gut microbial homeostasis or mucosal integrity and are often accompanied by substantial long-term adverse effects [4]. Therefore, novel therapeutic strategies for UC capable of simultaneously resolving inflammation, repairing the mucosa, and reestablishing microbial homeostasis are urgently needed.

The aryl hydrocarbon receptor (AhR) is a widely expressed ligand-activated transcription factor primarily localized in the cytoplasm [5]. Among its key endogenous ligands are indole derivatives, such as indole-3-acetic acid (IAA) and indole-3-propionic acid, which are produced through gut microbial metabolism of tryptophan [6]. Ligand binding triggers AhR nuclear translocation and the initiation of target gene transcription. This signaling pathway converges with multiple regulatory networks governing inflammation, immune homeostasis, and cellular differentiation, underscoring its essential role in sustaining intestinal immune equilibrium [7]. From an immunological perspective, AhR activation promotes the proliferation and functional specification of innate lymphoid cells, fine-tunes the balance of T-helper cell subsets, and influences macrophage polarization, thereby exerting precise control over the release of inflammatory mediators [8]. In the context of mucosal homeostasis, AhR helps maintain epithelial integrity by suppressing proapoptotic and necroptotic pathways, augments mucin and antimicrobial peptide synthesis through interleukin-22 (IL-22)-dependent mechanisms, and reinforces the intestinal barrier via up-regulation of tight junction (TJ) proteins [9]. Therefore, targeted AhR activation represents a promising therapeutic strategy for immunomodulation and mucosal repair.

However, AhR expression and activity are subject to modulation by inflammatory signaling pathways and related stimuli. For example, Wang et al. [10] reported that lipopolysaccharide (LPS) induces microglial activation and central nervous system inflammation, elevates proinflammatory cytokines such as IL-1β and IL-6, and markedly down-regulates AhR protein levels. Similarly, Vogel et al. [11] showed that LPS-induced nuclear factor κB (NF-κB) activation exerts negative feedback on AhR expression, thereby attenuating its regulatory capacity. These findings suggest that an inflammatory microenvironment can disrupt AhR signaling, posing a substantial challenge to its effective activation under pathological conditions. In the progression of UC, excessive reactive oxygen species (ROS) function as critical drivers of disease pathogenesis [12]. ROS are generated largely via mitochondrial dysfunction and nicotinamide adenine dinucleotide phosphate oxidase activation triggered by inflammatory cytokines. Beyond activating major proinflammatory pathways such as NF-κB and mitogen-activated protein kinase, ROS further amplify the inflammatory cascade by promoting additional cytokine release, thereby sustaining disease progression [13]. Therefore, the clearance of excess ROS in the inflammatory microenvironment may establish a favorable setting for AhR activation and the full realization of its regulatory functions.

As a principal indole derivative, IAA has been repeatedly shown to exert immunomodulatory and mucosal-repairing effects via AhR activation. In a study by Yang et al. [14], fructo-oligosaccharides were used to remodel gut microbiota-dependent tryptophan metabolism, markedly elevating colonic IAA levels in mice. The subsequent AhR activation enhanced IL-22 production and tight-junction protein expression, ultimately alleviating dextran sulfate sodium (DSS)-induced colitis. Similarly, Wang et al. demonstrated that the traditional herbal formula Wuji Wan promotes *Lactobacillus*-mediated tryptophan catabolism and IAA generation, leading to AhR pathway activation, reinforcement of intestinal barrier function, and suppression of inflammatory responses, thereby mitigating DSS-induced colitis [15]. Furthermore, IAA–AhR signaling stimulates IL-22 secretion, induces antimicrobial peptide expression in epithelial cells, and acts synergistically with mucins to restrict pathogen colonization and promote microbial homeostasis [16]. Therefore, targeted delivery of IAA to inflamed intestinal mucosa represents a promising strategy to concurrently support epithelial repair, resolve inflammation, and reestablish microbiota balance. Lipoic acid (LA), a natural dithiolane-containing antioxidant, exhibits high electron density and electrophilicity, enabling efficient scavenging of a broad spectrum of ROS, including hydroxyl radicals (·OH), hydrogen peroxide (H_2_O_2_), and singlet oxygen (^1^O_2_) [17]. Together, these findings support the concept that localized codelivery of IAA and LA to inflamed colonic tissue could leverage LA’s potent ROS scavenging capacity to mitigate oxidative stress and inflammatory burden, thereby creating a favorable microenvironment for IAA-mediated AhR activation. This combination strategy holds promise for synergistically suppressing inflammation, accelerating mucosal healing, and correcting microbial dysbiosis, offering an integrated therapeutic approach for UC.

However, both IAA and LA are hydrophobic molecules with poor oral bioavailability, making them difficult to achieve the desired therapeutic effect. The disulfide bond in LA can be responsively cleaved under the high intracellular concentration of glutathione (GSH) in inflammatory conditions, transforming LA from a hydrophobic to a hydrophilic state [18]. Based on this property, an amphiphilic polymer carrier can be constructed using LA as the hydrophobic segment for coloading IAA, enabling not only dual-drug codelivery but also GSH-responsive drug release [19]. The lamina propria of the intestinal mucosa is a vital component of the gut lining, rich in immune cells such as macrophages, dendritic cells, and lymphocytes, forming the frontline of intestinal immune surveillance and response. It also possesses a dense capillary network responsible for delivering oxygen and nutrients to immune and epithelial cells [20]. Under inflammatory conditions, proinflammatory cytokines act on vascular endothelial cells, increasing intercellular gaps and creating endothelial gaps that enable passive extravasation of long-circulating nanoparticles—a process known as the enhanced permeability and retention (EPR) effect [21]. Additionally, inflammation degrades the extracellular matrix, increasing the permeability of the basement membrane between the epithelium and lamina propria, thereby enhancing the likelihood of nanoparticle uptake by epithelial cells.

**Fig. 1.**
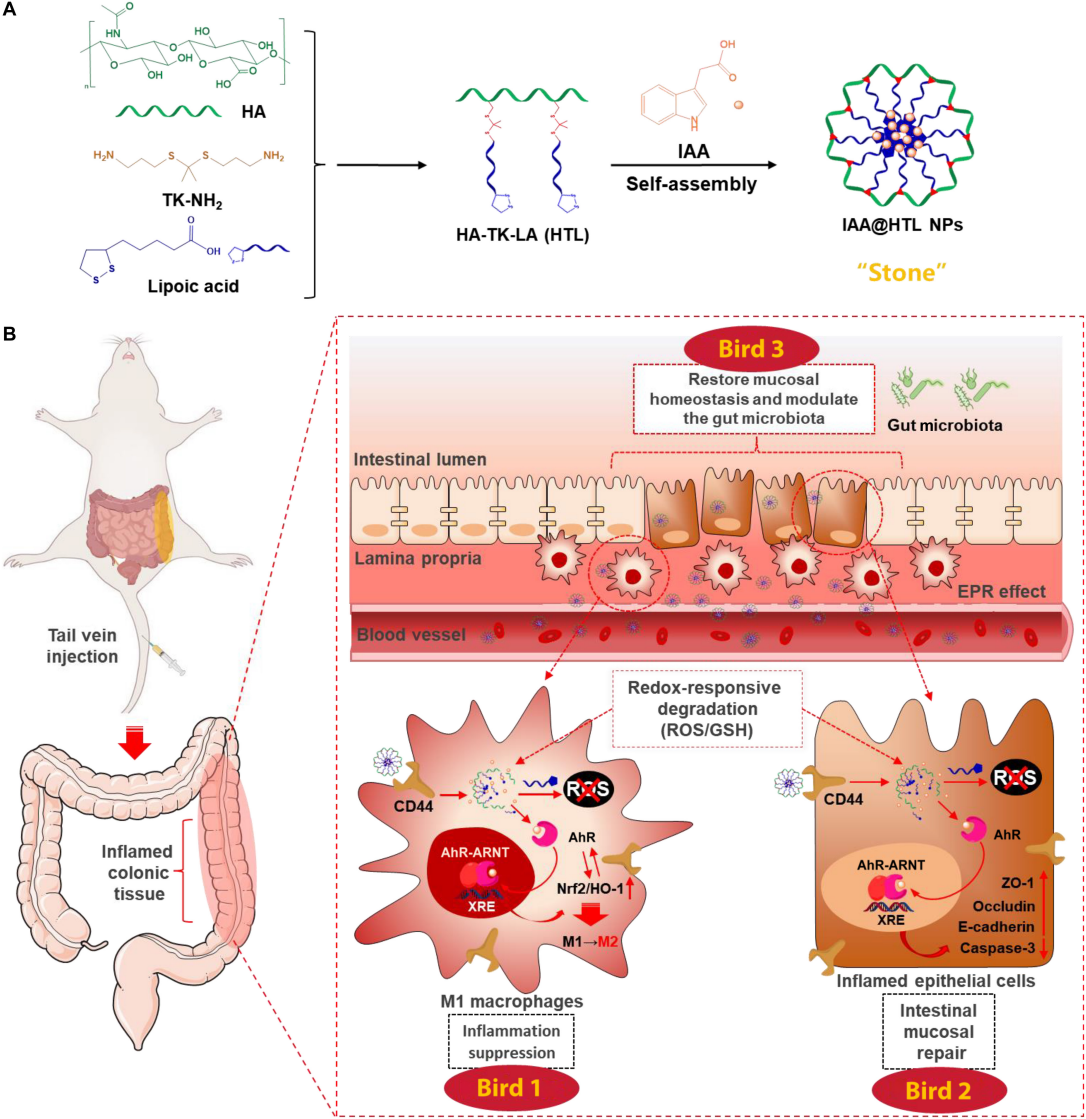
(A) Schematic illustration of the synthesis of the graft copolymer hyaluronic acid-thioketal-lipoic acid (HA-TK-LA [HTL]) and the preparation of IAA@HTL nanoparticles. (B) Following intravenous injection, the nanoparticles target inflamed colon tissue via enhanced permeability and retention (EPR) and CD44-mediated uptake. Intracellular degradation releases indole-3-acetic acid (IAA) and lipoic acid (LA), which act synergistically to exert a “one-stone-three-birds” effect: polarizing macrophages to an anti-inflammatory phenotype, enhancing epithelial-barrier function, and ultimately restoring gut microbiota homeostasis. AhR, aryl hydrocarbon receptor. ARNT, aryl hydrocarbon receptor nuclear translocator; XRE, xenobiotic response element.

Therefore, intravenous administration of IAA-loaded LA-based nanoparticles can exploit the inflamed lamina propria to simultaneously enhance their uptake by both immune and epithelial cells. Because the CD44 receptor is markedly up-regulated on inflamed epithelial- and immune-cell membranes, we selected the glycosaminoglycan hyaluronic acid (HA)—which exhibits high affinity for CD44—as the hydrophilic block and connected it to LA via a ROS-cleavable thioketal (TK) linker to create an amphiphilic graft copolymer, HA-TK-LA (HTL) [22,23]. Self-assembly of HTL in the presence of IAA yielded IAA@HTL nanoparticles. After intravenous injection, these nanoparticles accumulate in the inflamed colonic lamina propria through the EPR effect and are then actively internalized by epithelial cells and M1 macrophages via HA–CD44 interactions. Intracellular ROS and GSH rapidly cleave the TK and disulfide bonds, respectively, inducing carrier disassembly and synchronized release of IAA and LA: LA scavenges excess ROS, while IAA binds AhR and activates its downstream signaling. This synergistic action suppresses epithelial cell death, up-regulates barrier-associated proteins, and accelerates mucosal repair; simultaneously, nuclear factor erythroid 2-related factor 2/heme oxygenase-1 (Nrf2/HO-1) pathway activation drives macrophage polarization toward an anti-inflammatory phenotype and curtails proinflammatory cytokine production. Moreover, the system markedly reshapes the gut microbiota, reducing pathogens and enriching beneficial taxa (Fig. 1). Thus, IAA@HTL achieves a “one-stone-three-birds” outcome—quenching inflammation, restoring the mucosal barrier, and reestablishing microbial homeostasis—providing new experimental evidence and a theoretical basis for UC therapy.

## Materials and Methods

### Materials

IAA, LA, HA (Lot No.: H58745LCT1, molecular weight = 100 kDa), 2,2’-(propane-2,2-diylbis(sulfanediyl))diethanamine (TK-NH_2_),1-ethyl-(3-dimethylaminopropyl)carbodiimide hydrochloride (EDC·HCl), N-hydroxy succinimide (NHS), and oleic acid (OA) were purchased from Tianjin Heowns Biochemical Technology Co., Ltd. (Tianjin, China). 3,3’,5,5’-Tetramethylbenzidine (TMB), crystal violet, riboflavin, rhodamine 123 (R123), and LPS were obtained from Shanghai Aladdin Biochemical Technology Co., Ltd. (Shanghai, China). The MTT cell proliferation assay kit, calcein-acetoxymethyl ester (AM)/propidium iodide (PI) live/dead-cell double-staining kit, Annexin V-fluorescein isothiocyanate (FITC)/PI apoptosis detection kit, 2',7'-dichlorodihydrofluorescein diacetate (DCFH-DA) ROS detection probe, and enzyme-linked immunosorbent assay (ELISA) kits for tumor necrosis factor-α (TNF-α), IL-1β, IL-10, transforming growth factor-β1 (TGF-β1), and IL-22 were purchased from Beijing Solarbio Science & Technology Co., Ltd. (Beijing, China). phycoerythrin-conjugated rat anti-mouse CD86 antibody, allophycocyanin-conjugated rat anti-mouse CD206 antibody, as well as zonula occludens-1, Occludin, and E-cadherin antibodies, were acquired from Xi’an XSXCbio Biotechnology Co., Ltd. (Xi’an, China). All other reagents were of analytical grade and purchased from standard commercial sources.

The HT-29 human colon cancer cell line and RAW264.7 mouse monocyte macrophage leukemia cell line were purchased from the Cell Bank of the Shanghai Institute of Biochemistry and Cell Biology, Chinese Academy of Sciences (Shanghai, China). All cells were cultured in Dulbecco’s modified Eagle’s medium high-glucose medium (Corning) supplemented with 10% fetal bovine serum (FBS, Gibco), 100 IU/ml penicillin, and 100 μg/ml streptomycin (HyClone), and maintained in a 37 °C, 5% CO_2_ incubator (Thermo Scientific) under constant humidity. Cells in the logarithmic growth phase were used for experiments to ensure data consistency. Six-week-old male Institute of Cancer Research mice (weighing 20 to 25 g) were provided by the Experimental Animal Center of Xi’an Jiaotong University. All mice were housed in an SPF-grade barrier environment with room temperature maintained at 20 to 25 °C, relative humidity at 50% to 60%, and free access to food and water. Before experiments, the animals underwent a 1-week acclimatization period to minimize stress effects. All animal experimental procedures strictly adhered to the protocol approved by the Laboratory Animal Ethics Committee of Xi’an Jiaotong University (Approval No.: XJTULAC2019-068) and were performed in compliance with the Guide for the Care and Use of Laboratory Animals of the National Institutes of Health and the Animal Welfare Act Regulations.

### Synthesis and characterization of HTL

Synthesis of LA-TK conjugate: Under a nitrogen (N_2_) atmosphere, LA (210 mg, 1 mmol) was dissolved in anhydrous dichloromethane, followed by sequential addition of EDC·HCl (200 mg, 2 mmol) and NHS (230 mg, 2 mmol) with 30-min stirring for activation. A methanol solution (5 ml) containing TK-NH_2_ (300 mg, 1.5 mmol) was then slowly added dropwise, and the reaction mixture was stirred continuously at room temperature under N_2_ protection for 48 h. After completion, the product was purified by ether precipitation (4 °C, 12 h) to yield a white solid LA-TK conjugate. Synthesis of HTL: HA (75 mg) was swollen in deionized water, lyophilized, and redispersed in N, N-dimethylformamide. EDC·HCl (200 mg, 2 mmol) and NHS (230 mg, 2 mmol) were added sequentially and activated for 1 h. Subsequently, a dichloromethane solution (5 ml) of LA-TK conjugate (150 mg, 0.4 mmol) was slowly added dropwise, followed by ultrasonication (100 W, 10 min) to homogenize the system. After 24 h of reaction, the crude product was purified by dialysis (molecular weight cutoff of 3,500 Da, aqueous dialysis at 4 °C, water changed every 6 h for 48 h) and lyophilized to obtain HTL.

Additionally, 2 control compounds were synthesized using the same strategy as HTL: (a) HA-TK-OA (HTO), where OA replaced LA; and (b) HA-LA (HL), formed via direct amidation of HA with LA. The synthesis and purification of all control compounds were performed according to the procedures detailed in the Supplementary Materials. All intermediates and final products were characterized by proton nuclear magnetic resonance (^1^H-NMR) (JNM-ECZ400S/L1 NMR spectrometer, JEOL). The LA-TK intermediate was further confirmed by Fourier-transform infrared spectroscopy (Great 20 spectrometer, Rigaku) and high-resolution mass spectrometry (Q Exactive Plus, Thermo Fisher). The critical micelle concentration of the graft copolymer was determined using the pyrene fluorescence probe method (experimental details in the Supplementary Materials). The degree of substitution was calculated based on sulfur content determined by elemental analysis. Since native HA contains no sulfur, the sulfur in the graft copolymer originated from the TK linker and LA units, with degree of substitution defined as the mass ratio of sulfur-containing side chains to the HA backbone per unit mass.

### Studies on the antioxidant performance of HTL

This study evaluated the antioxidant capacity of HTL by assessing its scavenging effects on hydroxyl radicals (·OH) and superoxide anions (·O_2_^−^). The antioxidant performance of HTL was systematically examined using the TMB colorimetric method and crystal violet staining assay. The TMB colorimetric method for evaluating ·OH scavenging activity was conducted as follows: Different concentrations (0.1 to 2.0 mg/ml) of LA (dissolved in dimethyl sulfoxide [DMSO]) or HTL/HTO solutions containing equivalent molar amounts of LA were tested. Taking the 0.1 mg/ml LA group as an example, 3 parallel experiments were set up: (a) Experimental group: 200-μl LA + H_2_O_2_/FeCl_3_ + TMB; (b) Sample control group: 200-μl LA + H_2_O_2_/FeCl_3_ + DMSO; (c) Blank control group: 200-μl DMSO + H_2_O_2_/FeCl_3_ + TMB. After incubating the reaction system at 37 °C for 20 min, the absorbance was measured at 450 nm. The ·OH scavenging rate (*I*) was calculated using the following formula:

$$I\;\left(\%\right)=\frac{A_{0}-\left(A-A_{i}\right)}{A}\times 100\%$$ (1)

where *A*, *A_i_*, and *A*_0_ represent the absorbance values of the sample group, control group, and blank control group, respectively. The same method was applied to other concentration groups.

The crystal violet staining method for evaluating ·OH scavenging capacity was performed as follows: HTL solutions with concentration gradients of 0.01 to 1 mg/ml (calculated based on LA content in HTL) were prepared. The standard reaction system consisted of 500 μl of 0.4 mmol/l FeCl_3_, 500 μl of 0.4 mmol/l H_2_O_2_, and 200 μl of HTL solution at different concentrations. After vortex mixing for 1 min and static incubation for 10 min, 100 μl of 0.5 mg/ml crystal violet solution was added. The mixture was then continuously monitored at 37 °C for 30 min, with absorbance at 580 nm recorded every 3 min. Time-absorbance kinetic curves were plotted to characterize the antioxidant activity of HTL.

Furthermore, to evaluate the ·O_2_^−^ scavenging capacity of HTL, this study employed the riboflavin-nitroblue tetrazolium (NBT) photoreduction method. A concentration gradient of HTL (0.01 to 1 mg/ml, calculated based on LA content in the molecular structure) was tested. All reaction components were prepared in 0.1 mol/l phosphate-buffered saline (PBS) buffer (pH 7.4), including riboflavin (0.4 mg/ml), methionine (9.5 mg/ml), EDTA disodium (16.8 mg/ml), and NBT (1 mg/ml). The experimental procedure was as follows: In a 5-ml Eppendorf tube, 500 μl each of riboflavin, methionine, and EDTA-disodium solutions was sequentially added, followed by 200 μl of NBT solution and 200 μl of HTL solution at different concentrations. The reaction system was exposed to light-emitting diode light (4,000 lux), and the absorbance at 600 nm was measured at 0, 3, 6, 9, 12, 15, and 18 min. To further analyze the reaction process, ultraviolet-visible absorption spectra in the range of 500 to 750 nm were additionally recorded at 15 min to dynamically monitor the reduction of NBT through changes in characteristic absorption peaks.

### Preparation and characterization of IAA@HTL nanoparticles

IAA@HTL nanoparticles (NPs) were prepared using the nanoprecipitation method with the following specific steps: IAA and HTL were separately dissolved in tetrahydrofuran to prepare stock solutions at 400 μg/ml; appropriate volumes of the solutions were mixed according to a predetermined drug-to-polymer ratio (m_IAA_:m_HTL_) and then slowly dripped (15 drops/min) into deionized water maintained at a constant temperature. After continuous stirring for 1 h, the mixture was transferred to a rotary evaporator (50 °C, 15 min) to remove the organic solvent, followed by filtration through a 0.22-μm microporous membrane to obtain the IAA@HTL NP solution. A single-factor experimental design was systematically employed to investigate the effects of drug-to-polymer ratio (m_IAA_:m_HTL_), hydration volume, and hydration temperature on NPs’ properties, with particle size distribution, polydispersity index (PDI), and zeta potential as evaluation indicators. The morphology of the NPs was observed using transmission electron microscopy (Talos F200X, ThermoFisher Scientific), while particle size and surface potential were measured using a Malvern Zetasizer Nano ZS90. The crystal structure and thermodynamic properties were characterized by x-ray diffraction (XRD, XRD-6100 X, Shimadzu) and differential scanning calorimetry (DSC, Q2000, TA Instruments), respectively.

### Encapsulation efficiency and loading capacity

The drug loading performance of IAA@HTL NPs was determined using high-performance liquid chromatography (HPLC, LC-2010A High-performance Liquid Chromatograph, Shimadzu). The specific procedure was as follows: 0.5 ml of the drug-loaded NP solution was mixed with an equal volume (0.5 ml) of chromatographic methanol, vortexed for 10 min, and then filtered through a 0.22-μm microporous membrane to prepare the test sample. The IAA concentration was measured according to the established and validated HPLC method (method validation details are provided in the Supplementary Materials). The encapsulation efficiency (EE [%]) and drug loading capacity(DL [%]) were calculated using the following formulas:

$$\mathrm{EE}\;\left(\%\right)=\frac{\text{Encapulated}\;\mathrm{IAA}\;\text{content}}{\text{Total}\;\mathrm{IAA}\;\text{input}}\times 100\%$$ (2)

$$\mathrm{LC}\;\left(\%\right)=\frac{\text{Encapulated}\;\mathrm{IAA}\;\text{content}}{\text{Weight of nanoparticles}}\times 100\%$$ (3)

### Plasma stability and in vitro release behavior of IAA@HTL NPs

In the plasma stability study, IAA@HTL NP solution was mixed with equal volumes of either PBS buffer (pH 7.4) containing 20% FBS or plain PBS buffer (pH 7.4), followed by incubation at 37 °C for 12 h. Samples were collected at 0, 2, 4, 6, 8, 10, and 12 h, and NP size and PDI were determined by dynamic light scattering to systematically evaluate their stability in simulated physiological conditions, with IAA@HTO NPs (HTO as carrier) and IAA@HL NPs (HL as carrier) serving as controls. For the in vitro release study, the drug release profiles of IAA@HTL NPs and control formulations (IAA@HTO NPs and IAA@HL NPs) were investigated using dialysis in 3 different release media: (a) simulated physiological environment (pH 7.4 PBS buffer); (b) simulated oxidative environment (pH 7.4 PBS buffer containing 10 mmol/l H_2_O_2_); and (c) simulated reductive environment (pH 7.4 PBS buffer containing 10 mmol/l GSH). At predetermined time points (0, 1, 2, 4, 6, 8, 12, and 24 h), 200 μl of release medium was precisely sampled and replaced with an equal volume of fresh medium to maintain sink conditions. All samples were filtered through 0.22-μm microporous membranes, and IAA concentration was quantified using a validated HPLC method (chromatographic conditions detailed in the Supplementary Materials) to calculate the cumulative release percentage.

### Biocompatibility of IAA@HTL NPs

#### Cell cytotoxicity assessment

In the cytotoxicity study, HT-29 and RAW264.7 cells were used as models for intestinal epithelial cells and macrophages, respectively, and the cytotoxicity of graft copolymers (HTL, HTO, and HL) and their drug-loaded NPs (IAA@HTL, IAA@HTO, and IAA@HL) was evaluated using the MTT assay. Cells in the logarithmic growth phase were seeded in 96-well plates at a density of 5 × 10^3^ cells/well and precultured for 24 h at 37 °C with 5% CO_2_ to allow cell attachment. The medium was then replaced with fresh medium containing different concentrations of graft copolymers (based on material mass concentration) or drug-loaded NPs (based on IAA loading amount). After 24 h of incubation, 200 μl of MTT solution (0.5 mg/ml in PBS) was added to each well and incubated for 4 h to form purple formazan crystals. The supernatant was discarded, and 150 μl of DMSO was added to dissolve the crystals. The absorbance (optical density [OD]) was measured at 490 nm using a microplate reader. Cell viability was calculated using the following formula:

$$\text{Cell viability}\;\left(\%\right)=\frac{{\mathrm{OD}}_{\text{Sample}-}{\mathrm{OD}}_{\text{Blank}}}{{\mathrm{OD}}_{\text{Nagative}-}{\mathrm{OD}}_{\text{Blank}}}\times 100\%$$ (4)

where OD_Blank_ represented the optical density of the blank group (no cells), OD_Negative_ was the optical density of the negative control group (untreated cells), and OD_Sample_ was the optical density of the treatment groups.

#### Hemolysis analysis

Rat whole blood (10 ml) was taken, centrifuged (2,000 r/min, 5 min), to obtain the lower layer of red blood cells (RBCs). The RBCs were washed with physiological saline and centrifuged (2,000 r/min, 5 min, repeated 3 times) to remove plasma proteins and platelets. The purified RBCs were then resuspended in pH 7.4 PBS buffer to prepare an RBC suspension. The experiment included 3 kinds of parallel control groups: a negative control group (PBS buffer), a positive control group (1% Triton X-100), and experimental groups (different concentrations of IAA@HTL, IAA@HTO, or IAA@HL NP solutions). The RBC suspension (500 μl) was mixed with an equal volume of test samples and incubated at 37 °C in a constant-temperature water bath for 4 h to ensure sufficient contact between RBCs and test substances. After incubation, the mixture was centrifuged (2,000 r/min, 5 min), and the absorbance of the supernatant was measured at 570 nm. The hemolysis rate (HR) was calculated as follows:

$$\mathrm{HR}\;\left(\%\right)=\frac{A_{\text{Sample}-}A_{\text{Negative}}}{A_{\text{positive}-}A_{\text{Negative}}}\times 100\%$$ (5)

where *A*_Sample_ was the absorbance of the experimental group, *A*_Negative_ was the absorbance of the PBS group, and *A*_Positive_ was the absorbance of the Triton X-100 group.

#### Hematological and biochemical analyses

Healthy male ICR mice were administered IAA@HTL, IAA@HTO, or IAA@HL NP aqueous solutions (IAA-equivalent dose: 80 μg/kg) via tail vein injection every other day for 14 consecutive days. After the treatment period, the mice were euthanized under anesthesia, and blood samples were collected to evaluate hepatic and renal function and hematological parameters. Major organs (heart, liver, spleen, lungs, and kidneys) were harvested, fixed in 4% paraformaldehyde, and subjected to hematoxylin-eosin (H&E) staining for histopathological examination.

### Cellular uptake and intracellular ROS scavenging

In the cellular uptake experiment, R123-labeled HTL NPs (R123@HTL) were used to investigate the uptake differences of NPs by intestinal epithelial cells and macrophages under normal and inflammatory conditions, using HT-29 cells and RAW264.7 cells as models. Cells were seeded at a density of 2 × 10^5^ cells/well in 6-well plates and cultured for 24 h at 37 °C with 5% CO_2_ to ensure adherence, followed by grouping and treatment. The experiment included a blank control group, a free R123 group (Free R123), and an R123@HTL group, which only received fresh medium. For the inflammatory model groups (LPS+R123, LPS+R123@HTL, and HA-blocked), cells were prestimulated with 100 ng/ml LPS for 12 h to establish the inflammatory model, with the HA-blocked group additionally pretreated with 1 mg/ml HA for 12 h for receptor blocking. Subsequently, the Free R123 and LPS+R123 groups were treated with free R123 solution, while the other experimental groups received R123@HTL NPs. After 4 h of incubation, cells were washed 3 times with precooled PBS, fixed with 4% paraformaldehyde for 15 min, and stained with Hoechst 33342 (1 μg/ml) for 10 min for nuclear labeling. Cellular uptake was observed and recorded using an inverted fluorescence microscope (IX71 inverted fluorescence microscope, Olympus, excitation/emission wavelengths: 507/529 nm).

To evaluate the ROS-scavenging ability of the nanodrug delivery system, the oxidative stress levels in HT-29 and RAW264.7 cells were detected using the DCFH-DA fluorescent probe. Log-phase cells were seeded at 1 × 10^5^ cells/well in 6-well plates and cultured for 24 h at 37 °C with 5% CO_2_, then randomly divided into 6 groups: blank control, free IAA (16 μg/ml), blank HTL NPs, IAA@HTL, IAA@HTO, and IAA@HL NPs (the drug-loaded groups were prepared at equivalent concentrations of free IAA). All groups were treated with 200 μmol/l H_2_O_2_ to induce ROS production. After 12 h, except for the control group, the other groups were treated with the corresponding preparations for 3 h. The medium was discarded, and cells were incubated with 10-μmol/l DCFH-DA working solution in the dark for 30 min, washed 3 times with precooled PBS, and immediately observed under an inverted fluorescence microscope (excitation/emission wavelengths: 488 nm/525 nm) to record changes in intracellular fluorescence intensity.

### Assessment of apoptosis inhibition

This study systematically evaluated the inhibitory effects of drug-loaded NPs on LPS-induced apoptosis in HT-29 cells through multidimensional experiments. First, the protective effect of NPs was assessed using the MTT assay: HT-29 cells in the logarithmic growth phase (5 × 10^3^ cells/well) were seeded in 96-well plates, and after 24 h, they were divided into 2 experimental groups: (a) comparing the protective effects of different NP formulations (IAA@HTL, IAA@HTO, IAA@HL, and blank HTL NPs, all prepared at equivalent IAA concentrations) under 1 μg/ml LPS stimulation; and (b) examining the concentration-dependent effect of IAA@HTL NPs (0.1 to 10 μg/ml IAA equivalent concentration). After 12 h of treatment, cell viability was measured using the MTT assay, and absorbance at 490 nm was detected using a microplate reader to calculate survival rates.

To further validate the protective effects, morphological observation was performed using calcein-AM/PI double staining. HT-29 cells were seeded in 12-well plates at a density of 1 × 10^5^ cells/ml and cultured for 24 h before being divided into 2 groups: a normal control group and an LPS-induced group (containing 1 μg/ml LPS). Each group was treated with free IAA, blank HTL NPs, IAA@HTL, IAA@HTO, or IAA@HL (NP concentrations were calculated based on equivalent IAA concentrations), with a blank medium control also included. After 12 h of treatment, calcein-AM/PI double staining was performed, and the distribution of live (green) and dead (red) cells was observed under an inverted fluorescence microscope.

Finally, the inhibitory effect of drug-loaded NPs on LPS-induced apoptosis in HT-29 cells was quantitatively assessed using Annexin V-FITC/PI double staining combined with flow cytometry. HT-29 cells in the logarithmic growth phase were seeded in 6-well plates at a density of 2 × 10^5^ cells/ml and incubated for 24 h at 37 °C with 5% CO_2_. Seven treatment groups were established: blank control, LPS model group, free IAA group, blank HTL NP group, IAA@HTL NP group, IAA@HTO NP group, and IAA@HL NP group. Except for the control group, all other groups were pretreated with medium containing 1 μg/ml LPS for 2 h to establish an inflammatory cell model. Subsequently, the corresponding treatments—IAA (10 μg/ml), HTL (equivalent carrier amount), IAA@HTL, IAA@HTO, and IAA@HL (all prepared at an equivalent IAA concentration of 10 μg/ml)—were added and incubated for an additional 10 h. The medium and trypsin-digested cell suspensions were combined and centrifuged (1,000 rpm, 5 min), followed by staining using an Annexin V-FITC/PI apoptosis detection kit. Apoptosis was analyzed using a flow cytometer (BD FACSCalibur), and experimental data were processed with FlowJo 10.6 software.

### Assessment of macrophage polarization and inflammatory cytokine modulation

To evaluate the regulatory effects of drug-loaded NPs on macrophage polarization and inflammatory cytokine expression, RAW 264.7 cells in the logarithmic growth phase were seeded in 6-well plates at a density of 2 × 10^5^ cells/well and cultured for 24 h at 37 °C with 5% CO_2_. After incubation, the cells were divided into 7 groups: control group, LPS group, IAA group, HTL group, IAA@HTL group, IAA@HTO group, and IAA@HL group. The control group was cultured in complete medium for an additional 24 h, while the other groups were treated with 2 ml of 200 ng/ml LPS for 8 h, followed by replacement with medium containing corresponding formulations (drug concentrations calculated based on IAA equivalents) for another 4 h. For macrophage polarization analysis, cells were coincubated with phycoerythrin rat anti-mouse CD86 and allophycocyanin rat anti-mouse CD206 antibodies at room temperature for 15 min, and fluorescence signals were detected using a flow cytometer (BD FACSCalibur), with data analyzed by FlowJo 10.6 software. For inflammatory cytokine expression, RAW 264.7 cells were treated using the same culture and grouping methods, and the supernatant was collected to measure TNF-α, IL-1β, IL-10, and TGF-β1 levels using ELISA kits. The same approach was applied to assess the effect of IAA@HTL on inflammatory cytokine expression in HT-29 cells.

Since IAA@HTL exerts its anti-inflammatory effects primarily through IAA and LA, this study further investigated the synergistic effect of their combination against inflammation. Using RAW264.7 cells as a model and the proinflammatory cytokine TNF-α as the detection indicator, the pharmacodynamic evaluation was performed using the Chou–Talalay combination index (CI) method [24]. The experimental groups included a blank control group, an LPS model group (1 μg/ml), IAA monotherapy groups, LA monotherapy groups (with concentration gradients of 3, 6, 12, 24, 48, and 96 μM for both IAA and LA), and IAA+LA combination groups (at a fixed molar ratio of 1:1, with the same concentrations as above). Cells in each group were pretreated with LPS for 2 h, followed by coincubation with the respective drugs for 24 h. Cell supernatants were collected, and TNF-α levels were detected using ELISA. The inhibition rate was calculated using the following formula:

$$\text{Inhibition Rate}\;\left(\%\right)=\frac{C_{\mathrm{LPS}-}C_{\text{Treatment}}}{C_{\mathrm{LPS}-}C_{\text{Blank}}}\times 100\%$$ (6)

where *C*_LPS_ represented the TNF-α concentration in the LPS group, *C*_treatment_ represented the TNF-α concentration in each treatment group, and *C*_Blank_ represented the TNF-α concentration in the blank control group. Based on the dose-effect data, the CI was calculated using the CompuSyn software (Version 1.4, ComboSyn, Inc., Paramus, NJ, USA) to evaluate the nature of the combined effect of the 2 drugs. CI < 0.9 indicates a synergistic effect, CI = 0.9 to 1.1 indicates an additive effect, and CI > 1.1 indicates an antagonistic effect.

### In vivo biodistribution evaluation

This study utilized the fluorescent molecule R123 as a substitute for IAA to label NPs (R123@HTL NPs) for evaluating the biodistribution characteristics of drug-loaded NPs in both healthy and UC model mice. Male ICR mice were randomly divided into a healthy control group and a UC model group. The model group received 3% (w/v) dextran sodium sulfate (DSS) solution orally for 7 consecutive days to induce inflammation. After successful modeling, R123@HTL NP suspension (100 μg/kg R123 equivalent dose) was administered via tail vein injection. At predetermined time points postinjection (0, 2, 4, 6, and 24 h), animals were euthanized, and major organs (heart, liver, spleen, lungs, kidneys, and colon) were collected. Ex vivo organ fluorescence imaging was performed using a small animal in vivo imaging system (Lumazone FA2048, Teledyne Photometrics, USA), followed by semiquantitative analysis of fluorescence intensity using ImageJ software.

### Pharmacokinetic study

Male Sprague–Dawley rats (220 to 250 g) were randomly divided into a free IAA group and an IAA@HTL group, with 6 rats in each group. The rats were administered 0.5 mg/kg of the corresponding drug via tail vein injection. Blood samples were collected from the jugular vein at predetermined time points postadministration, and plasma was separated by centrifugation. Plasma samples were pretreated by protein precipitation with methanol, followed by centrifugation, evaporation to dryness under nitrogen, and reconstitution. Drug concentrations were determined using HPLC. The chromatographic conditions were as follows: a BDS Hypersil C18 column, isocratic elution with methanol-2% acetic acid aqueous solution (40:60, v/v), detection wavelength of 280 nm, column temperature of 25 °C, flow rate of 1.0 ml/min, and injection volume of 20 μl. To ensure the reliability of the analytical method, systematic methodological validation was performed, including assessments of specificity, standard curve and linear range, intraday and interday precision, recovery rate, and sample stability. In this work, the Phoenix WinNonlin software (v8.1, Certara, USA) was used for the calculation of pharmacokinetic parameters.

### In vivo intervention of IAA-loaded NPs on DSS-induced colitis in mice

#### Establishment and treatment process of DSS-induced colitis model

Six-week-old male ICR mice were used to establish UC animal models. The normal control group received sterile filtered water, while the model group was given 3% DSS water solution for 7 d, followed by a return to regular drinking water. The model group mice were then divided into 4 subgroups: DSS model group, free IAA group, blank HTL NP group, and IAA@HTL NP group, with 6 mice in each group. All groups were administered via tail vein injection, with the first dose given on the day after modeling completion and subsequent doses administered every other day for a total of 3 doses. The control and DSS model groups received 0.1 ml of saline, while the remaining 3 treatment groups received 0.1 ml of freshly prepared free IAA aqueous solution (80 μg/ml), blank HTL NP suspension, or IAA@HTL NP suspension (the concentration of IAA@HTL NPs was calculated based on the equivalent free IAA concentration, and blank HTL NPs were adjusted according to the mass ratio of HTL in IAA@HTL NPs). Body weight changes were recorded throughout the experiment. Disease activity index (DAI) was assessed starting from the first day of DSS treatment, with scores calculated based on weight loss (0 to 4 points), fecal blood (0 to 4 points), and stool consistency (0 to 4 points). On day 15, blood was collected via orbital bleeding before euthanasia, and major organs (heart, liver, spleen, lungs, kidneys, and colon) were rapidly isolated. The colon tissue was rinsed with PBS, photographed, and measured for length. The spleen was weighed to calculate the spleen index (spleen weight/body weight × 100%) as an indicator of systemic inflammatory response.

#### Histological and immunofluorescence assay

The collected organ tissue samples were fixed in 4% paraformaldehyde, embedded in paraffin, and sectioned into 4-μm-thick slices. H&E staining was first performed to observe histopathological changes. To further analyze microenvironmental alterations in colon tissues, the following assays were conducted: (a) immunofluorescence double staining using fluorescently labeled anti-CD86 (M1 marker) and anti-CD206 (M2 marker) antibodies to assess macrophage polarization status; (b) dihydroethidium (DHE) staining to detect superoxide anion (·O_2_^−^) levels, indirectly reflecting ROS generation; (c) immunofluorescence staining with ZO-1 and Occludin antibodies for TJs and E-cadherin antibody for adherens junctions (AJs), combined with fluorescent secondary antibodies, to evaluate epithelial-barrier function. All fluorescence staining results were imaged using a confocal laser scanning microscope, and fluorescence intensity was quantitatively analyzed using ImageJ software.

#### Oxidative stress and cytokine assay

Colon tissue samples were homogenized in precooled 50 mM PBS buffer (pH 6.0) at a 1:10 (w/v) ratio, followed by centrifugation at 10,000 g for 10 min at 4 °C to collect the supernatant. Blood samples were allowed to stand at room temperature for 1 h and then centrifuged at 3,000 rpm for 10 min at 4 °C to separate the serum, which was stored at −80 °C. To evaluate oxidative stress in colon tissues, myeloperoxidase (MPO), malondialdehyde (MDA), and superoxide dismutase (SOD) levels in the homogenate supernatant were measured using commercial assay kits. Meanwhile, the expression levels of cytokines, including TNF-α, IL-1β, IL-10, TGF-β1, and IL-22, in both colon tissue homogenate supernatant and serum were determined using ELISA kits.

#### Western blotting

Colon tissue samples were homogenized in radioimmunoprecipitation assay lysis buffer containing phenylmethylsulfonyl fluoride and phosphatase inhibitors to extract total proteins, with protein concentrations determined using bicinchoninic acid protein assay kits. Following the literature method [25], Western blotting was performed to detect Nrf2, HO-1, AhR, CYP1A1, and Caspase-3 expression. Protein bands were visualized using a Clinx chemiluminescence imaging system (ChemiScope 6100, Clinx, China), and grayscale quantification analysis was conducted with ImageJ software.

### Gut microbiota analysis

The gut microbiota in mouse feces was analyzed using 16S ribosomal RNA V3-V4 region sequencing technology. After genomic DNA extraction, polymerase chain reaction amplification, fluorescence quantification, and library construction, sequencing was performed on the NovaSeq platform (completed by Novogene Co., Ltd., Beijing). Cloud-based analysis was conducted to examine microbial composition, α-diversity, and species abundance, with Spearman correlation analysis performed using the corr.test function from the R psych package.

### Statistical analysis

Statistical significance was evaluated using 1-way analysis of variance (1-way ANOVA), followed by post hoc pairwise comparisons using the least significant difference method when significant differences were detected. A *P* value <0.05 was considered statistically significant. All experiments were performed with at least 3 independent replicates, and data were expressed as means ± SD.

## Results and Discussion

### Synthesis, characterization, and antioxidant analysis of graft copolymers

**Fig. 2.**
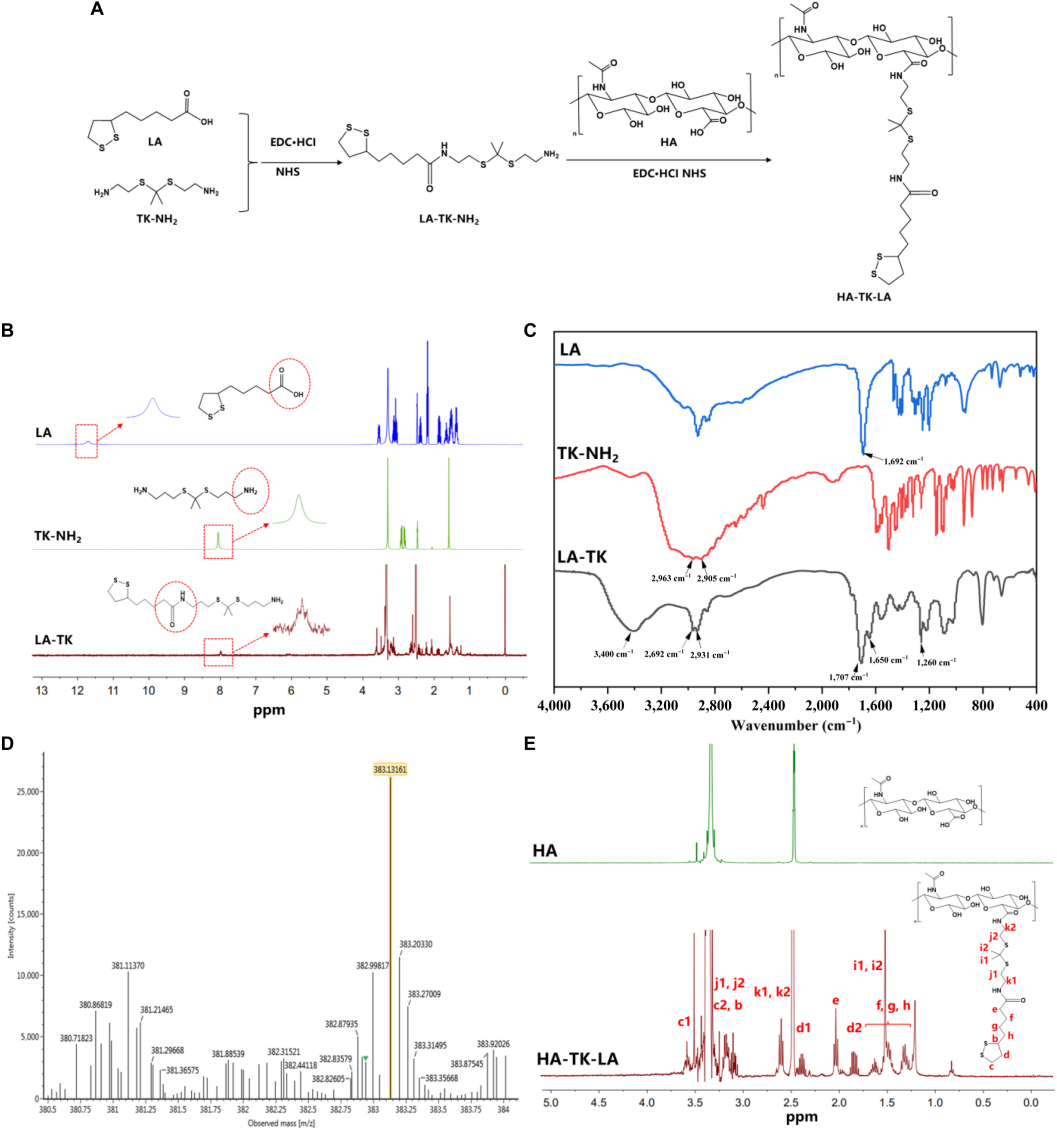
Synthesis and characterization of the amphiphilic graft copolymer hyaluronic acid-thioketal-lipoic acid (HA-TK-LA [HTL]). (A) Schematic illustration of the HTL synthesis route. (B) Full-range (0 to 13 ppm) proton nuclear magnetic resonance (^1^H-NMR) spectra of lipoic acid (LA), thioketal amine (TK-NH_2_), and the LA-TK intermediate. (C) Fourier-transform infrared (FTIR) spectra of LA, TK-NH_2_, and LA-TK, with arrows indicating key functional group vibration peaks. (D) Electrospray ionization mass spectrometry (ESI-MS) of the LA-TK intermediate. (E) ^1^H-NMR spectra (0 to 5 ppm) of hyaluronic acid (HA) and HTL.

The amphiphilic graft copolymer HTL was constructed using HA as the hydrophilic backbone, which was linked to the hydrophobic LA via a ROS-responsive TK bond. The synthesis proceeded in 2 steps: First, the carboxyl group (–COOH) of LA and the amino group (–NH_2_) of TK underwent amidation catalyzed by EDC·HCl/NHS to form the intermediate LA-TK. Subsequently, under the same activation system, the –COOH of HA further reacted with the –NH_2_ of LA-TK via acylation to yield HTL (Fig. 2A). The structure of the intermediate LA-TK was systematically characterized using multiple spectroscopic techniques. ^1^H-NMR analysis confirmed the successful synthesis of LA-TK: Comparison of the spectra of LA, TK-NH_2_, and LA-TK revealed the disappearance of the –COOH (12 ppm) and –NH_2_ characteristic peaks, while a new triplet (t) appeared at 8 ppm, assigned to the amide proton, indicating the occurrence of amidation (Fig. 2B). The methylene protons (–CH_2_–) of TK-NH_2_ originally at 2.84 to 2.98 ppm shifted to 2.59 ppm (m, 4H) and 3.0 to 3.5 ppm in LA-TK, accompanied by the emergence of characteristic proton signals from LA, including methylene protons at 1.32 to 1.62 ppm, dithiolane methylene protons at 2.37/1.84 ppm, and methine/methylene protons at 3.09 to 3.56 ppm (Fig. S1). Fourier-transform infrared spectroscopy further supported these findings: The C=O stretching vibration of LA at 1,692 cm^−1^ and the N–H absorption peaks of TK-NH_2_ at 2,963/2,905 cm^−1^ disappeared, while characteristic amide absorptions emerged, including N–H stretching at 3,400 cm^−1^, C=O stretching at 1,707 cm^−1^, and amide I and II bands at 1,650 and 1,260 cm^−1^, respectively (Fig. 2C). Additionally, high-resolution mass spectrometry showed a molecular ion peak [M+H]^+^ at a mass/charge ratio of 383.13161 in positive ion mode, which was highly consistent with the theoretical molecular weight (382.13137), further confirming the structure of LA-TK (Fig. 2D). The structure of HTL was verified by ^1^H-NMR: The methine and methylene signals of the HA backbone at 3.0 to 3.6 ppm remained, alongside the characteristic proton peaks of the LA-TK side chains, including 3.57 ppm (H-c1), 3.04 to 3.18 ppm (H-c2/H-b/H-j1/j2), 2.59 ppm (H-k1/k2), 2.39/1.83 ppm (H-d), 2.06 ppm (H-e), and 1.28 to 1.64 ppm (H-f/g/h/H-i1/i2), indicating successful grafting of HTL (Fig. 2E). To further investigate the structure–property relationship of the copolymers, we synthesized 2 control compounds: HTO (using OA instead of LA) and HL (HA directly linked to LA without the TK linkage) (Fig. S2). Their detailed characterization data are provided in the Supplementary Materials (Figs. S3 to S5). Elemental analysis revealed sulfur contents of 11.13%, 5.81%, and 7.61% for HTL, HTO, and HL, respectively, corresponding to grafting degrees of 49.46%, 48.60%, and 59.79%. Pyrene fluorescence assays indicated that the critical micelle concentration of all 3 copolymers was below 0.03 mg/ml, demonstrating their ability to self-assemble into micellar structures (composed of amphiphilic copolymers) even at very low concentrations, thereby providing a foundation for the subsequent construction of IAA-loaded NPs (Fig. S6).

**Fig. 3.**
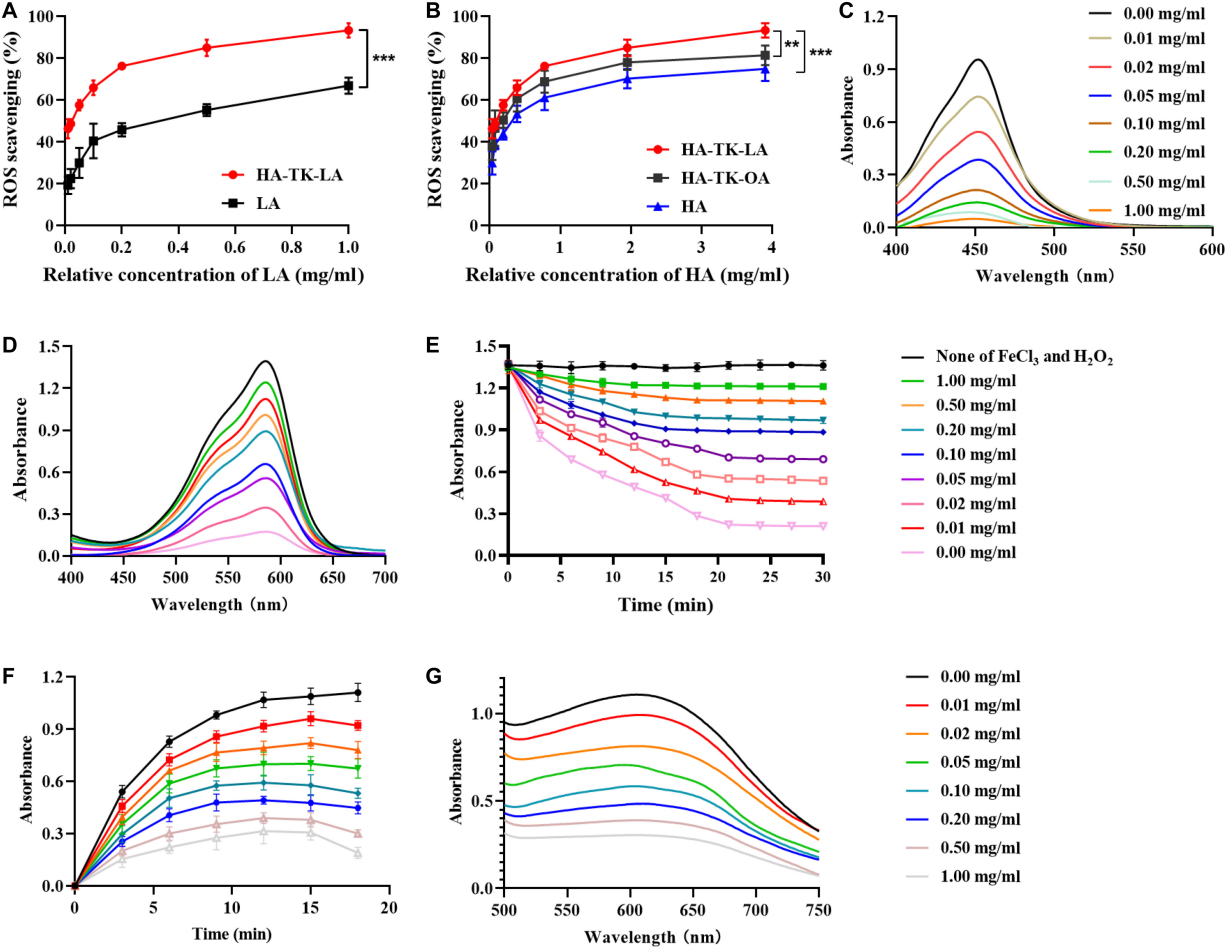
Evaluation of reactive oxygen species (ROS) scavenging capacity of HA-TK-LA (HTL). (A) Comparison of ROS scavenging efficiency between lipoic acid (LA) and HTL. (B) ROS scavenging ability comparison among HTL, HA-TK-OA (HTO), and hyaluronic acid (HA). (C) Effect of different HTL concentrations on the absorbance (400 to 600 nm) of the FeCl_3_/H_2_O_2_-TMB (3,3’,5,5’-tetramethylbenzidine) system (ultraviolet [UV]-visible [Vis] absorption spectra). (D) Effect of different HTL concentrations on the absorbance (400 to 700 nm) of the FeCl_3_/H_2_O_2_-crystal violet system. (E) Time-dependent changes in absorbance at 580 nm for crystal violet under HTL concentration gradients. (F) Detection of superoxide anion (·O_2_^−^) scavenging by HTL using the riboflavin photoreduction method: Time-dependent changes in absorbance at 600 nm for nitroblue tetrazolium chloride (NBT) after treatment with different HTL concentrations. (G) UV-Vis absorption spectral changes (500 to 750 nm) of NBT under HTL concentration gradients. Data are presented as means ± SD (*n* = 3). ^**^P < 0.01, ^***^P < 0.001. m/s, mass/charge ratio.

In vivo, LA can be converted to dihydrolipoic acid, and both act synergistically to enhance the scavenging capacity for various reactive oxygen/nitrogen species [26]. Therefore, this study evaluated the ROS scavenging performance of the LA derivative, HTL. First, its ability to scavenge ·OH was investigated. During UC progression, ·OH is primarily generated through activated neutrophils, macrophage polarization, mitochondrial dysfunction in epithelial cells, and the Fenton reaction triggered by intestinal pathogens, collectively contributing to intestinal barrier disruption, aggravated inflammation, and dysbiosis of the gut microbiota. In this study, a Fenton reaction system (FeCl_3_/H_2_O_2_) was employed to generate ·OH, with TMB as the chromogenic agent, and ·OH levels were quantified by measuring absorbance changes at 450 nm. At equivalent LA concentrations, HTL exhibited superior concentration-dependent ·OH scavenging efficacy compared to LA (Fig. 3A). Furthermore, when comparing different polymers (HTL, HTO, and HA) based on HA concentration, all 3 polymers demonstrated concentration-dependent enhancement in ·OH scavenging, with HTL performing markedly better than HTO and HA (Fig. 3B). TMB chromogenic assays visually confirmed gradual fading of the yellow color with increasing polymer concentrations, with HTL showing the most pronounced fading effect (Fig. S7). Ultraviolet-visible spectral analysis further verified a positive correlation between HTL concentration and the reduction in absorbance at 450 nm (Fig. 3C). These results indicate that HA possesses weak ROS scavenging activity, possibly attributable to the electron/hydrogen-donating ability of its glucuronic acid units, whereas HTL integrates the antioxidative properties of both LA and HA, leading to a synergistic enhancement in ·OH scavenging capacity [27]. Experiments using crystal violet also confirmed that HTL could dose-dependently restore the ·OH-induced reduction in absorbance (Fig. 3D), with sustained scavenging activity observed over 30 min (Fig. 3E). Additionally, the ·O_2_^−^ scavenging ability of HTL was evaluated. In intestinal inflammation, epithelial cells generate ·O_2_^−^ due to electron leakage from mitochondrial electron transport chain complexes I/III dysfunction, initiating mitochondrial ROS cascades [28]. A riboflavin photoreduction/NBT chromogenic assay was used, quantifying ·O_2_^−^ levels via formazan absorbance at 600 nm [29]. As shown in Fig. 3F, compared to the control group, HTL treatment (especially at higher concentrations) markedly suppressed the increase in absorbance (resulting from the reaction of ·O_2_^−^ generated by riboflavin with NBT to form blue formazan). Upon termination of illumination, a rapid decline in absorbance was observed, indicating effective clearance of ·O_2_^−^ in the system. Quantitative analysis further confirmed the concentration-dependent ·O_2_^−^ scavenging effect of HTL (Fig. 3G). By efficiently eliminating ·O_2_^−^ and ·OH, HTL holds promise for clearing excessive ROS in the inflammatory intestinal mucosa, thus laying the foundation for alleviating inflammation and repairing the mucosa.

### Preparation and characterization of IAA-loaded NPs

**Fig. 4.**
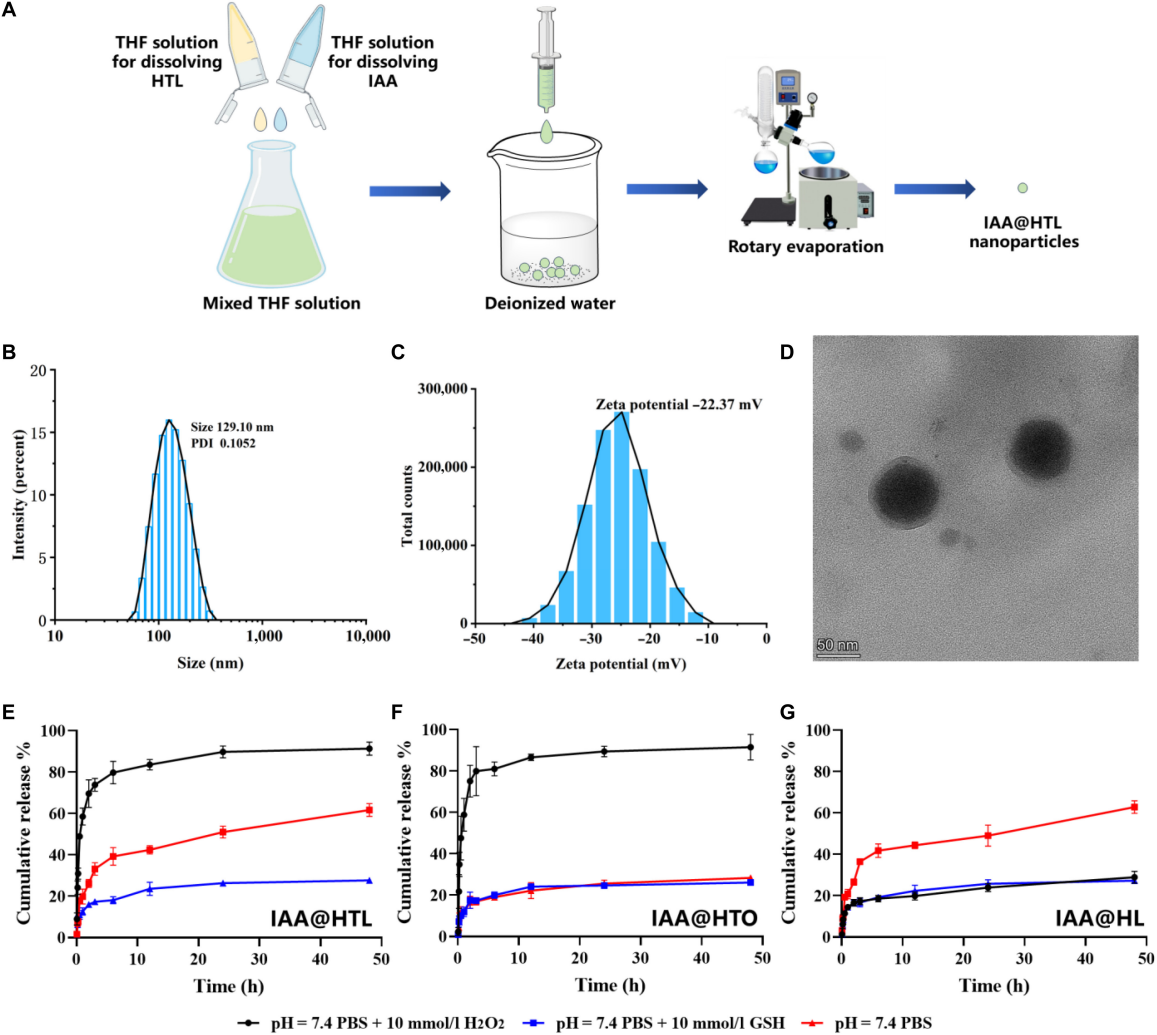
Construction, characterization, and in vitro release study of IAA@HTL nanoparticles (NPs). (A) Schematic illustration of the preparation process for IAA@HTL NPs. (B) Hydrodynamic size distribution of IAA@HTL NPs. (C) Zeta potential of IAA@HTL NPs. (D) Transmission electron microscopy (TEM) image of IAA@HTL NPs (scale bar: 200 nm). (E to G) In vitro release profiles of different nanoparticles under simulated physiological conditions: (E) IAA@HTL NPs, (F) IAA@HTO NPs, and (G) IAA@HL NPs in phosphate-buffered saline (PBS) buffer (pH 7.4) containing H_2_O_2_ (10 mM), glutathione (GSH, 10 mM), or PBS alone. Data are presented as means ± SD (*n* = 3). THF, tetrahydrofuran.

In this study, IAA@HTL NPs with a well-defined core–shell structure were fabricated through a nanoprecipitation process. This method leveraged hydrophobic interactions to drive the self-assembly of HTL, leading to the effective encapsulation of IAA (Fig. 4A) [30]. During process optimization, the effects of key parameters, including the drug-to-polymer mass ratio (m_IAA_:m_HTL_), hydration volume, and hydration temperature, were systematically investigated. As shown in Fig. S8, increasing the amount of HTL led to a decrease in drug loading efficiency, likely due to saturation of the hydrophobic core capacity. Appropriately increasing the hydration volume contributed to reduced particle size and improved drug loading, attributed to enhanced solvent diffusion rates. Hydration temperature required precise control within an optimal range, as deviations resulted in uneven particle size distribution or reduced loading efficiency. Through systematic optimization, the final preparation parameters were determined as a drug-to-polymer ratio of 1:1, hydration volume of 15 ml, and hydration temperature of 50 °C.

The IAA@HTL NPs prepared under the optimized conditions exhibited favorable physicochemical properties. Dynamic light scattering analysis showed an average hydrodynamic diameter of 130 nm with a PDI of 0.11 (Fig. 4B), indicating a narrow size distribution. The zeta potential was measured at −22 mV (Fig. 4C); this high absolute value suggests good colloidal stability and supports potential systemic circulation and targeted delivery to inflamed colon tissue via the EPR effect. Transmission electron microscopy further confirmed that the NPs exhibited a spherical morphology with a typical core–shell structure—comprising a dense hydrophobic core and a diffuse hydrophilic shell (Fig. 4D). A reliable HPLC method was developed and validated for the quantification of IAA, showing good linearity over the concentration range of 0.1 to 50 μg/ml (Figs. S9 to S11). The method also met the required standards for precision, stability, and recovery (Tables S1-S4). Using this method, the EE (%) and DL (%) of IAA@HTL NPs were determined to be 72.67% ± 1.32% and 36.34% ± 0.89%, respectively. Moreover, IAA@HTO and IAA@HL NPs prepared using the same process also exhibited satisfactory drug loading and particle size characteristics (Table S5), demonstrating the good universality of the preparation method. To investigate the physical state of the drug within the NPs, XRD and DSC tests were performed. The XRD pattern of pure IAA and the physical mixture showed sharp crystalline diffraction peaks, which completely disappeared in IAA@HTL NPs, with only the amorphous halo of HTL being observed (Fig. S12). DSC results indicated that the melting peak of IAA at 165 to 169 °C remained in the physical mixture but was absent in IAA@HTL NPs, where only the thermal response profile consistent with HTL was detected (Fig. S13). These findings confirm that IAA is molecularly dispersed in the hydrophobic core of HTL NPs in an amorphous state, with no crystalline drug phase present. This state helps reduce the crystallization energy barrier, thereby enhancing the apparent solubility of IAA. In summary, IAA@HTL NPs possess desirable physicochemical properties, drug loading performance, and structural characteristics, providing a solid foundation for subsequent in vitro and in vivo evaluations.

### Functional validation of NPs

#### Stability and responsive release analysis

For intravenously administered nanocarriers, plasma stability is critical to ensure effective drug delivery. Serum proteins tend to adsorb onto the NP surface, forming a “protein corona” that may induce particle aggregation or size alterations, thereby influencing in vivo distribution and therapeutic efficacy [31]. Therefore, evaluating the plasma stability of nanodelivery systems is essential. As shown in Figs. S14 and S15, after incubation in FBS for 12 h, IAA@HTL, IAA@HTO, and IAA@HL NPs exhibited only minor changes in particle size and PDI, indicating structural integrity under nonstimulatory conditions (i.e., without ROS/GSH) and a low tendency for premature drug leakage. Although all NPs showed a slight increase in size following FBS treatment, the final sizes remained below 150 nm, which is suitable for leveraging the EPR effect. Considering that intravenously injected NPs can reach target sites within 12 h, the delivery system demonstrates favorable plasma stability over the intended timeframe, meeting the requirements for intravenous administration.

During acute UC episodes, the inflammatory microenvironment is characterized by oxidative stress: Immune cells generate high levels of ROS via respiratory bursts, while compensatory up-regulation of GSH helps maintain redox balance [32]. This unique pathological setting provides an ideal context for designing stimulus-responsive drug delivery systems. In vitro release studies confirmed the dual ROS/GSH responsiveness of IAA@HTL NPs (Fig. 4E to G). Under simulated physiological conditions (PBS), all nanoformulations showed excellent stability, with cumulative drug release remaining below 30% over 48 h. In an inflammatory-mimicking environment (10 mM H_2_O_2_), NPs containing TK bonds (IAA@HTL and IAA@HTO) released approximately 80% of IAA within 6 h, significantly higher than the TK-lacking IAA@HL (18.50%). Similarly, in the presence of 10 mM GSH, NPs with disulfide bonds (IAA@HTL and IAA@HL) exhibited faster release (39.17% and 41.70%, respectively, at 6 h), with continued increase over 48 h, whereas disulfide-free IAA@HTO released more slowly. These results indicate that the TK bonds in HTL undergo specific cleavage in response to ROS, leading to nanostructure degradation, while the disulfide bonds in LA undergo thiolysis in the presence of GSH, altering the hydrophilic–lipophilic balance. These mechanisms collectively trigger NP disassembly and the release of both IAA and LA. This intelligent responsiveness enables the NPs to recognize the inflammatory microenvironment and achieve precise drug release, thereby enhancing therapeutic efficacy while minimizing potential systemic toxicity.

#### Cellular targeting and intracellular antioxidant capacity evaluation

**Fig. 5.**
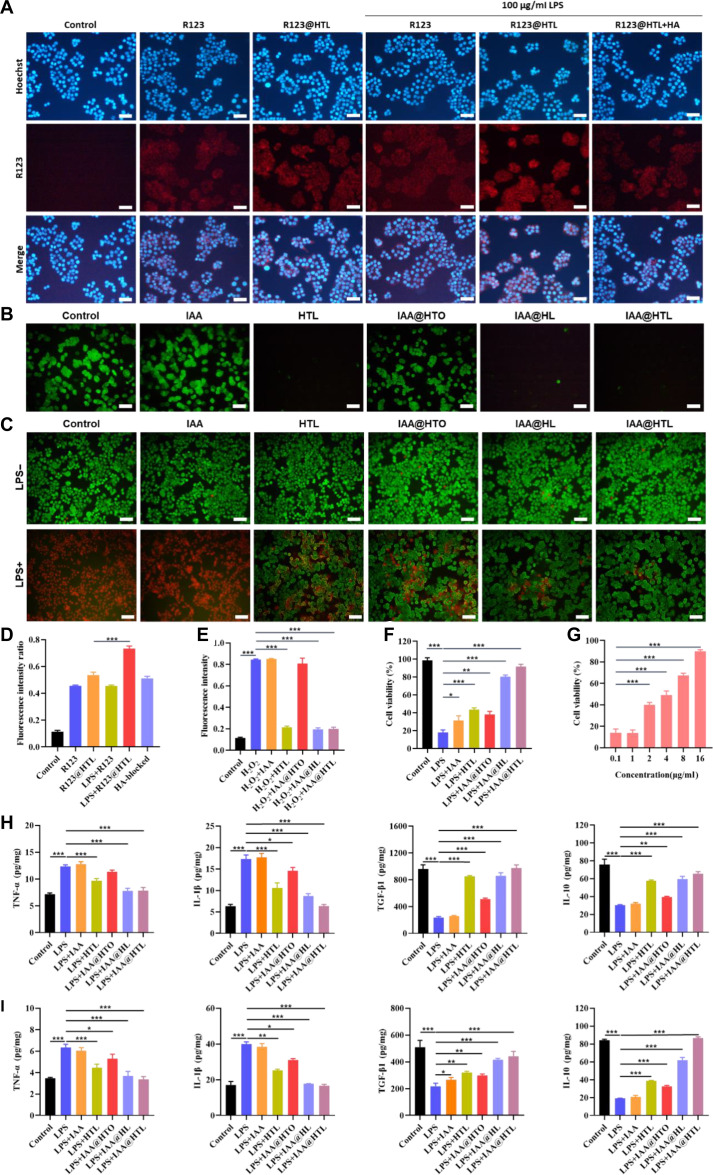
Investigation of nanoparticles’ cellular uptake, antioxidant protection, antiapoptotic, and anti-inflammatory effects. (A) Cellular uptake behavior of rhodamine 123 (R123)-labeled nanoparticles: Fluorescence microscopy images of HT-29 cells after coincubation with R123-loaded nanoparticles or free R123 solution (red: R123; blue: 4′,6-diamidino-2-phenylindole [DAPI] nuclear staining). Scale bar: 100 μm. (B) Reactive oxygen species (ROS) scavenging capacity of different nanoparticles: Changes in DCFH-DA fluorescence intensity (green: ROS; blue: DAPI) in H_2_O_2_ (200 μmol/l)-induced HT-29 cells treated with different nanoparticles. Scale bar: 100 μm. (C) Live/dead cell staining analysis: Calcein-acetoxymethyl ester (AM)/propidium iodide (PI) double staining showing the viability status of lipopolysaccharide (LPS)-treated HT-29 cells after intervention with different nanoparticles (green: live cells; red: dead cells). Scale bar: 100 μm. (D) Semiquantitative analysis results of HT-29 cellular uptake experiments. (E) Semiquantitative analysis of intracellular 2',7'-dichlorofluorescein (DCF) fluorescence intensity in HT-29 cells. (F and G) Protective effects of different nanoparticles on cell viability (MTT assay): (F) Effects of different nanoparticles on the survival rate of LPS-stimulated HT-29 cells. (G) Concentration gradient (0.1 to 16 μg/ml) of IAA@HTL nanoparticles (NPs) on alleviating LPS-induced cytotoxicity. (H and I) Enzyme-linked immunosorbent assay (ELISA) detection of inflammatory cytokine secretion levels: Concentration changes of proinflammatory factors (tumor necrosis factor-α [TNF-α] and interleukin-1β [IL-1β]) and anti-inflammatory factors (transforming growth factor-β1 [TGF-β1] and IL-10) in culture supernatants of RAW 264.7 cells (H) and HT-29 cells (I). Data are presented as means ± SD (*n* = 3). ^*^P < 0.05, ^**^P < 0.01, ^***^P < 0.001.

In the inflammatory microenvironment of UC, the expression of CD44 receptors is markedly up-regulated on the surfaces of macrophages and intestinal epithelial cells. As an important transmembrane glycoprotein, CD44 exhibits high affinity for HA in the extracellular matrix. Leveraging this active HA-targeting mechanism, the drug-loaded NPs in this study are designed to be actively internalized by epithelial cells and macrophages within the inflamed mucosal lamina propria, thereby enhancing the intracellular concentration of therapeutic agents. The hydrophobic fluorescent molecule R123 was used to replace IAA, forming R123@HTL NPs to achieve active targeted delivery via CD44–HA interaction. Uptake of free R123 and R123@HTL NPs by HT-29 and RAW 264.7 cells was observed using inverted fluorescence microscopy. Results showed weak fluorescence in normal cells for both groups, with slightly higher intensity in the R123@HTL group than in the free R123 group, indicating basal CD44-mediated internalization. After LPS induction, fluorescence in the R123@HTL group increased significantly, while the free R123 group showed no change, suggesting that LPS stimulation enhances CD44 expression and promotes NP uptake. In competitive binding assays, preincubation with HA markedly inhibited the internalization of R123@HTL, confirming that cellular uptake is mediated by specific CD44–HA binding (Fig. 5A and Fig. S16A). Semiquantitative analysis indicated stronger uptake of R123@HTL in LPS-induced macrophages, which may be attributed to their immune effector functions and metabolic characteristics (Fig. 5D and Fig. S17). These results demonstrate that IAA@HTL NPs can effectively target inflammatory cells through the HA–CD44 mechanism, enhancing drug accumulation and laying the foundation for subsequent studies on ROS scavenging and AhR activation.

In colonic tissues of UC patients, ROS levels are significantly elevated. Excessive ROS not only directly oxidizes biological macromolecules but also exacerbates inflammatory responses by activating the NLRP3 inflammasome and suppressing the Nrf2 pathway, among other mechanisms [33]. For intracellular ROS measurement, an oxidative stress model was established by stimulating HT-29 and RAW 264.7 cells with H₂O₂, and ROS levels were detected using the DCFH-DA fluorescent probe. As shown in Fig. 5B and Fig. S16B, H_2_O_2_ stimulation significantly enhanced fluorescence intensity. Intervention experiments revealed that neither free IAA nor IAA@HTO effectively reduced intracellular ROS levels, whereas all formulations containing LA (HTL, IAA@HL, and IAA@HTL) exhibited significant ROS scavenging capacity. Among them, IAA@HTL NPs restored fluorescence intensity to near-normal levels, indicating superior antioxidant efficacy (Fig. 5E and Fig. S18). These results confirm that LA efficiently scavenges ROS and helps reestablish intracellular redox homeostasis, thereby creating favorable conditions for IAA to activate AhR and exert anti-inflammatory and epithelial repair effects.

#### Assessment of apoptosis inhibition ability

During acute UC flares, inflammatory responses induce intestinal epithelial cell apoptosis through multiple mechanisms, primarily involving excessive release of proinflammatory cytokines and oxidative stress-induced aberrant activation of apoptotic signaling pathways. The increased apoptosis, coupled with an imbalance in compensatory proliferation, leads to severe impairment of the intestinal barrier function and accelerates disease progression. Studies have shown that activation of the AhR pathway can modulate inflammatory responses, enhance antioxidant defenses, and promote epithelial regeneration, thereby restoring epithelial homeostasis and facilitating tissue repair [34]. In this study, we first evaluated the protective effects of different IAA formulations in an LPS-induced HT-29 cell inflammation model by using the MTT assay. As shown in Fig. 5F and G, treatment with 1 μg/ml LPS had reduced cell viability to below 20%. Free IAA, blank HTL NPs, and IAA@HTO NPs provided limited improvement in cell viability (all below 40%). In contrast, IAA@HTL NPs and IAA@HL NPs, which contained LA, exhibited significant protective effects, with IAA@HTL NPs showing the highest efficacy, restoring cell viability to over 80%. In concentration-response experiments, the protective effect of IAA@HTL NPs was dose-dependent: As the IAA concentration increased from 4 to 16 μg/ml, cell viability progressively improved, reaching nearly 90% at the highest concentration, indicating a substantial protective effect against LPS-induced cellular injury within the tested range.

The protective effects of different formulations on epithelial cells were further assessed using live/dead cell double staining. As shown in Fig. 5C, strong green fluorescence was observed in the untreated control and all experimental groups, with no significant differences among groups. The control group exhibited the strongest red fluorescence, followed by the free IAA group. Compared to free IAA, the IAA@HTO group showed significantly enhanced green fluorescence, indicating that the nanocarrier facilitated IAA uptake through HA-mediated targeting, thereby enhancing AhR pathway-associated repair functions. Notably, the blank HTL NP, IAA@HL NP, and IAA@HTL NP groups all displayed more pronounced enhancement of green fluorescence, with the drug-loaded groups outperforming the blank carrier group. These results demonstrate that LA confers protection by counteracting oxidative stress, while the nanodelivery system efficiently delivers IAA, activates the AhR pathway, and alleviates inflammatory damage. To quantitatively evaluate the antiapoptotic effects of the NPs, Annexin V-FITC/PI double staining coupled with flow cytometry was performed. As shown in Figs. S19 and S20, LPS treatment significantly increased the proportions of both early and late apoptotic cells. Although free IAA exhibited some inhibitory effect on apoptosis, IAA@HTO NPs demonstrated a stronger antiapoptotic effect, suggesting that HA-mediated targeted delivery enhances intracellular IAA concentration and efficacy. Blank HTL NPs, IAA@HL NPs, and IAA@HTL NPs all exhibited more pronounced antiapoptotic effects, with the drug-loaded groups outperforming the blank carrier group. This confirms that IAA-mediated AhR activation, in synergy with LA-regulated redox homeostasis, effectively suppresses epithelial cell apoptosis and promotes functional recovery.

#### Evaluation of macrophage polarization and inflammatory cytokine expression regulation

Macrophages play crucial roles in maintaining intestinal immune homeostasis. M2 macrophages, characterized by their anti-inflammatory and tissue-repair functions, are key regulators of intestinal homeostasis. They help maintain a tolerogenic mucosal immune environment by phagocytosing apoptotic cells and pathogens and by secreting regulatory cytokines such as IL-10 and TGF-β. However, during the pathogenesis of UC, stimuli like LPS and interferon-γ can drive macrophage polarization toward the M1 phenotype. These M1 macrophages promote the excessive secretion of proinflammatory cytokines (e.g., TNF-α and IL-1β) and reactive oxygen/nitrogen species, thereby exacerbating inflammation and oxidative stress. Furthermore, M1 macrophages can amplify immune damage through antigen presentation and T cell activation. Consequently, modulating macrophage polarization has emerged as a key therapeutic strategy for alleviating intestinal inflammation and promoting mucosal repair [35]. In flow cytometry experiments, the polarization state of macrophages was assessed by detecting the expression of M1 (CD86) and M2 (CD206) surface markers. The results demonstrated that LPS successfully induced M1 polarization in RAW264.7 cells. Free IAA partially suppressed M1 polarization, while HTL (blank NPs) and IAA@HTO NPs showed moderate inhibitory effects. In contrast, IAA@HL NPs and IAA@HTL NPs exhibited more significant suppression of M1 polarization, with the IAA@HTL group showing the lowest percentage of CD86^+^ cells and the strongest inhibitory effect (Figs. S21A and S22). Regarding M2 polarization, using IL-4-treated cells as a positive control, all drug-loaded NP groups (IAA@HTO, IAA@HL, and IAA@HTL), as well as free IAA, significantly promoted M2 polarization. Notably, the NP groups were more effective than free IAA in this regard (Figs. S21B and S23). To further evaluate the immunomodulatory effects of the NPs, changes in the expression of key cytokines were measured. The results indicated that LPS treatment significantly up-regulated proinflammatory cytokines (TNF-α and IL-1β) and down-regulated anti-inflammatory cytokines (TGF-β1 and IL-10). Free IAA showed limited regulatory capacity, whereas IAA@HTO NPs, by enhancing cellular uptake of IAA, demonstrated improved immunomodulatory effects. Blank HTL NPs, owing to the antioxidant activity of LA, exhibited a certain degree of immunomodulatory capability, albeit weaker than that of IAA@HL and IAA@HTL NPs. IAA@HTL NPs exhibited the strongest immunomodulatory capacity (Fig. 5H). A consistent trend in cytokine modulation was observed in epithelial cells across the treatment groups (Fig. 5I). These findings collectively demonstrate that: (a) IAA can modulate macrophage polarization, and this regulatory effect is enhanced by the nanocarriers through improved cellular internalization; (b) blank HTL NPs inhibit M1 polarization and modulate inflammatory cytokine secretion via the antioxidant action of LA; (c) compared to the individual components, IAA@HTL NPs achieve the optimal polarization regulation and anti-inflammatory effects in both intestinal epithelial cells and macrophages, likely attributable to the synergistic interplay between the respective regulatory activities of IAA and LA.

Given that the above experimental results indicated that both IAA and LA played positive roles in regulating macrophage polarization and inflammatory factor secretion, we further investigated whether there was a synergistic anti-inflammatory effect when IAA and LA were used in combination. TNF-α was used as a representative proinflammatory cytokine, and the Chou–Talalay CI method was employed to evaluate their inhibitory effects on TNF-α in an LPS-stimulated RAW264.7 cell model. CI analysis showed that at effect levels of half-maximal effective dose (ED50), 75% effective dose (ED75), 90% effective dose (ED90), and 95% effective dose (ED95), the CI values were 0.472, 0.409, 0.356, and 0.325, respectively, all far below 0.9. Moreover, as the effect level increased, the CI values gradually decreased, indicating a concentration-dependent enhancement of strong synergistic effects between the 2 drugs in inhibiting TNF-α (Fig. S24). This synergistic effect may be attributed to LA creating a favorable redox microenvironment for IAA to activate the AhR pathway by scavenging ROS, thereby promoting its anti-inflammatory effects. At the same time, LA itself can directly inhibit the production of inflammatory factors by scavenging ROS. The 2 agents work together through different mechanisms to synergistically suppress the development of inflammation.

### In vivo targeting capability analysis

**Fig. 6.**
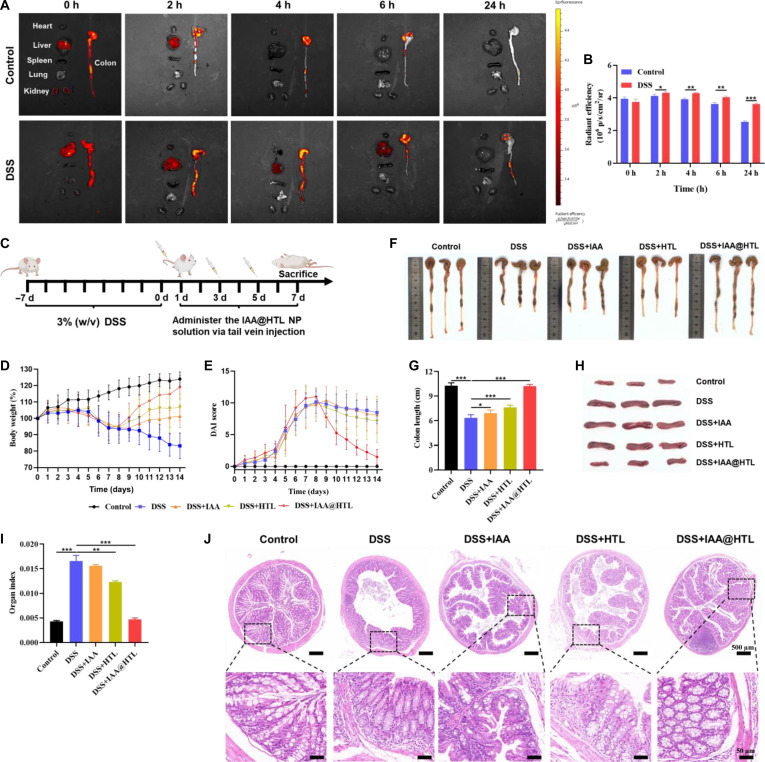
Evaluation of in vivo targeting and therapeutic effects of IAA@HTL nanoparticles (NPs). (A) Biodistribution characteristics of R123-labeled nanoparticles: Ex vivo fluorescence imaging (red: R123 signal) of major organs (heart, liver, spleen, lungs, and kidneys) and colon at different time points (0, 2, 4, 6, and 24 h) after intravenous injection of R123@HTL NPs in healthy mice and dextran sulfate sodium (DSS)-induced colitis model mice. (B) Quantitative targeting analysis: Temporal changes in R123 fluorescence intensity in colon tissues (*n* = 3). (C) Schematic diagram of the experimental design showing DSS-induced colitis model establishment and tail vein administration protocol. (D) Body weight changes during treatment and (E) dynamic curves of disease activity index (DAI) (*n* = 6). (F) Representative ex vivo colon photographs and (G) quantitative analysis of colon length across treatment groups (*n* = 6). (H) Ex vivo spleen photographs and (I) statistical analysis of spleen index (spleen weight/body weight) (*n* = 6). (J) Histopathological analysis of colon tissues by hematoxylin-eosin (H&E) staining (scale bars: upper panel, 500 μm; lower panel, 100 μm). Data are presented as means ± SD. ^*^P < 0.05, ^**^P < 0.01, ^***^P < 0.001.

A murine UC model was established by administering 3% DSS for 7 d. R123 was employed as a fluorescent tracer substitute for IAA to evaluate the biodistribution and targeting capability of IAA@HTL NPs in colitic tissue. Following intravenous injection, the results (Fig. 6A and B and Fig. S25) revealed that during the initial phase (0 to 2 h), the NPs rapidly distributed to major organs, including the liver, kidneys, and colon, in both healthy and DSS-treated mice. In healthy mice, the colonic fluorescence signal began to diminish rapidly after 2 h, primarily confined to the ileocecal region, and became nearly undetectable by 24 h. In contrast, DSS-induced colitic mice exhibited strong fluorescence signals throughout the entire colon from 0 to 4 h, which persisted up to 24 h, with the signals encompassing both the ileocecal and distal colon regions. Notably, during the early postinjection period (liver: 0 to 6 h; kidneys: 0 to 2 h), NP accumulation in the liver and kidneys was higher in DSS-treated mice compared to healthy controls. However, by 24 h, the fluorescence intensity in all major organs (including the liver and kidneys) was lower than that in the colonic tissue (Fig. S25). These findings indicate that intravenously administered IAA@HTL NPs, following systemic circulation, accumulate in the liver and kidneys via reticuloendothelial system capture and renal clearance mechanisms. Under inflammatory conditions, the NPs additionally extravasate into the intestinal lamina propria through the EPR effect across dilated vascular endothelium and are subsequently internalized by macrophages and epithelial cells via CD44 receptor-mediated active targeting, thereby exerting their therapeutic action. In contrast, normal colonic tissue, lacking the EPR effect and inflammation-associated pathological alterations, fails to facilitate long-term NP retention.

### Pharmacokinetic analysis

The HPLC analytical method exhibited good linearity within the concentration range of 0.1 to 50 μg/ml (*R*^2^ = 0.9993) (Fig. S26). The relative standard deviations for intraday and interday precision were both less than 3%, the spiked recovery rates ranged from 99% to 103%, and the samples remained stable within 24 h, meeting the requirements for biological sample analysis (Tables S6 to S8). The plasma concentration–time curves of free IAA and IAA@HTL following tail vein injection in rats are shown in Fig. S27, and the main pharmacokinetic parameters are listed in Table S9. Compared with the free IAA group, the peak concentration (Cmax) of the IAA@HTL group increased to 2.07 times that of the free IAA, the half-life (t1/2) was prolonged to 2.11 times, and the area under the curve (AUC_0~t_ and AUC_0~∞_) increased to 1.60 times and 1.25 times, respectively, while the clearance rate (CL) decreased by 52%. The mean residence time (MRT_0~t_ and MRT_0~∞_) was extended to 1.26 times and 1.30 times, respectively. These results indicated that IAA@HTL NPs greatly improved the pharmacokinetic behavior of IAA, prolonged the circulation time of the drug in vivo, enhanced its bioavailability, and demonstrated favorable sustained-release effects and potential for clinical application.

### Biocompatibility assessment

As shown in Fig. S28A and B, when calculated based on HA concentration, HTL, HTO, and HL exhibited no significant cytotoxicity against both HT-29 and RAW 264.7 cells within the concentration range of 0.1 to 50 μg/ml, with cell viability remaining above 80%. Further investigation (Fig. S28C and D) demonstrated that IAA-loaded NPs based on these polymers also showed excellent biocompatibility in the range of 0.1 to 16 μg/ml (based on IAA concentration), maintaining cell viability above 80% in both cell lines. The safety evaluation following intravenous administration (Figs. S29 and S30) revealed that IAA@HTL, IAA@HTO, and IAA@HL NPs all induced HRs below 2% at concentrations ranging from 2.5 to 16 μg/ml (based on IAA concentration). No notable hemolysis was observed compared to the Triton X-100 positive control group, indicating favorable hemocompatibility suitable for intravenous injection. To further assess the long-term biosafety of the NPs, we administered mice daily tail vein injections at a dose of 80 μg/kg for 14 consecutive days, with a PBS group serving as the negative control. Throughout the administration period, no adverse effects such as constipation, vomiting, or diarrhea were observed in any experimental group. Upon completion of the experiment, major organs (heart, liver, spleen, lungs, kidneys, and colon) and peripheral blood samples were collected for toxicological analysis. Histopathological examination (Fig. S31) demonstrated that all NP-treated groups maintained intact tissue architecture in major organs compared to the control group, with no apparent pathological alterations observed.

Hematological parameters including red blood cell count (RBC), hemoglobin (HGB), hematocrit (HCT), mean corpuscular volume (MCV), mean corpuscular hemoglobin (MCH), mean corpuscular hemoglobin concentration (MCHC), red cell distribution width (RDW), white blood cell count (WBC), absolute neutrophil count (Gran), absolute lymphocyte count (Lym), platelet count (PLT), mean platelet volume (MPV), platelet distribution width (PDW), and plateletcrit (PCT) were analyzed. As shown in Fig. S32, no significant differences were detected between experimental and control groups. Hepatic function markers including total bilirubin (T-BIL), direct bilirubin (D-BIL), albumin (ALB), alanine aminotransferase (ALT), aspartate aminotransferase (AST), alkaline phosphatase (ALP), γ-glutamyl transferase (GGT), and total bile acid (TBA), along with renal function parameters including blood urea nitrogen (BUN) and creatinine (CREA), were evaluated. As presented in Fig. S33, no statistically significant differences were observed between experimental and control groups.

### Therapeutic efficacy assessment and mechanism analysis in experimental colitis

The therapeutic efficacy of IAA@HTL NPs was evaluated in a murine UC model induced by DSS (Fig. 6C). As shown in Fig. 6D and Fig. S34, all DSS-treated mice exhibited progressive weight loss starting from day 4 of modeling, reaching the nadir on day 8 (calculated from the treatment initiation day). Following treatment, body weight recovered to varying degrees across all experimental groups, with the IAA@HTL group demonstrating the most significant recovery by day 15, superior to all other groups. The DAI, which comprehensively assesses weight loss, stool consistency, and fecal bleeding, was used to evaluate colitis severity [36]. At the end point of the treatment period (day 15), compared to the DSS model group, the IAA, HTL, and IAA@HTL groups exhibited reductions in DAI scores of 5%, 16%, and 83%, respectively (Fig. 6E), indicating that IAA@HTL NPs provided the most substantial alleviation of colitis severity. Macroscopic examination of the colon revealed pronounced shortening, edema, and hemorrhage in the DSS group. In contrast, IAA@HTL treatment resulted in a 34%, 48%, and 61% increase in colon length compared to the DSS, IAA, and HTL groups, respectively (Fig. 6F and G). Spleen weight and the spleen index serve as indicators of systemic inflammation. As shown in Fig. 6H and I, the DSS group exhibited a marked increase in both spleen weight and the spleen index, indicative of immune activation and systemic inflammation. IAA@HTL treatment most effectively reduced these parameters, confirming its potent anti-inflammatory effect. Histopathological analysis by H&E staining (Fig. 6J) demonstrated an intact epithelial barrier and well-defined crypt structures in the normal control group. The DSS group displayed characteristic pathological features, including epithelial-barrier atrophy, crypt destruction, and extensive inflammatory cell infiltration. The IAA@HTL treatment group exhibited the best restorative outcomes, characterized by a largely intact epithelial barrier, clear crypt architecture, and near-complete resolution of inflammatory infiltration. Furthermore, examination of major organs revealed no pathological alterations in any treatment group, confirming the favorable safety profile of the administered formulations (Fig. S35).

**Fig. 7.**
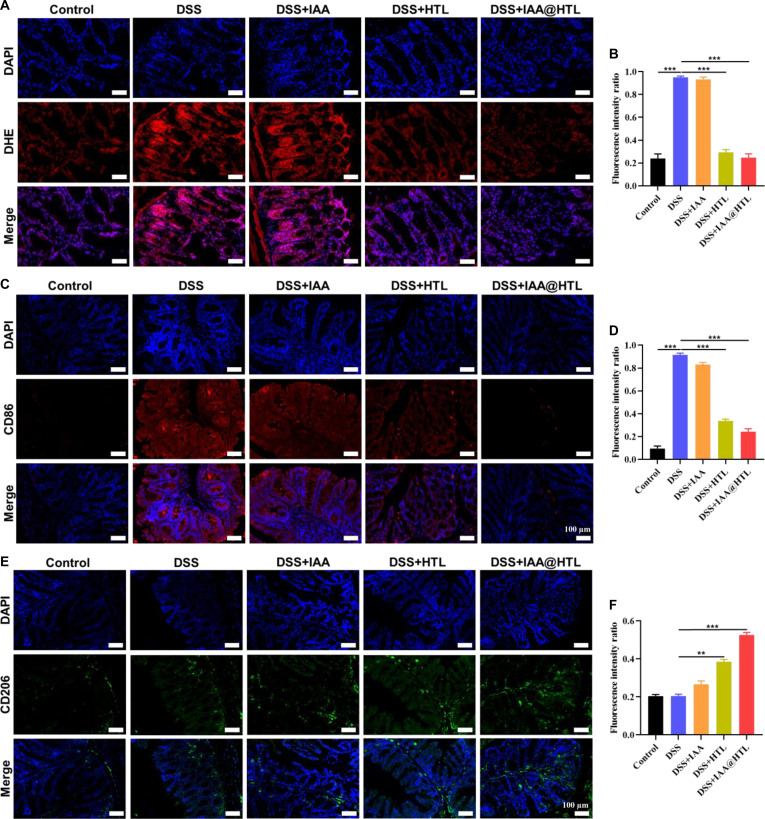
Qualitative and quantitative characterization of reactive oxygen species (ROS) levels and macrophage polarization status in colonic tissues. (A and B) Dihydroethidium (DHE) staining for detecting ROS levels in colon tissues (A) and semiquantitative analysis of DHE staining in mouse colon tissues based on the DHE staining images (B). (C and E) Immunofluorescence analysis of macrophage polarization phenotypes (C: CD86^+^ M1-type, E: CD206^+^ M2-type). (D and F) Semiquantitative analysis of macrophage M1 (D) and M2 (F) polarization in mouse colon tissues based on immunofluorescence images. Data are presented as means ± SD (*n* = 6). ^**^P < 0.01, ^***^P < 0.001.

To validate the in vivo ROS scavenging capacity of the NPs, the levels of ·O_2_^−^ in colonic tissues were detected using DHE fluorescence staining. As the primary ROS generated by the mitochondrial electron transport chain, ·O_2_^−^ can be converted to H_2_O_2_ and subsequently give rise to secondary ROS species. Its accumulation level directly reflects the degree of tissue oxidative stress [37]. As shown in Fig. 7A and B, compared to the normal group, the DSS model group exhibited a significant increase in DHE fluorescence intensity, indicating substantial ·O_2_^−^ accumulation. The ·O_2_^−^ level in the free IAA group was comparable to that in the model group. In contrast, both the LA-containing HTL and IAA@HTL groups effectively reduced ·O_2_^−^ levels, with the IAA@HTL group showing fluorescence intensity nearly equivalent to the normal control group, demonstrating the most potent antioxidant effect. Regarding macrophage polarization, the expression of the M1 marker CD86 (Fig. 7C and D) and the M2 marker CD206 (Fig. 7E and F) was assessed via immunofluorescence staining. Quantitative analysis revealed that the CD86 fluorescence intensity in the DSS group was significantly higher than in the normal control group, indicating a predominance of M1 proinflammatory polarization under inflammatory conditions. The inhibitory effect on CD86 expression increased progressively across the treatment groups in the order of IAA, HTL, and IAA@HTL. Concurrently, the expression of the anti-inflammatory marker CD206 showed a stepwise increase, with the most pronounced effect observed in the IAA@HTL group.

These results collectively demonstrate that free IAA, due to insufficient targeting capability and low cellular uptake efficiency, fails to effectively modulate intracellular oxidative stress and macrophage polarization status. In contrast, IAA@HTL NPs, through active targeting to specific cells, substantially enhance the intracellular concentrations of both IAA and LA. This leads to highly efficient regulation of oxidative stress and macrophage polarization. Within macrophages, the antioxidant activity of LA acts in synergy with the pharmacological activity of IAA, promoting a more substantial shift toward the anti-inflammatory (M2) phenotype, thereby culminating in optimal therapeutic efficacy.

**Fig. 8.**
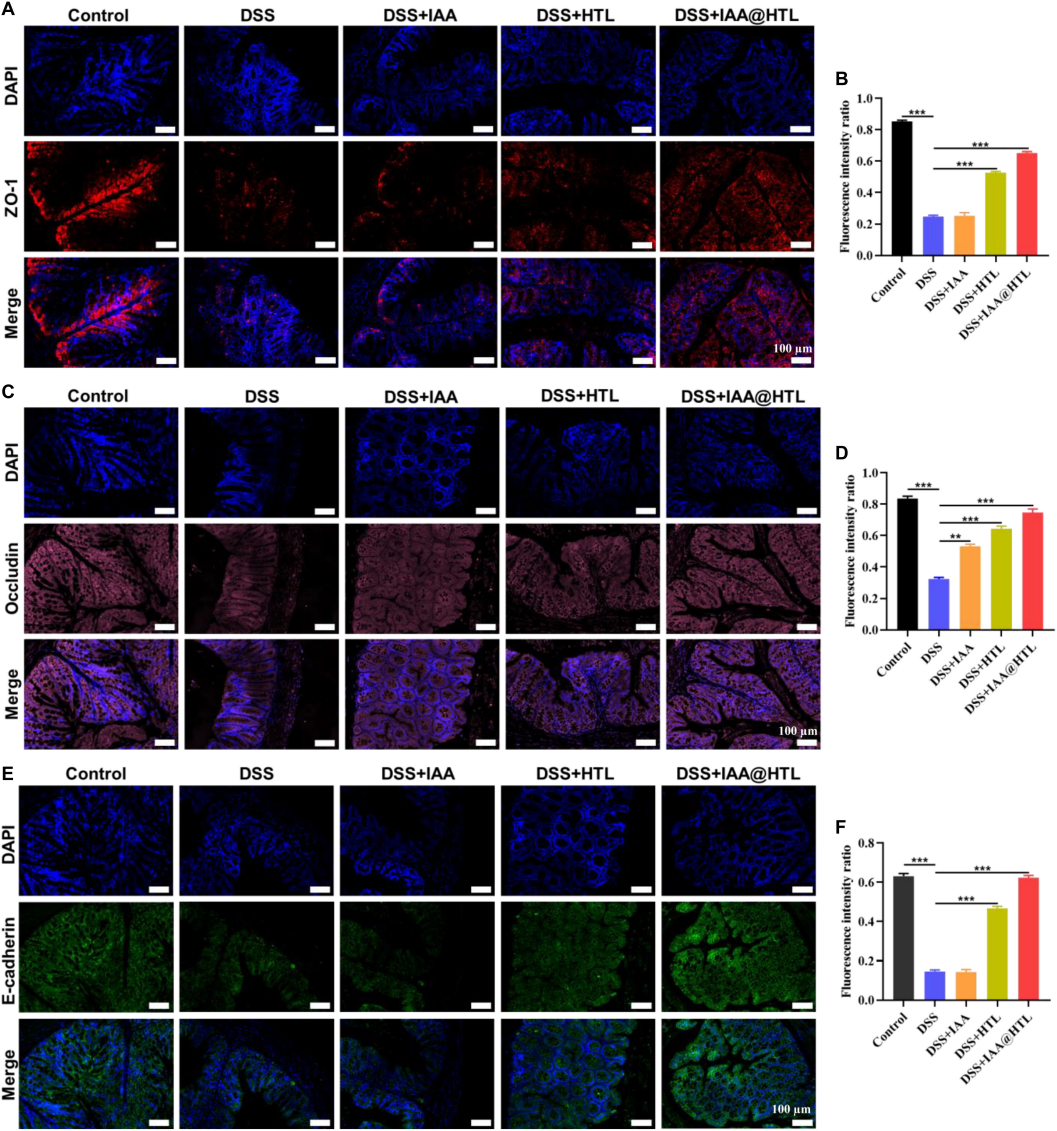
Effects of IAA@HTL nanoparticles (NPs) on the expression of intestinal epithelial junction-related proteins. (A, C, and E) Immunofluorescence images of intestinal barrier-related proteins in different treatment groups, A: ZO-1, C: Occludin, E: E-cadherin. Scale bar: 100 μm. (B, D, and F) Semiquantitative analysis of the relative expression levels of ZO-1 (B), Occludin (D), and E-cadherin (F) from fluorescence images of colon tissues in mice from different treatment groups. Data are presented as means ± SD (*n* = 6). ^**^P < 0.01, ^***^P < 0.001.

The intestinal epithelium serves as a critical physical and functional barrier between the internal milieu and the external environment. Its structural integrity is maintained by intercellular junctions, primarily TJs and AJs. These junctional proteins not only constitute a mechanical barrier against pathogens and toxins but are also involved in maintaining cell polarity, signal transduction, and immune regulation [38]. Impaired expression or function of these proteins increases intestinal permeability, facilitating the translocation of harmful substances and thereby initiating or exacerbating colitis. This study systematically evaluated the regulatory effects of IAA@HTL NPs on key junctional proteins—ZO-1, Occludin, and E-cadherin—under inflammatory conditions. ZO-1 acts as a scaffold protein within TJs, mediating the connection between transmembrane proteins and the cytoskeleton, and coordinating barrier integrity and the dynamic assembly of TJs [39]. Occludin is a crucial transmembrane protein of TJs, directly contributing to the intercellular seal and regulating paracellular permeability and epithelial polarity [40]. E-cadherin, the core adhesion molecule of AJs, mediates calcium-dependent cell–cell adhesion and is essential for maintaining epithelial homeostasis and suppressing tumor invasion [41]. Immunofluorescence images (Fig. 8A, C, and E) and their semiquantitative analysis (Fig. 8B, D, and F) revealed a significant reduction in the fluorescence intensity of ZO-1, Occludin, and E-cadherin in the DSS model group compared to the control group, indicating severe disruption of the junctional protein architecture by the inflammatory response. All treatment groups (IAA, HTL, and IAA@HTL) demonstrated a restorative trend in protein expression. Notably, the IAA@HTL group exhibited fluorescence intensity closest to the normal level, representing the most significant recovery effect. These findings demonstrate that the active targeting capability of IAA@HTL to inflamed epithelium effectively enhances the intracellular delivery of both IAA and LA. The concerted actions of LA-mediated ROS scavenging and IAA-driven AhR pathway activation synergistically inhibit epithelial cell death and up-regulate junctional protein expression, culminating in the restoration of epithelial-barrier integrity at both structural and functional levels.

**Fig. 9.**
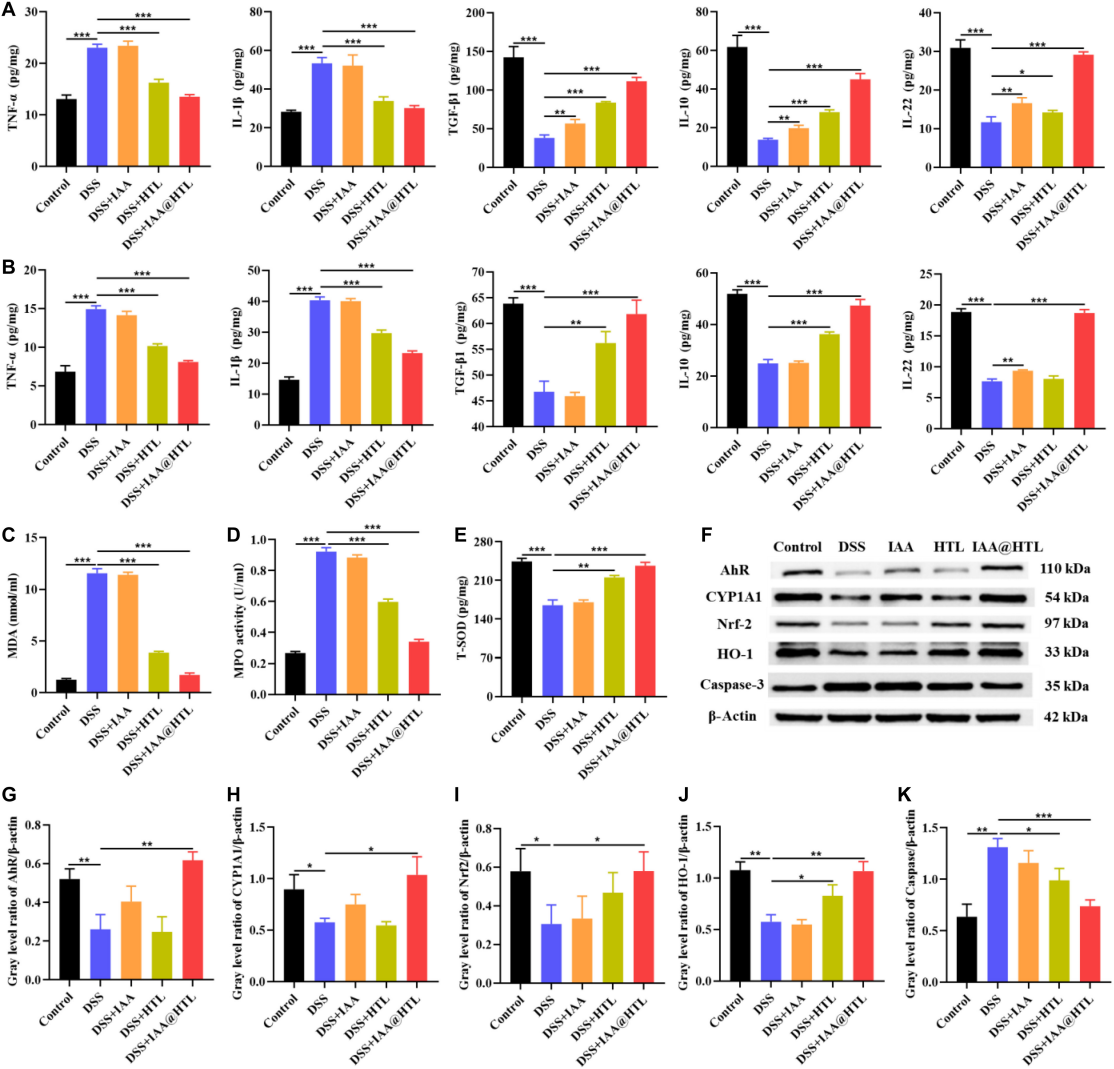
Effects of IAA@HTL nanoparticles (NPs) on inflammatory factor secretion, main oxidative stress markers, and critical regulatory protein expression in the intestinal mucosa. (A and B) Enzyme-linked immunosorbent assay (ELISA) detection of inflammatory cytokines (tumor necrosis factor-α [TNF-α], interleukin-1β [IL-1β], transforming growth factor-β1 [TGF-β1], IL-10, and IL-22) in colon tissues (A) and serum (B) (*n* = 6). Quantitative analysis of malondialdehyde (MDA) (C), myeloperoxidase (MPO) (D), and superoxide dismutase (SOD) (E) levels in colon tissues of mice from different treatment groups (*n* = 6). ^**^P < 0.01, ^***^P < 0.001. (F) Western blot analysis of aryl hydrocarbon receptor (AhR)/CYP1A1/Nrf-2/HO-1/cleaved Caspase-3 protein expression. (G to K) Western blot analysis of protein expression levels in colon tissues of mice from different treatment groups: (G) AhR, (H) CYP1A1, (I) Nrf2, (J) HO-1, and (K) Caspase 3 (normalized to β-actin as loading control, with relative expression calculated based on band density) (*n* = 3). ^*^P < 0.05, ^**^P < 0.01, ^***^P < 0.001.

During the progression of UC, the inflammatory response acts as both a core event disrupting mucosal homeostasis and a key trigger for excessive ROS generation. As shown in Fig. 9A and B, compared to the control group, the levels of proinflammatory cytokines (TNF-α and IL-1β) were significantly elevated in both the colonic tissue and serum of DSS-induced model mice, whereas the levels of anti-inflammatory cytokines (TGF-β1 and IL-10) were markedly reduced. Intervention with IAA@HTL NPs demonstrated the most potent anti-inflammatory efficacy: It not only effectively suppressed the expression of TNF-α and IL-1β but also substantially elevated the levels of TGF-β1 and IL-10. This effect was superior to those observed in the free IAA and HTL groups, indicating that IAA@HTL can simultaneously ameliorate both local and systemic inflammatory states. IL-22, a key effector cytokine of the AhR pathway, is primarily secreted by type 3 innate lymphoid cells and natural killer cells. It exerts mucosal protective effects through multiple mechanisms, including promoting epithelial proliferation, enhancing mucus secretion, and inducing antimicrobial peptide production [9]. Results in Fig. 9A and B showed that IAA@HTL restored IL-22 levels more effectively than free IAA, while the HTL group exhibited a weaker effect. These findings suggest that IAA@HTL not only effectively suppresses inflammation but also promotes IL-22 secretion, thereby providing crucial support for mucosal barrier repair. Measurements of key intestinal oxidative stress markers are presented in Fig. 9C (MPO), Fig. 9D (MDA), and Fig. 9E (SOD). The DSS model group exhibited a significant increase in colonic MDA content and MPO activity, alongside a marked decrease in SOD activity, indicating severe oxidative stress [42]. Both HTL and IAA@HTL treatments effectively reduced MDA and MPO levels and enhanced SOD activity, with the IAA@HTL group showing the most pronounced effects. These results indicate that the synergistic interaction between IAA and LA within IAA@HTL provides superior control over oxidative stress, thereby establishing a favorable microenvironment for subsequent anti-inflammatory responses and mucosal repair.

Western blot analysis revealed that, compared to the control group, the DSS model group exhibited significant down-regulation of AhR, CYP1A1, Nrf-2, and HO-1 protein expression, alongside a marked up-regulation of Caspase-3, indicating activation of the apoptotic pathway under inflammatory conditions. In the intervention studies, IAA treatment significantly up-regulated AhR and its downstream target CYP1A1 but had a limited effect on activating Nrf-2 and HO-1 and failed to effectively inhibit Caspase-3 expression. HTL, while not significantly affecting AhR/CYP1A1 expression, notably promoted the Nrf-2/HO-1 pathway and effectively suppressed Caspase-3. In contrast, the combined IAA@HTL intervention exhibited a synergistic effect: It most potently activated the AhR/CYP1A1 signaling, significantly up-regulated Nrf-2/HO-1 expression, and maximally inhibited Caspase-3 (Fig. 9F to K). These results indicate that IAA@HTL NPs establish a multipathway interactive regulatory network through the synergistic action of their components, collectively mitigating UC. Specifically, IAA acts as a key ligand, effectively activating the AhR/CYP1A1 pathway, while HTL, via efficient ROS scavenging, drives the up-regulation of the Nrf-2/HO-1 pathway. Notably, extensive crosstalk exists between the AhR and Nrf-2 pathways, creating a “bidirectional regulation, functional synergy” effect. That is, AhR activation can induce Nrf-2/HO-1, and Nrf-2 can, in turn, feedback-regulate AhR activity and CYP1A1 function, thereby achieving functional complementarity in anti-inflammatory and antioxidant responses [43]. Building upon this, IAA@HTL, by concurrently activating both pathways, inhibits proinflammatory signals like NF-κB and reduces ROS accumulation, thereby curbing M1 macrophage polarization. Concurrently, it promotes M2 macrophage polarization by activating the peroxisome proliferator–activated receptor γ/signal transducer and activator of transcription 6 pathway and enhancing overall antioxidant defense [44,45]. This dual regulatory action effectively reshapes the intestinal immune microenvironment and alleviates inflammation. Furthermore, the activated AhR/CYP1A1 pathway can also up-regulate antiapoptotic proteins (e.g., Bcl-2) and inhibit the mitochondrial apoptotic pathway, thereby reducing Caspase-3 activation [46,47], providing direct antiapoptotic protection for intestinal epithelial cells.

### Intestinal microbiota regulation analysis

**Fig. 10.**
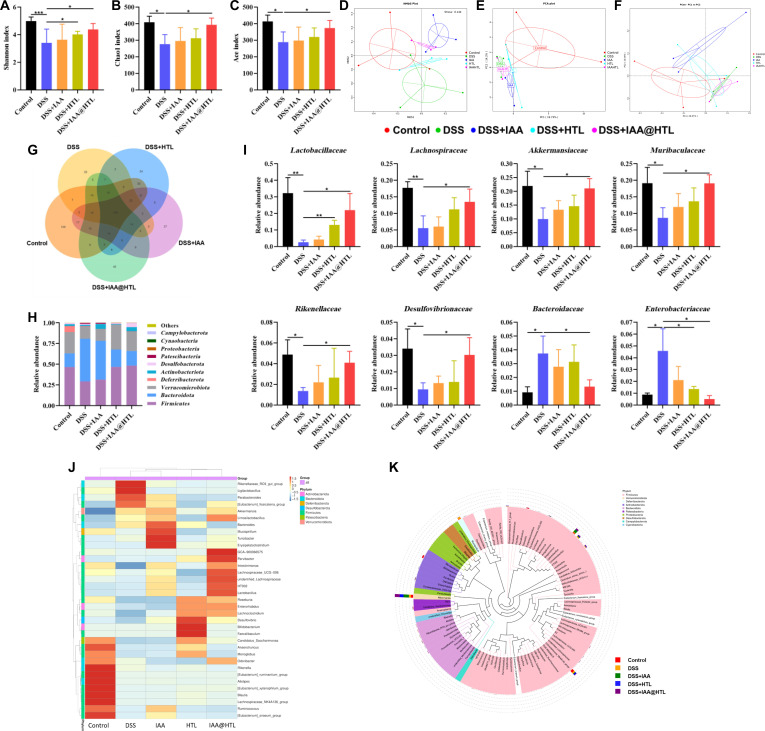
Regulatory effects of IAA@HTL on gut microbiota. (A to C) α-Diversity analysis: (A) Shannon index. (B) Chao1 index. (C) Ace index. (D to F) β-diversity analysis: (D) Multidimensional scaling (MDS) plot. (E) Principal component analysis (PCA) plot. (F) Principal coordinates analysis (PCoA) plot. (G) Venn diagram of microbial community composition differences. (H) Phylum-level relative abundance bar chart. (I) Family-level relative abundance bar chart. (J) Genus-level heatmap. (K) Genus-level phylogenetic tree. Data are presented as means ± SD (*n* = 5). ^*^P < 0.05, ^**^P < 0.01, ^***^P < 0.001.

Current evidence indicates that gut microbiota dysbiosis contributes to the pathogenesis of UC by disrupting immune tolerance. An imbalance between commensal and pathogenic bacteria can trigger aberrant activation of the intestinal mucosal immune system, ultimately leading to chronic inflammation [3]. To evaluate the regulatory effect of IAA@HTL NPs on gut microbiota homeostasis, this study employed 16S ribosomal RNA gene high-throughput sequencing to analyze the composition and species richness of the fecal microbiota. To ensure data reliability, sample sufficiency was first verified by species accumulation boxplots, while rarefaction curves and rank-abundance curves confirmed that species richness, evenness, and sequencing depth met the required standards, validating the accuracy and reliability of the sequencing results (Fig. S36). Compared to the blank control group, the DSS group exhibited a significant reduction in α-diversity indices (including Shannon, Chao1, and Ace indices). Intervention with IAA@HTL effectively restored microbial richness and diversity (Fig. 10A to C). Beta diversity, assessed by principal component analysis, principal coordinates analysis, and nonmetric multidimensional scaling (Fig. 10D to F), revealed significant separation among the microbial communities of the 5 groups. The IAA@HTL group clustered closer to the blank control group than the DSS group. Venn diagram analysis identified 260 operational taxonomic units common to all groups. The number of unique operational taxonomic units was 100 for the blank control group, 25 for the DSS group, 37 for the IAA group, 34 for the HTL group, and 45 for the IAA@HTL group, indicating that DSS treatment reduced species richness, which was restored by IAA@HTL therapy (Fig. 10G). To further investigate the impact of different treatments on gut microbiota composition, changes in microbial communities at various taxonomic levels were analyzed. At the phylum level (Fig. 10H), all groups contained *Firmicutes*, *Bacteroidetes*, *Verrucomicrobiota*, *Deferribacterota*, *Actinobacteria*, *Desulfobacterota*, and *Proteobacteria*. Compared to the control group, DSS-treated mice showed a decrease in *Firmicutes* and an increase in *Bacteroidetes*—a dysbiosis pattern that was significantly reversed by IAA@HTL treatment. Notably, IAA@HTL also reduced the relative abundance of the pathogenic phylum *Proteobacteria* compared to the DSS group. At the family level (Fig. 10I), control mice maintained high abundances of beneficial bacteria, including *Lactobacillaceae*, *Lachnospiraceae*, *Akkermansiaceae*, *Muribaculaceae*, *Rikenellaceae*, and *Desulfovibrionaceae*. DSS treatment significantly reduced these beneficial families while increasing the abundance of harmful *Bacteroidaceae* and *Enterobacteriaceae*. Although free IAA or HTL NPs showed limited effects, the combined IAA@HTL therapy maximally restored beneficial bacterial families and suppressed pathogenic ones compared to the DSS control. Genus-level analysis via abundance heatmap and phylogenetic tree (Fig. 10J and K) demonstrated that the microbiota structure of IAA@HTL-treated mice highly resembled that of the healthy control group. Ternary plot analysis (Fig. S37) further confirmed that the microbial profile of the IAA@HTL group was significantly different from the DSS model and closely matched the control group, indicating its capacity to correct DSS-induced dysbiosis. Mechanistic studies suggest that IAA can directly modulate the microbiota via the AhR pathway: Activating AhR in epithelial cells promotes the secretion of TJ proteins, restoring intestinal mucosal structure; activating AhR in type 3 innate lymphoid cells promotes IL-22 secretion, which in turn induces the expression of antimicrobial peptides (e.g., Reg3γ and β-defensins), inhibiting the growth of pathogenic bacteria [48,49]; concurrently, activating AhR signaling in macrophages induces the production of the anti-inflammatory cytokine IL-10, alleviating intestinal inflammation and promoting the colonization of beneficial bacteria [50]. Leveraging their ability to actively target epithelial cells and macrophages, IAA@HTL NPs further enhance the intracellular concentrations of IAA and LA, thereby amplifying the regulatory effects. Ultimately, this system achieves a “one-stone-three-birds” therapeutic outcome by activating the AhR pathway and modulating oxidative stress, thereby suppressing inflammatory progression, restoring the mucosal barrier, and reestablishing gut microbiota homeostasis.

## Conclusion

In this study, we engineered a redox-responsive nanodrug delivery system (IAA@HTL) for the targeted delivery of the natural AhR ligand, IAA. This system utilizes HA as the hydrophilic backbone, which is grafted with LA via a TK linker, enabling efficient IAA encapsulation and targeted delivery. The resulting NPs exhibited favorable physicochemical properties, high encapsulation efficiency, and drug loading capacity, coupled with prominent ROS/GSH dual-responsive drug release behavior. In vitro experiments confirmed that IAA@HTL NPs could be efficiently internalized by inflamed intestinal epithelial cells and macrophages via CD44 receptor-mediated active targeting. Subsequently, they effectively scavenged intracellular ROS, inhibited apoptosis, promoted macrophage polarization toward the M2 anti-inflammatory phenotype, and modulated inflammatory cytokine expression. In a DSS-induced murine UC model, IAA@HTL NPs specifically accumulated in the inflamed colonic tissue through the EPR effect and active targeting mechanisms. This led to marked alleviation of disease symptoms, restoration of colon length, and improvement of histopathological damage. Mechanistic investigations revealed that IAA@HTL synergistically activated both the AhR/CYP1A1 and Nrf2/HO-1 signaling pathways. This dual activation not only suppressed inflammatory responses, strengthened the epithelial-barrier function, and promoted IL-22 secretion but also effectively modulated the gut microbiota structure, restoring the diversity of the gut microbiota and the abundance of beneficial bacteria. In summary, this study developed a novel nanotherapeutic strategy capable of simultaneously achieving “inflammation alleviation–mucosal repair–microbiota restoration”, representing a “one-stone-three-birds” approach. It provides new insights and experimental evidence for the synergistic treatment of UC. Looking toward clinical translation, the IAA@HTL NPs present several distinguishing features from the numerous NP systems previously explored for inflammatory bowel disease. Unlike conventional single-mechanistic approaches, our system uniquely integrates a therapeutic synergy: The LA-mediated ROS scavenging actively modulates the inflammatory microenvironment to potentiate the AhR activation by IAA. This cooperative action achieves a comprehensive “one-stone-three-birds” outcome, which is rarely accomplished by single-agent therapies. From a translational standpoint, the use of naturally derived and biocompatible components (HA and LA) combined with a simple, scalable nanoprecipitation method offers substantial advantages in terms of manufacturing feasibility and biosafety. The demonstrated in vivo safety profile, coupled with the active targeting via CD44, positions this formulation for reduced off-target toxicity and enhanced therapeutic precision. By addressing multiple pathological facets of UC with a single, well-tolerated nanomedicine, IAA@HTL NPs represent a promising candidate worthy of further development in large animal models and eventual clinical evaluation for patients suffering from UC.

## Data Availability

The datasets used and/or analyzed during the current study are available from the corresponding author on reasonable request.

## References

[1] Le Berre C, Honap S, Peyrin-Biroulet L. Ulcerative colitis. Lancet. 2023;402(10401):571–584. 37573077 10.1016/S0140-6736(23)00966-2

[2] Tang X, Huang Y, Zhu Y, Xu Y. Immune dysregulation in ulcerative colitis: Pathogenic mechanisms and therapeutic strategies of traditional Chinese medicine. Front Cell Dev Biol. 2025;13:1610435. 40538978 10.3389/fcell.2025.1610435 PMC12176777

[3] Bu F, Chen K, Chen SC, Jiang Y. Gut microbiota and intestinal immunity interaction in ulcerative colitis and its application in treatment. Front Cell Infect Microbiol. 2025;15:1565082. 40292216 10.3389/fcimb.2025.1565082 PMC12031664

[4] Li H, Pan M, Li Y, Cui M, Zhang M. New targets for the treatment of ulcerative colitis: Gut microbiota and its metabolites. Comput Struct Biotechnol J. 2025;27:1850–1863. 40470314 10.1016/j.csbj.2025.05.006 PMC12136715

[5] Barroso A, Mahler JV, Fonseca-Castro PH, Quintana FJ. The aryl hydrocarbon receptor and the gut-brain axis. Cell Mol Immunol. 2021;18(2):259–268. 33408340 10.1038/s41423-020-00585-5 PMC8027889

[6] Wang G, Fan Y, Zhang G, Cai S, Ma Y, Yang LJ, Wang Y, Yu H, Qiao S, Zeng X. Microbiota-derived indoles alleviate intestinal inflammation and modulate microbiome by microbial cross-feeding. Microbiome. 2024;12(1):59. 38504383 10.1186/s40168-024-01750-y PMC10949743

[7] Stockinger B, Shah K, Wincent E. AHR in the intestinal microenvironment: Safeguarding barrier function. Nat Rev Gastroenterol Hepatol. 2021;18(8):559–570. 33742166 10.1038/s41575-021-00430-8 PMC7611426

[8] Quintana FJ, Basso AS, Iglesias AH, Korn T, Farez MF, Bettelli E, Caccamo M, Oukka M, Weiner HL. Control of T_reg_ and T_H_17 cell differentiation by the aryl hydrocarbon receptor. Nature. 2008;453(7191):65–71. 18362915 10.1038/nature06880

[9] Lee JS, Cella M, McDonald KG, Garlanda C, Kennedy GD, Nukaya M, Mantovani A, Kopan R, Bradfield CA, Newberry RD, et al. AHR drives the development of gut ILC22 cells and postnatal lymphoid tissues via pathways dependent on and independent of Notch. Nat Immunol. 2011;13(2):144–151. 22101730 10.1038/ni.2187 PMC3468413

[10] Wang MY, Li JM, Wu YL, Zhang Y, Hu T, Su WJ, Feng JF, Jiang CL. Activation of aryl hydrocarbon receptor (AhR) alleviates depressive-like behaviors in LPS-induced mice. Sheng Li Xue Bao. 2024;76(3):353–364. 38939930

[11] Vogel CFA, Khan EM, Leung PSC, Gershwin ME, Chang WLW, Wu DL, Haarmann-Stemmann T, Hoffmann A, Denison MS. Cross-talk between aryl hydrocarbon receptor and the inflammatory response: A role for nuclear factor-κB. J Biol Chem. 2014;289(3):1866–1875. 24302727 10.1074/jbc.M113.505578 PMC3894361

[12] Ashique S, Mishra N, Garg A, Sibuh BZ, Taneja P, Rai G, Djearamane S, Wong LS, Al-Dayan N, Roychoudhury S, et al. Recent updates on correlation between reactive oxygen species and synbiotics for effective management of ulcerative colitis. Front Nutr. 2023;10:1126579. 37545572 10.3389/fnut.2023.1126579 PMC10400011

[13] Li X, Fang P, Mai J, Choi ET, Wang H, Yang X-f. Targeting mitochondrial reactive oxygen species as novel therapy for inflammatory diseases and cancers. J Hematol Oncol. 2013;6:19. 23442817 10.1186/1756-8722-6-19 PMC3599349

[14] Yang C, Du Y, Li Q, Liu L, Zhao L, Gao C, Tang Z, Zhang X, Zhao Y, Yang X. Fructo-oligosaccharides alleviated ulcerative colitis via gut microbiota-dependent tryptophan metabolism in association with aromatic hydrocarbon receptor activation in mice. J Agric Food Chem. 2024;72(50):27912–27922. 39641614 10.1021/acs.jafc.4c07248

[15] Jing W, Dong S, Xu Y, Liu J, Ren J, Liu X, Zhu M, Zhang M, Shi H, Li N, et al. Gut microbiota-derived tryptophan metabolites regulated by Wuji Wan to attenuate colitis through AhR signaling activation. Acta Pharm Sin B. 2025;15(1):205–223. 40041900 10.1016/j.apsb.2024.11.009 PMC11873645

[16] Rannug A. How the AHR became important in intestinal homeostasis-a diurnal FICZ/AHR/CYP1A1 feedback controls both immunity and immunopathology. Int J Mol Sci. 2020;21(16):5681. 32784381 10.3390/ijms21165681 PMC7461111

[17] Packer L, Witt EH, Tritschler HJ. Alpha-lipoic acid as a biological antioxidant. Free Radic Biol Med. 1995;19(2):227–250. 7649494 10.1016/0891-5849(95)00017-r

[18] Liu G, Li K, Wang H. Polymeric micelles based on PEGylated chitosan-g-lipoic acid as carrier for efficient intracellular drug delivery. J Biomater Appl. 2017;31(7):1039–1048. 28178903 10.1177/0885328216685755

[19] Liu L, Liu P. Synthesis strategies for disulfide bond-containing polymer-based drug delivery system for reduction-responsive controlled release. Front Mater Sci. 2015;9(3):211–226.

[20] Zhou X, Wu Y, Zhu Z, Lu C, Zhang C, Zeng L, Xie F, Zhang L, Zhou F. Mucosal immune response in biology, disease prevention and treatment. Signal Transduct Target Ther. 2025;10(1):7. 39774607 10.1038/s41392-024-02043-4 PMC11707400

[21] Dong K, Zhang Y, Ji HR, Guan ZL, Wang DY, Guo ZY, Deng SJ, He BY, Xing JF, You CY. Dexamethasone-loaded lipid calcium phosphate nanoparticles treat experimental colitis by regulating macrophage polarization in inflammatory sites. Int J Nanomedicine. 2024;19:993–1016. 38299194 10.2147/IJN.S442369 PMC10829593

[22] de Paula MC, Carvalho SG, Silvestre ALP, dos Santos AM, Meneguin AB, Chorilli M. The role of hyaluronic acid in the design and functionalization of nanoparticles for the treatment of colorectal cancer. Carbohydr Polym. 2023;320:121257. 37659830 10.1016/j.carbpol.2023.121257

[23] Wang H, Lin F, Wu Y, Guo W, Chen X, Xiao CS, Chen M. Carrier-free nanodrug based on co-assembly of methylprednisolone dimer and rutin for combined treatment of spinal cord injury. ACS Nano. 2023;17(13):12176–12187. 37387550 10.1021/acsnano.3c00360

[24] Ray L, Pal MK, Ray RS. Synergism of co-delivered nanosized antioxidants displayed enhanced anticancer efficacy in human colon cancer cell lines. Bioact Mater. 2017;2(2):82–95. 29744415 10.1016/j.bioactmat.2017.02.003 PMC5935044

[25] Li CJ, Wang N, Zheng GD, Yang LC. Oral administration of resveratrol-selenium-peptide nanocomposites alleviates Alzheimer’s disease-like pathogenesis by inhibiting Aβ aggregation and regulating gut microbiota. ACS Appl Mater Interfaces. 2021;13(39):46406–46420. 34569225 10.1021/acsami.1c14818

[26] Superti F, Russo R. Alpha-lipoic acid: Biological mechanisms and health benefits. Antioxidants. 2024;13(10):1228. 39456481 10.3390/antiox13101228 PMC11505271

[27] Ke C, Sun L, Qiao D, Wang D, Zeng X. Antioxidant acitivity of low molecular weight hyaluronic acid. Food Chem Toxicol. 2011;49(10):2670–2675. 21787831 10.1016/j.fct.2011.07.020

[28] Patergnani S, Bouhamida E, Leo S, Pinton P, Rimessi A. Mitochondrial oxidative stress and “Mito-inflammation”: Actors in the diseases. Biomedicine. 2021;9(2):216. 10.3390/biomedicines9020216 PMC7923430 33672477

[29] Guo Z, He H, Liu K, Li Z, Yang S, Liao Z, Lai C, Ren X, Huang B, Pan X. The photolytic behavior of COVID-19 antivirals ribavirin in natural waters and the increased environmental risk. J Hazard Mater. 2023;452:131320. 37002997 10.1016/j.jhazmat.2023.131320 PMC10043975

[30] Govender T, Stolnik S, Garnett MC, Illum L, Davis SS. PLGA nanoparticles prepared by nanoprecipitation: Drug loading and release studies of a water soluble drug. J Control Release. 1999;57(2):171–185. 9971898 10.1016/s0168-3659(98)00116-3

[31] Kim W, Ly NK, He Y, Li Y, Yuan Z, Yeo Y. Protein corona: Friend or foe? Co-opting serum proteins for nanoparticle delivery. Adv Drug Deliv Rev. 2023;192:114635. 36503885 10.1016/j.addr.2022.114635 PMC9812987

[32] Wang Z, Li S, Cao Y, Tian X, Zeng R, Liao D-F, Cao D. Oxidative stress and carbonyl lesions in ulcerative colitis and associated colorectal cancer. Oxidative Med Cell Longev. 2016;2016:9875298. 10.1155/2016/9875298 PMC4707327 26823956

[33] Cao J, Bao Q, Hao H. Indole-3-carboxaldehyde alleviates LPS-induced intestinal inflammation by inhibiting ROS production and NLRP3 inflammasome activation. Antioxidants. 2024;13(9):1107. 39334766 10.3390/antiox13091107 PMC11429283

[34] Chen Y, Wang Y, Fu Y, Yin Y, Xu K. Modulating AHR function offers exciting therapeutic potential in gut immunity and inflammation. Cell Biosci. 2023;13(1):85. 37179416 10.1186/s13578-023-01046-y PMC10182712

[35] Zhang M, Li X, Zhang Q, Yang J, Liu G. Roles of macrophages on ulcerative colitis and colitis-associated colorectal cancer. Front Immunol. 2023;14:1103617. 37006260 10.3389/fimmu.2023.1103617 PMC10062481

[36] Dong K, Deng S-J, He B-Y, Guo Z-Y, Guan Z-L, Leng X, Ma R-R, Wang D-Y, Xing J-F, You C-Y. Mucoadhesive nanoparticles enhance the therapeutic effect of dexamethasone on experimental ulcerative colitis by the local administration as an enema. Drug Des Devel Ther. 2023;17:191–207. 10.2147/DDDT.S390274 PMC9884054 36718245

[37] Juan CA, Pérez de la Lastra JM, Plou FJ, Pérez-Lebeña E. The chemistry of reactive oxygen species (ROS) revisited: Outlining their role in biological macromolecules (DNA, lipids and proteins) and induced pathologies. Int J Mol Sci. 2021;22(9):4642. 33924958 10.3390/ijms22094642 PMC8125527

[38] Ramirez-Velez I, Belardi B. Storming the gate: New approaches for targeting the dynamic tight junction for improved drug delivery. Adv Drug Deliv Rev. 2023;199:114905. 37271282 10.1016/j.addr.2023.114905 PMC10999255

[39] Zhang Z, Xie Y, Yi Q, Liu J, Yang L, Wang R, Cai J, Li X, Feng X, Yao S, et al. PEAK1 maintains tight junctions in intestinal epithelial cells and resists colitis by inhibiting autophagy-mediated ZO-1 degradation. Nat Commun. 2025;16(1):6777. 40707483 10.1038/s41467-025-62107-z PMC12290104

[40] Furuse M, Hirase T, Itoh M, Nagafuchi A, Yonemura S, Tsukita S, Tsukita S. Occludin: A novel integral membrane protein localizing at tight junctions. J Cell Biol. 1993;123(6):1777–1788. 8276896 10.1083/jcb.123.6.1777 PMC2290891

[41] Niessen C, Tunggal JA, Helfrich I, Schwarz H, Günzel D, Fromm M, Kemler R, Krieg T. E-cadherin is essential for *in vivo* epidermal barrier function by regulating tight junctions. J Invest Dermatol. 2005;124(4):A32. 10.1038/sj.emboj.7600605 PMC556407 15775979

[42] Ma J, Wu D, Xu C, He Q, Wang M, Imran M, Nazar M, Li K. *Lactobacillus salivarius* alleviated dextran sulfate sodium (DSS)-induced colitis in mice by mitigating oxidative stress and inflammatory responses through modulation of the intestinal flora. Microb Pathog. 2025;205:107696. 40355053 10.1016/j.micpath.2025.107696

[43] Edamitsu T, Taguchi K, Okuyama R, Yamamoto M. AHR and NRF2 in skin homeostasis and atopic dermatitis. Antioxidants. 2022;11(2). 10.3390/antiox11020227 PMC8868544 35204110

[44] Yin Z, Song R, Yu T, Fu Y, Ding Y, Nie H. Natural compounds regulate macrophage polarization and alleviate inflammation against ALI/ARDS. Biomolecules. 2025;15(2):192. 40001495 10.3390/biom15020192 PMC11853067

[45] Zhang L, Li T, Liu J, Sun J, Niu J, Ren D, Ma Y, He Y, Liu S, Wang Q. The regulation of the NF-κB p65 and Nrf2/HO-1 signaling pathways by fucoxanthin in human THP-1 monocyte macrophages under a lipopolysaccharide-induced inflammation model. Foods. 2025;14(10):1746. 40428524 10.3390/foods14101746 PMC12110976

[46] Yi T, Wang J, Zhu K, Tang Y, Huang S, Shui X, Ding Y, Chen C, Lei W. Aryl hydrocarbon receptor: A new player of pathogenesis and therapy in cardiovascular diseases. Biomed Res Int. 2018;2018(1):6058784. 29984241 10.1155/2018/6058784 PMC6015699

[47] Rzemieniec J, Wnuk A, Lason W, Bilecki W, Kajta M. The neuroprotective action of 3,3-diindolylmethane against ischemia involves an inhibition of apoptosis and autophagy that depends on HDAC and AhR/CYP1A1 but not ERα/CYP19A1 signaling. Apoptosis. 2019;24(5–6):435–452. 30778709 10.1007/s10495-019-01522-2 PMC6522467

[48] Zhao XL, Xu LY, Li KD, Tang F, Liu D, Zhang JN, Cao ZJ, Peng C, Ao H. Exploring dried ginger essential oil as a therapeutic strategy for 5-FU-in-duced mucositis: Gut microbiota and tryptophan metabolite IAA-AHR/ IL-22/STAT3 signaling axis. J Ethnopharmacol. 2025;345:119616. 40074099 10.1016/j.jep.2025.119616

[49] Zhang Y, Han L, Dong J, Yuan Z, Yao W, Ji P, Hua Y, Wei Y. Shaoyao decoction improves damp-heat colitis by activating the AHR/ IL-22/STAT3 pathway through tryptophan metabolism driven by gut microbiota. J Ethnopharmacol. 2024;326:117874. 38342152 10.1016/j.jep.2024.117874

[50] Zhu J, Luo L, Tian L, Yin S, Ma X, Cheng S, Tang W, Yu J, Ma W, Zhou X, et al. Aryl hydrocarbon receptor promotes IL-10 expression in inflammatory macrophages through Src-STAT3 signaling pathway. Front Immunol. 2018;9:2033. 30283437 10.3389/fimmu.2018.02033 PMC6156150

