# Supplementary material for: A “One-Stone-Three-Birds” Inspired Nanoplatform for Multitargeted Ulcerative Colitis Therapy via Combined Aryl Hydrocarbon Receptor Activation and Reactive Oxygen Species Scavenging
Source: Biomater Res. 2026 May 12;30:0356. doi: 10.34133/bmr.0356 (PMC13161534; doi:10.34133/bmr.0356)
Supplement: Supplementary 1 — Supplementary Text Figs. S1 to S37 Tables S1 to S9 [file bmr.0356.f1.docx]

*Supporting information for*

**A “One-Stone-Three-Birds” Inspired Nanoplatform for Multi-Targeted Ulcerative Colitis Therapy via Combined AhR Activation and ROS Scavenging**

Kai Dong^1,2^, Zelin Guan^2^, Danyang Wang^2^, Jinyao Sun^1^, Ying Zhang^1^,

Cuiyu You^1^, Yuanming Xing^3*^

^1^Department of Pharmacy, The First Affiliated Hospital of Xi’an Jiaotong University, Xi’an, Shaanxi, China

^2^School of Pharmacy, Xi’an Jiaotong University, Xi’an, Shaanxi, China

^3^Department of Cardiovascular Medicine, The First Affiliated Hospital of Xi'an Jiaotong University, Xi'an, Shaanxi, China

***1. Experimental Methods***

***1.1 Synthesis of HA-TK-OA and HA-LA***

The synthesis of HA-TK-OA (HTO) was accomplished through the following steps: First, oleic acid (OA, 35 μL, 0.1 mmol) was dissolved in 2 mL of dichloromethane (DCM). Under nitrogen (N_2_) protection, EDC·HCl (0.2 mmol, 20 mg) dissolved in 1 mL of DCM was added sequentially and stirred for 30 min for activation, followed by the addition of NHS (0.2 mmol, 23 mg) dissolved in 3 mL of DCM for another 30 min of activation. Subsequently, TK-NH_2_ (0.15 mmol, 30 mg) dissolved in 1 mL of methanol was added, and the reaction was stirred continuously at room temperature under a N_2_ atmosphere for 48 h. After completion, 20 mL of cold diethyl ether was added, and the mixture was left to stand at 4 °C for 12 h to precipitate impurities. The solid collected on the filter paper was washed with 5 mL of deionized water for 10 min, and the aqueous phase was freeze-dried to obtain the OA-TK intermediate. In the second step of the synthesis, hyaluronic acid (HA, 7.5 mg) was swelled and dissolved in 1 mL of deionized water, then diluted with 9 mL of N, N-dimethylformamide (DMF). Under N_2_ protection, EDC·HCl (0.04 mmol, 4 mg) dissolved in 1 mL of DMF was added for 30 min of activation, followed by NHS (0.04 mmol, 4.6 mg) dissolved in 1 mL of DMF for another 30 min of activation. Finally, the OA-TK conjugate dissolved in 1 mL of DMF was added, and the reaction proceeded for 24 h. The product was dialyzed for 24 h using a dialysis bag with a 3,500 Da molecular weight cutoff to remove small-molecule impurities, and the pure HTO product was obtained by freeze-drying.

The synthesis of HA-LA (HL) was accomplished through the following steps: First, 20 mg of lipoic acid (LA) was completely dissolved in 8 mL of anhydrous DMF. Under N_2_ protection, a solution of EDC·HCl (37 mg) and NHS (23 mg) dissolved in 1 mL of DCM was sequentially added to the mixture. The reaction system was magnetically stirred at room temperature for 2 h to fully activate the terminal carboxyl group (-COOH) of the LA. Subsequently, a pre-prepared solution of 20 mg hyaluronic acid (HA) dissolved in 30 mL of DCM was slowly added dropwise to the activated system. The entire coupling reaction was strictly carried out under light-proof and nitrogen-protected conditions, with continuous stirring at room temperature for 24 h to ensure complete reaction. After the reaction was terminated, the resulting product solution was transferred into a dialysis bag with a molecular weight cutoff of 3,500 Da. The solution was dialyzed against ultrapure water as the dialysis medium at 4 °C for 48 h, with the dialysis solution replaced every 6 h to thoroughly remove unreacted raw materials and small-molecule byproducts. Finally, the dialyzed and purified solution was pre-frozen at -80 °C and then transferred to a freeze-dryer for lyophilization over 48 h, ultimately yielding a white fluffy solid of the HA-LA conjugate.

***1.2 Determination of Critical Micelle Concentration***

The critical micelle concentration (CMC) is a key parameter for evaluating the self-assembling ability of amphiphilic polymers, determining the minimum concentration required for the formation of stable nanostructures in aqueous solutions. Pyrene (Py), a typical hydrophobic polycyclic aromatic hydrocarbon molecule, exhibits unique fluorescence properties. In aqueous solutions, the fluorescence of Py is significantly quenched due to polar interactions with water molecules, whereas when encapsulated within the hydrophobic core of micelles, its fluorescence intensity markedly increases as it avoids the aqueous environment. This characteristic makes Py an ideal fluorescent probe for CMC determination. In this study, the CMC values of synthesized polymers (HTL, HTO, and HL) were measured using the pyrene fluorescence probe method. The specific steps were as follows: First, a Py stock solution (12.2 mg/L) was prepared in acetone, and 250 μL was added to volumetric flasks. After acetone evaporation, a series of HTL solutions with concentration gradients ranging from 0.0001 to 0.1 mg/mL was prepared using ultrapure water. The samples were shielded from light and equilibrated overnight at room temperature. The fluorescence spectrum of the 0.1 mg/mL sample was first measured to determine the excitation wavelengths (λ₁ and λ₂) corresponding to the two maximum emission peaks. Subsequently, the fluorescence intensities (I₁ and I₂) at λ₁ and λ₂ were measured for all concentration samples, and the I₁/I₂ ratio was calculated. A plot of the I₁/I₂ ratio (y-axis) against polymer concentration (x-axis) was generated, and the CMC value was identified as the concentration at the inflection point of the curve. The same method was applied to determine the CMC values of the control compounds HTO and HL.

***1.3 Development of in vitro IAA detection methods***

This study established an *in vitro* analytical method for Indole-3-acetic acid (IAA) through the following experiments: (1) Determination of detection wavelength: IAA standard was accurately weighed, dissolved in methanol, and diluted to 50 µg/mL to prepare the standard solution. The absorption spectrum curve was recorded by scanning the standard solution at 200-600 nm using a UV-visible spectrophotometer to identify the maximum absorption peak. (2) HPLC conditions and specificity investigation: The IAA concentration was determined by HPLC under the conditions listed in Table S1. The standard solution, IAA@HTL emulsion, and their mixture were injected and analyzed to record the retention times and evaluate separation efficiency. (3) Standard curve establishment: Precisely measured volumes (0.05, 0.1, 0.5, 1, 2, 5, and 10 mL) of the IAA standard solution were diluted to 10 mL with methanol to prepare seven concentration gradients (0.1-50 μg/mL). Each concentration was measured in triplicate, and the linear regression equation and correlation coefficient were derived by plotting the average peak area against concentration. (4) Precision test: Low (0.1 μg/mL), medium (5 μg/mL), and high (50 μg/mL) concentrations of IAA solutions were analyzed. Intraday precision was assessed by five consecutive injections in one day, while interday precision was evaluated by once-daily measurements over five consecutive days. The relative standard deviation (RSD) of peak areas was calculated. (5) Stability test: The IAA standard solution was stored at room temperature and analyzed at 0, 1, 2, 4, 8, 12, and 24 h to determine the RSD of peak areas at each time point. (6) Recovery test: Baseline samples of IAA solutions at three concentrations (5, 10, and 20 μg/mL) were prepared, and standard solutions equivalent to 80%, 100%, and 120% of the baseline concentration were added. Each concentration was tested in triplicate, and the recovery rate was calculated as Recovery (%) = (C_1_ - C_0_) × 100% / C_a_, where C_1_ was the measured concentration after spiking, C_0_ was the actual concentration before spiking, and C_a_ was the added standard concentration.


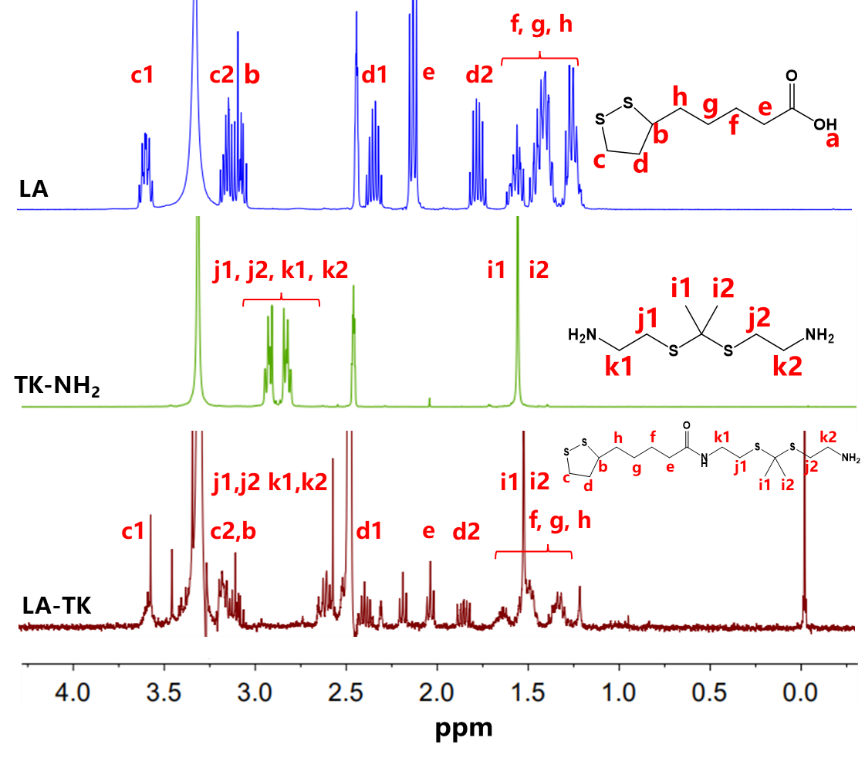


**Fig. S1** Expanded ^1^H-NMR spectra (0-4 ppm) of LA, TK-NH_2_, and LA-TK, highlighting characteristic peaks.


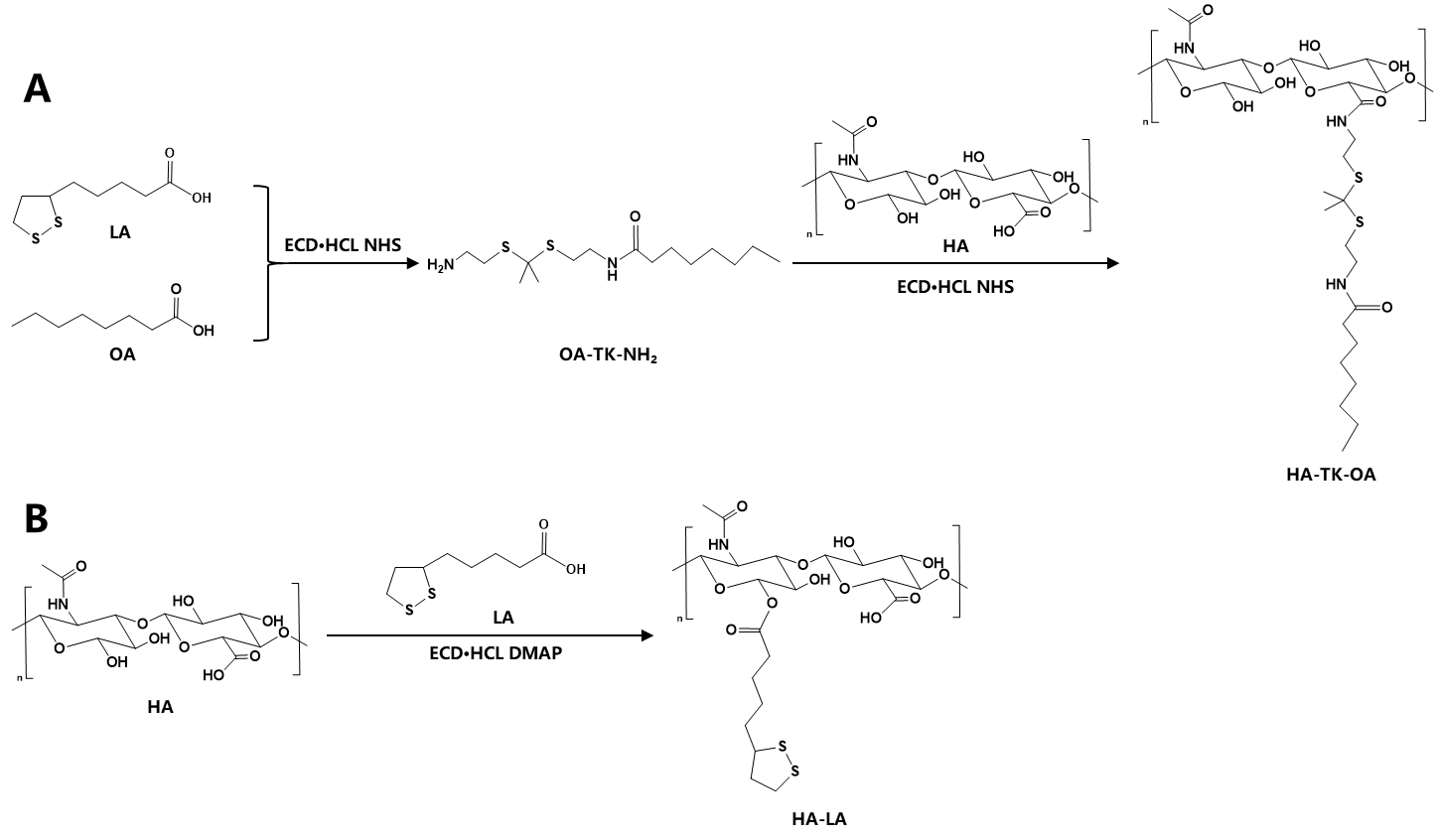


**Fig. S2** Schematic illustration of the synthetic routes for HA-TK-OA (HTO, **A**) and HA-LA (HL, **B**).


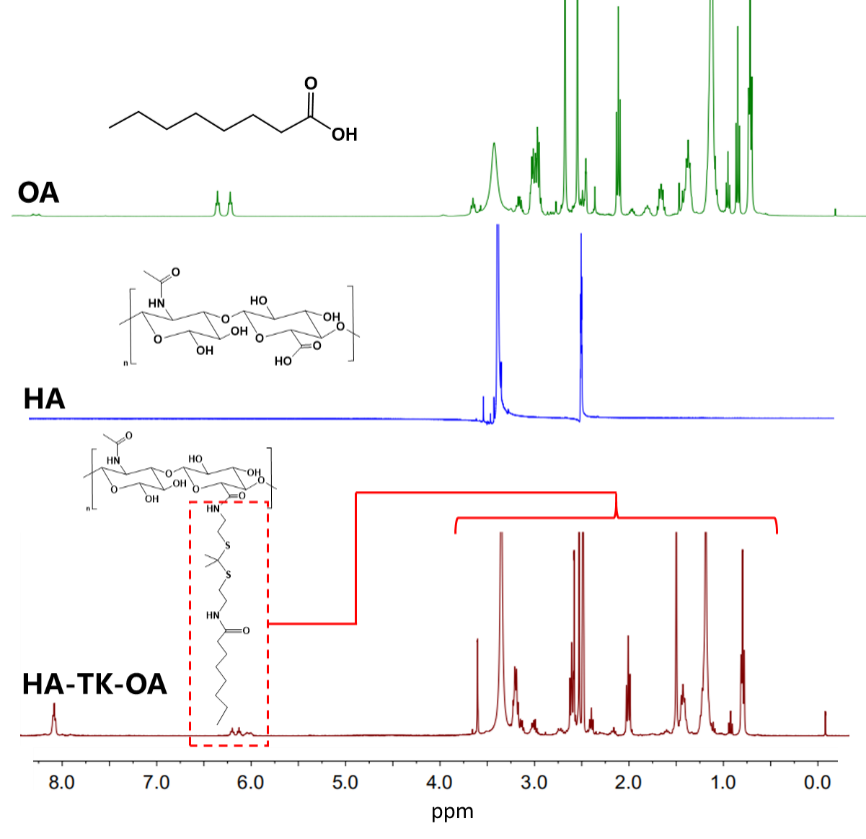


**Fig. S3** Partial expansion (0-8 ppm) of the ^1^H-NMR spectra of oleic acid (OA), hyaluronic acid (HA), and HTO, highlighting characteristic peaks.

As shown in **Fig. S3,** the ^1^H-NMR spectral analysis of HTO revealed that the characteristic peaks appearing at 3-3.5 ppm are attributed to the structural units of HA, while the proton signals in the 0-6 ppm range match the expected chemical shifts of the methylene groups (-CH_2_-) in OA. The presence of these characteristic peaks and their corresponding assignments confirmed the successful synthesis of HTO.


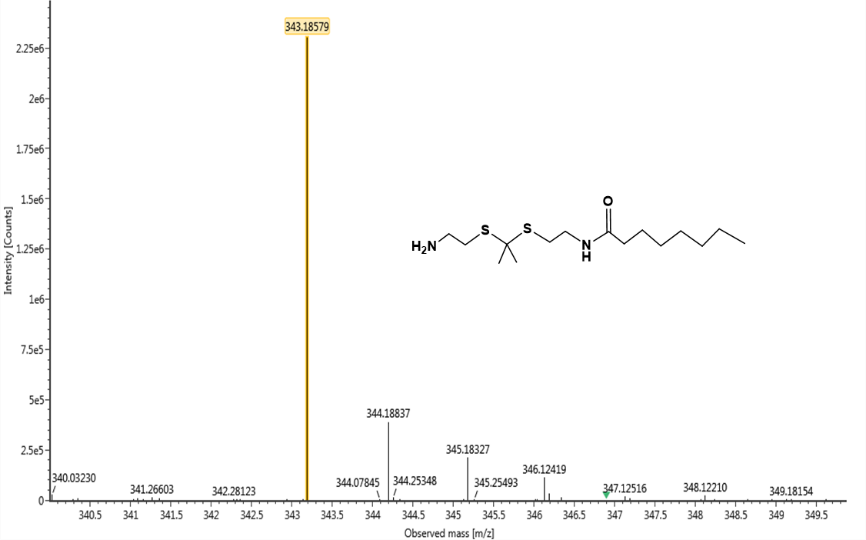


**Fig. S4** Electrospray ionization mass spectrometry (ESI-MS) of the OA-TK intermediate.

As shown in **Fig. S4**, the mass spectrometric analysis of OA-TK (molecular formula: C_15_H_30_N_2_OS_4_, theoretical molecular weight: 320.19561) revealed a quasi-molecular ion peak at m/z 343.18579 ([M+Na]^+^) in the ESI-MS spectrum, which matches the theoretically calculated value, confirming the successful synthesis of the OA-TK conjugate.


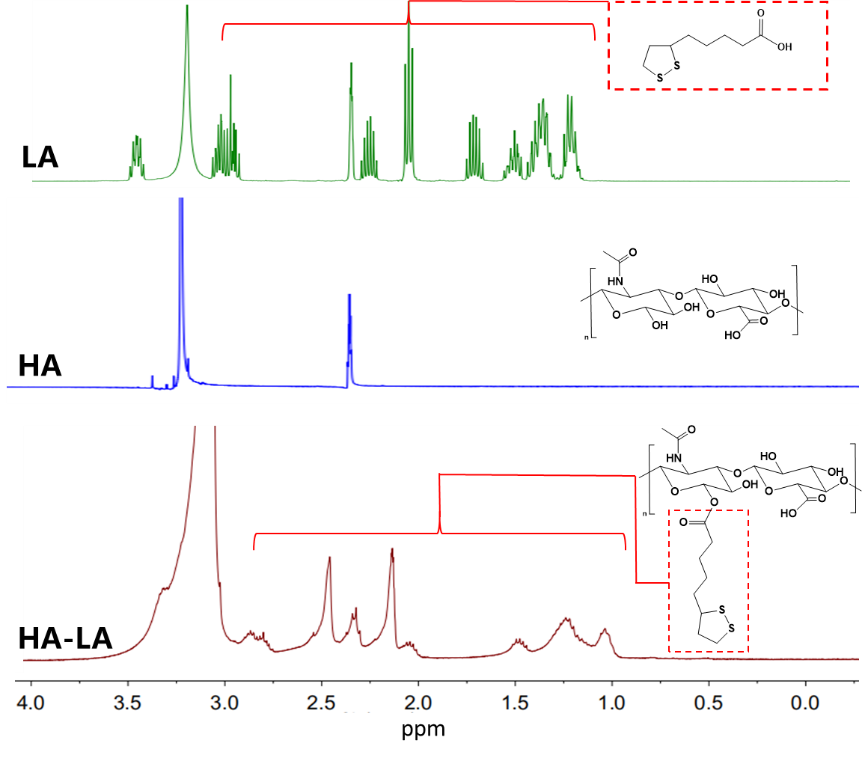


**Fig. S5** Partial expansion (0-4 ppm) of the ^1^H-NMR spectra of α-lipoic acid (LA), hyaluronic acid (HA), and HA-LA conjugate (HL), highlighting characteristic proton signals.

As shown in **Fig. S5**, the ^1^H-NMR spectrum of the HA-LA conjugate (HL) displays characteristic peaks at 3-3.5 ppm attributed to the protons of HA, while the methylene (-CH_2_-) and methine (-CH-) proton signals observed in the 0-3 ppm range are consistent with the ^1^H-NMR spectral features of LA, confirming the successful synthesis of HL.


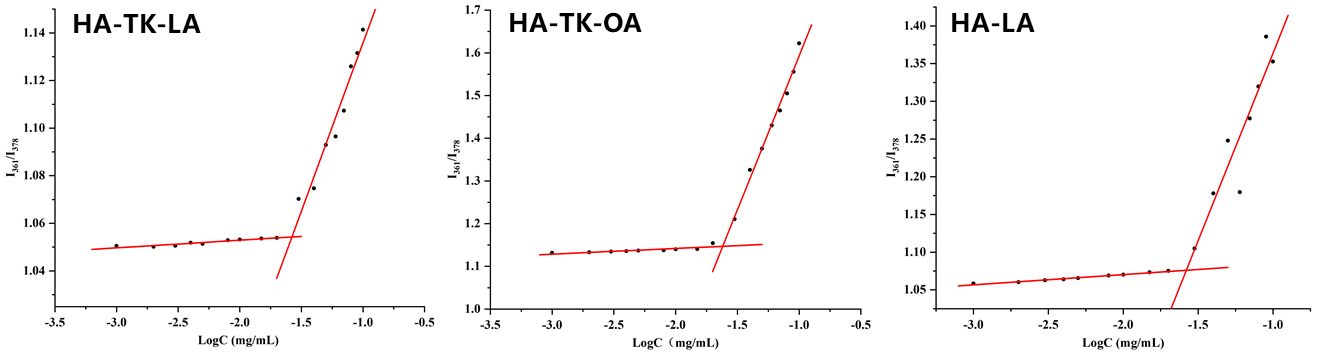


**Fig. S6** Determination of the critical micelle concentrations (CMC) for different graft copolymers.

**
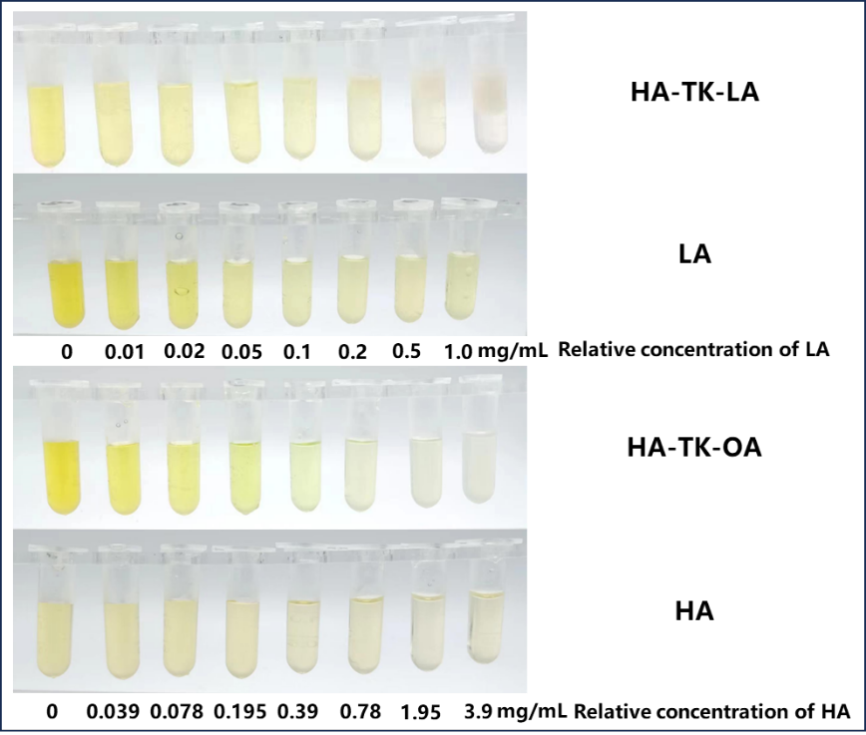
**

**Fig. S7** Schematic illustration of color changes in reaction solutions: Hydroxyl radicals (·OH) generated by the FeCl_3_/H_2_O_2_ system oxidize 3,3’,5,5’-tetramethylbenzidine (TMB) to produce a yellow product.


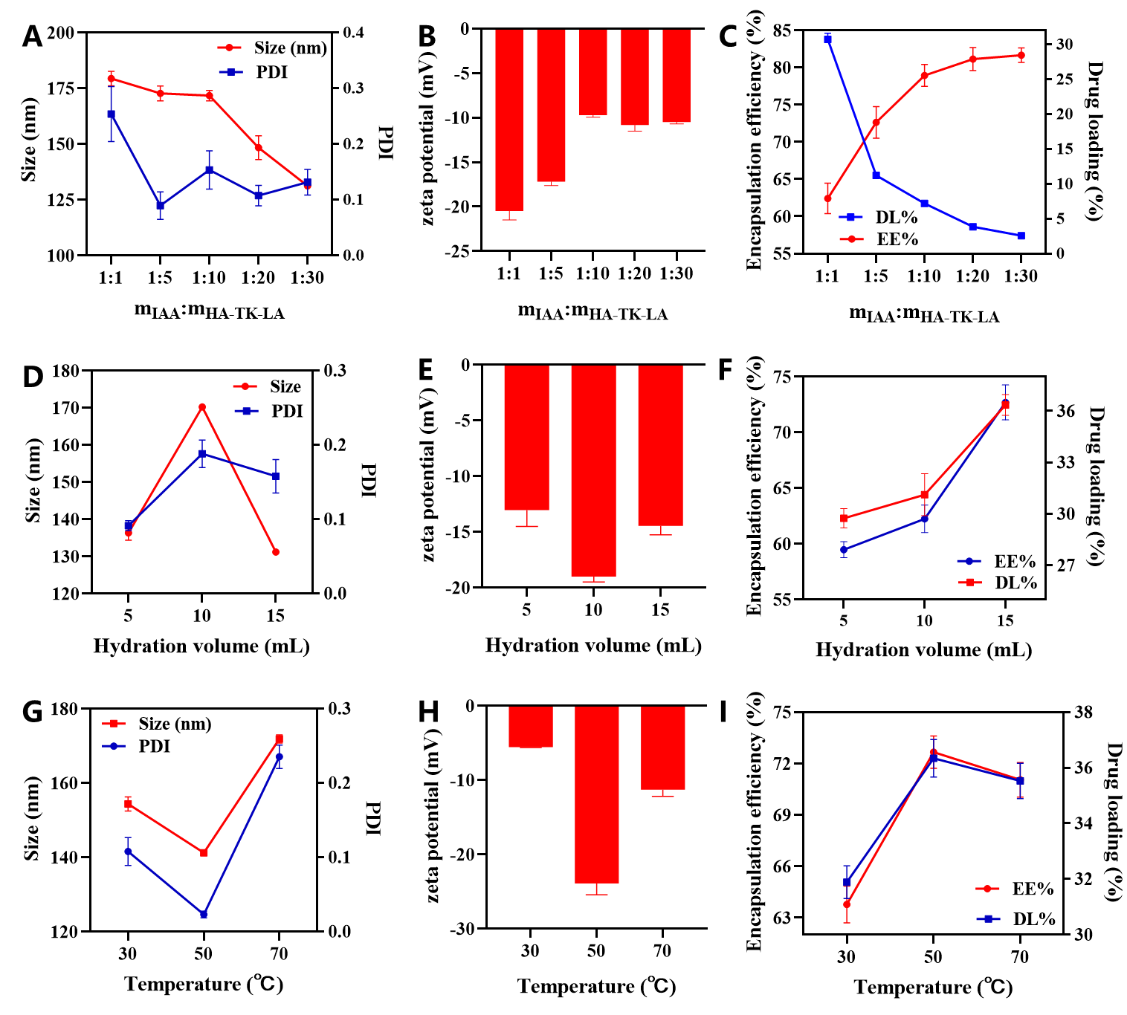


**Fig. S8** Optimization of the preparation process of IAA@HTL nanoparticles. **(A-C)** Effect of drug-to-material ratio (m_IAA_ : m_HTL_) on particle size/PDI **(A)**, Zeta potential **(B)**, drug loading (DL%), and encapsulation efficiency (EE%) **(C)**. **(D-F)** Effect of hydration volume on particle size/PDI **(D)**, Zeta potential **(E)**, DL%, and EE% **(F)**. **(G-I)** Effect of hydration temperature on particle size/PDI **(G)**, Zeta potential **(H)**, DL%, and EE% **(I)**. Data are presented as mean ± standard deviation (n=3).


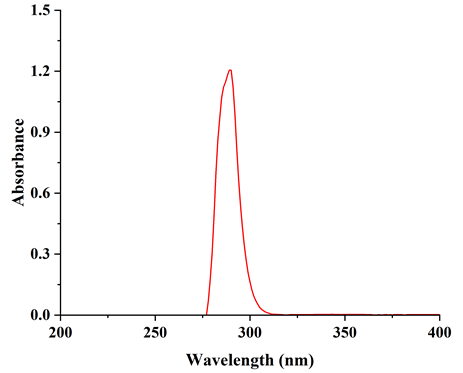


**Fig. S9** UV absorption spectrum of IAA standard solution (50 µg/mL) in the wavelength range of 200-400 nm.


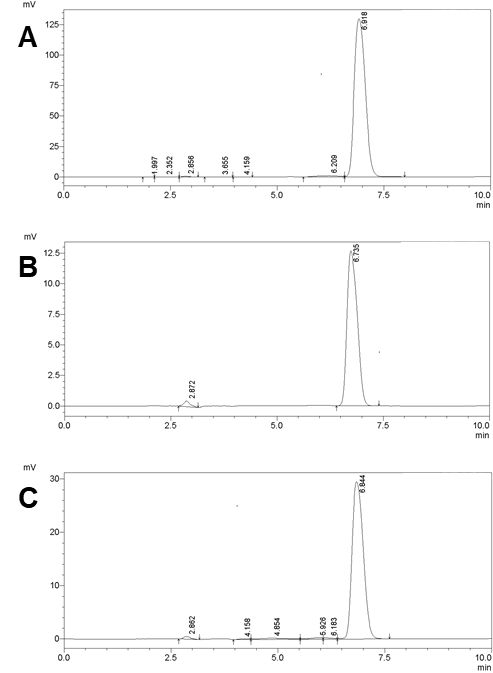


**Fig. S10** Specificity evaluation of the HPLC method for IAA detection: Chromatograms of IAA standard solution **(A)**, IAA@HTL nanoparticle lysate **(B)**, and their mixed solution **(C)**.


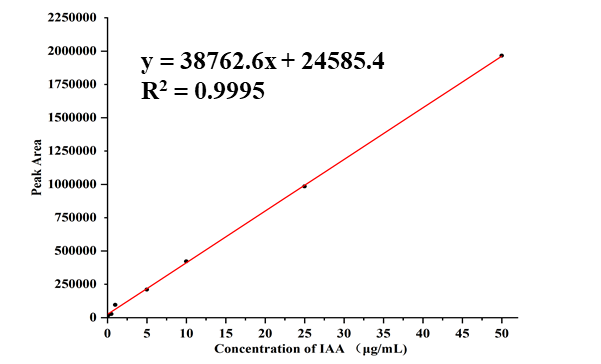


**Fig. S11** Standard curve of IAA (concentration range: 0.1-50 μg/mL).


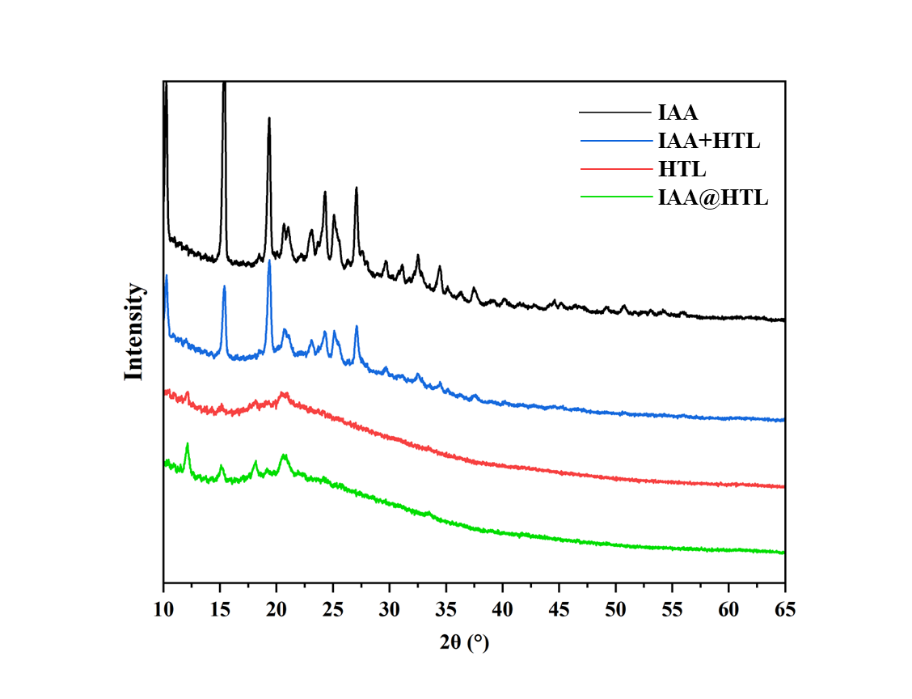


**Fig. S12** X-ray diffraction (XRD) patterns of IAA@HTL NPs and their control components.


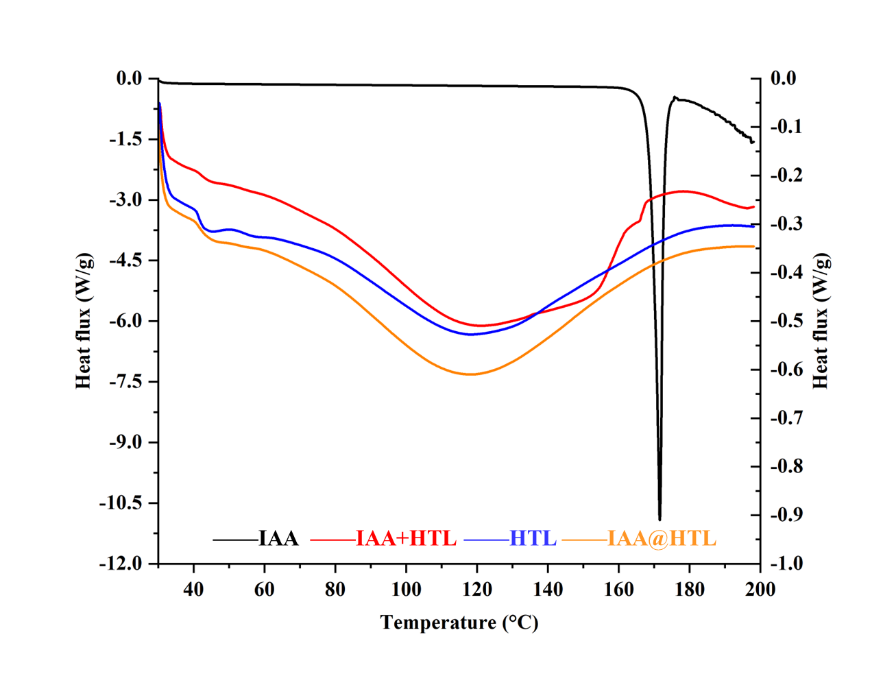


**Fig. S13** Differential scanning calorimetry (DSC) thermograms of IAA@HTL NPs and their control components.


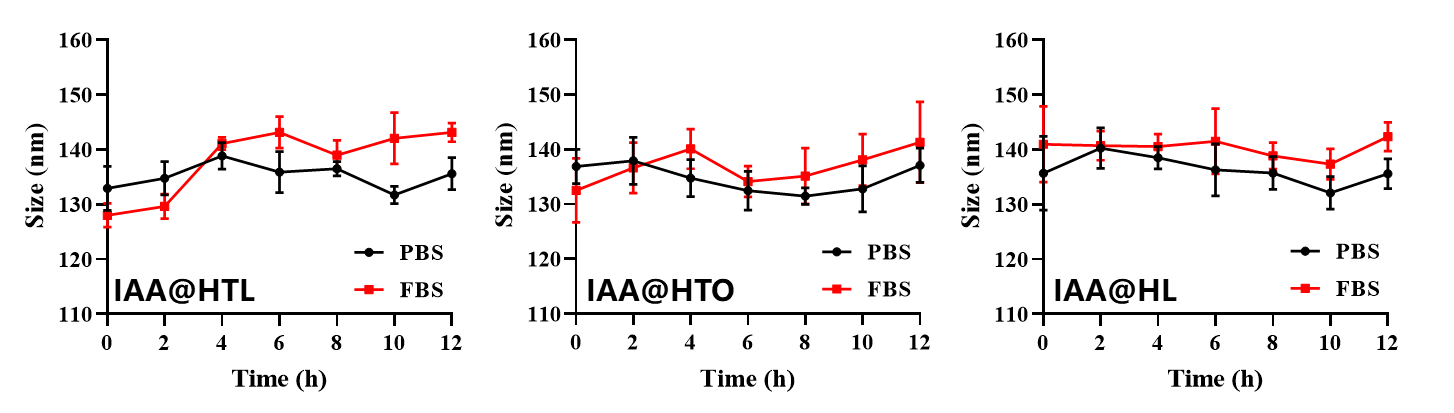


**Fig. S14** Evaluation of nanoparticle size stability in different physiological media: incubation in pH 7.4 PBS buffer and pH 7.4 PBS buffer containing 20% fetal bovine serum (FBS). Data are presented as mean ± standard deviation (n=3).


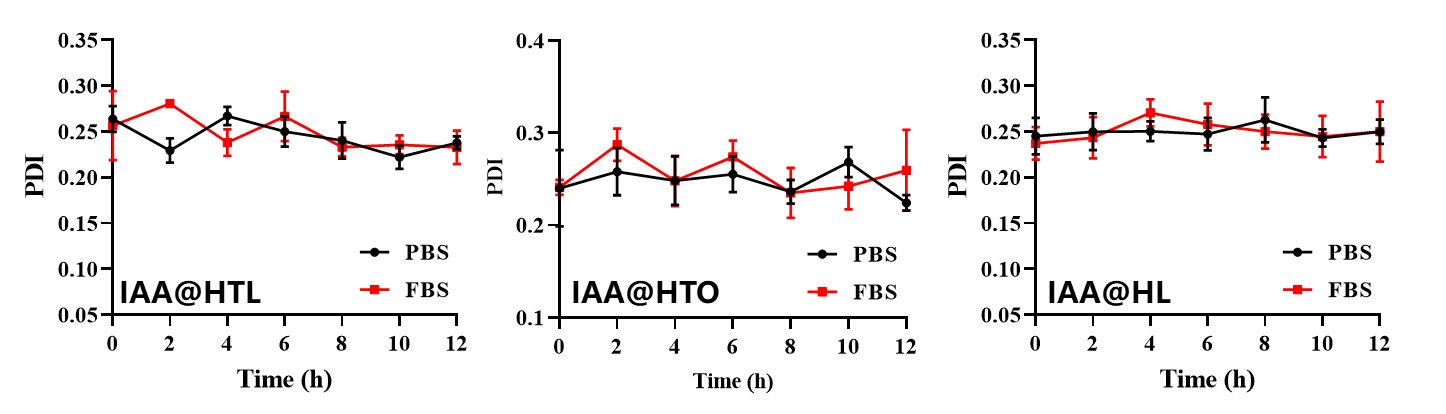


**Fig. S15** Changes in nanoparticle dispersibility in different physiological media: incubation solutions included pH 7.4 PBS buffer and pH 7.4 PBS buffer containing 20% fetal bovine serum (FBS). Data are expressed as mean ± standard deviation (n=3).


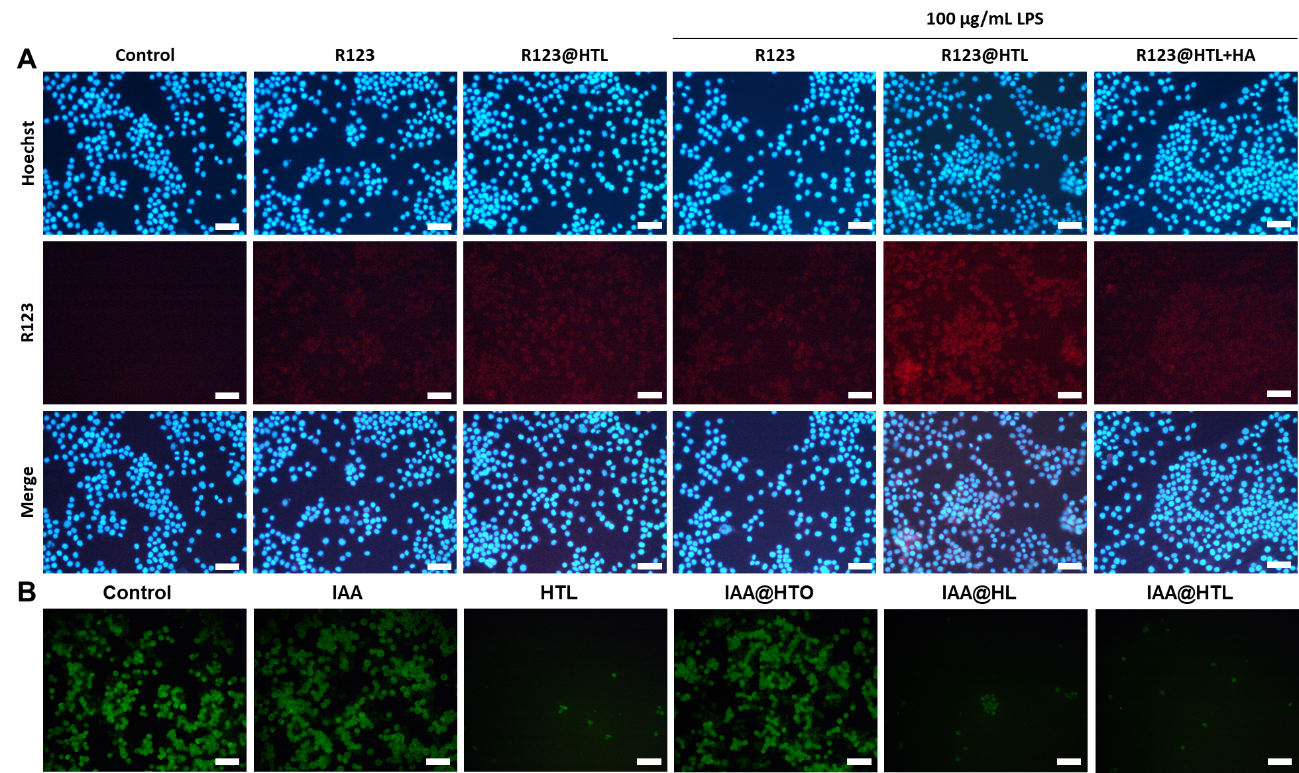


**Fig. S16 (A)** Cellular uptake behavior of rhodamine 123 (R123)-labeled nanoparticles: Fluorescence microscopy images of RAW264.7 cells after co-incubation with R123-loaded nanoparticles or free R123 solution (red: R123; blue: DAPI nuclear staining). Scale bar: 100 μm. **(B)** ROS scavenging capacity of different nanoparticles: Changes in DCFH-DA fluorescence intensity (green: ROS; blue: DAPI) in H_2_O_2_ (200 μmol/L)-induced RAW264.7 cells treated with different nanoparticles. Scale bar: 100 μm.


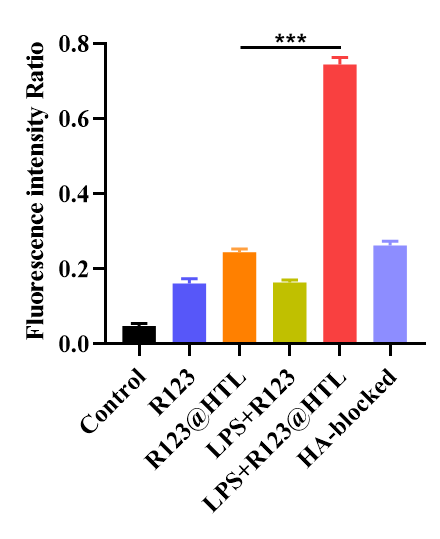


**Fig. S17** Semi-quantitative analysis of cellular uptake in RAW 264.7 cells. Data are expressed as mean ± SD (n=3), ^***^*p* < 0.001.


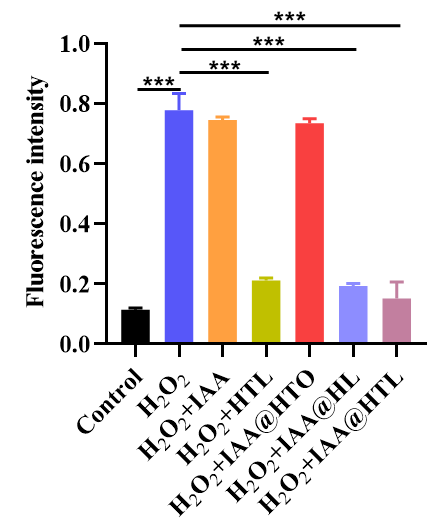


**Fig. S18** Semi-quantitative analysis of intracellular DCF fluorescence intensity in RAW 264.7 cells. Data represent mean ± SD (n=3). ^***^*p* < 0.001.


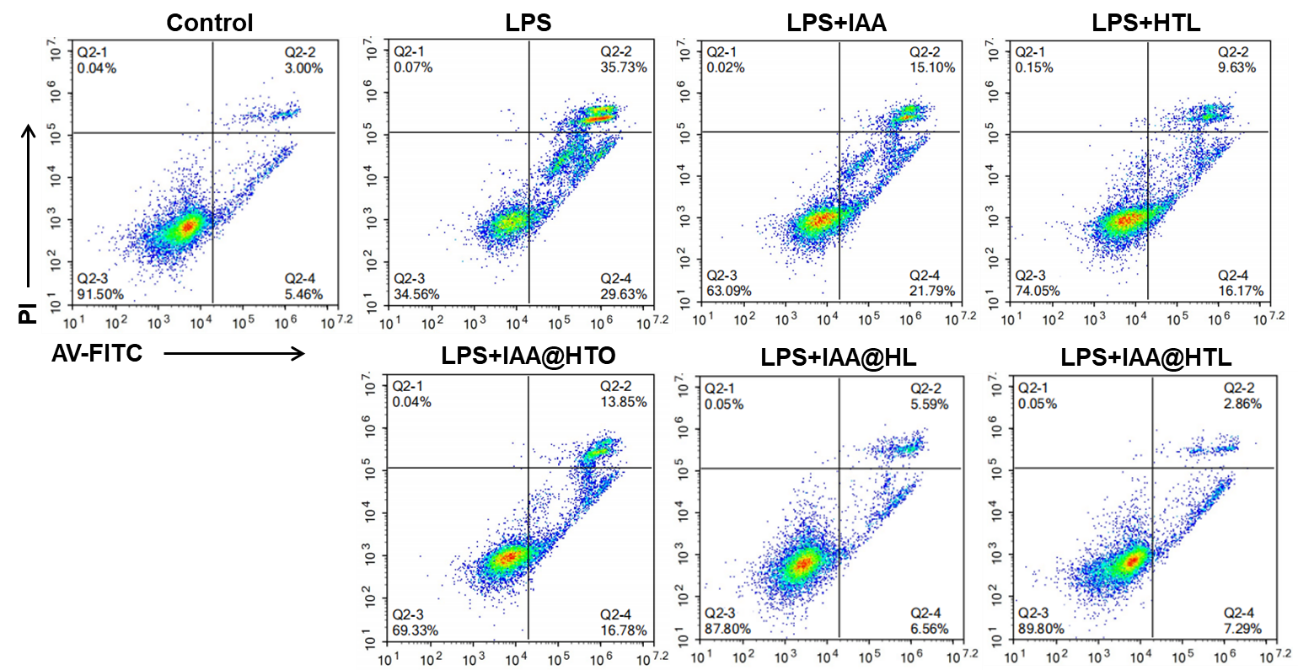


**Fig. S19** Flow cytometric analysis of the inhibitory effect of drug-loaded nanoparticles on LPS-induced apoptosis in HT-29 cells. Apoptosis rates were quantitatively analyzed using Annexin V-FITC/PI double staining. Quadrant definitions: upper left - necrotic cells/cellular debris; lower left - viable cells; upper right - late apoptotic cells; lower right - early apoptotic cells.


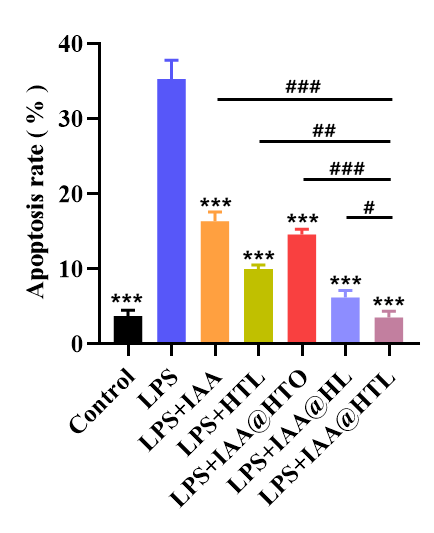


**Fig. S20** Anti-apoptotic effects of different nanoparticles: Flow cytometry quantitative analysis of apoptosis rate (Annexin V-FITC/PI double staining) in LPS-stimulated HT-29 cells. Data are presented as mean ± SD (n=3). ^#^*p* < 0.05, ^##^*p* < 0.01, ^###^*p* < 0.001, and ^*^*p* < 0.05, ^*^*p* < 0.01, ^***^*p* < 0.001 compared to the LPS group.


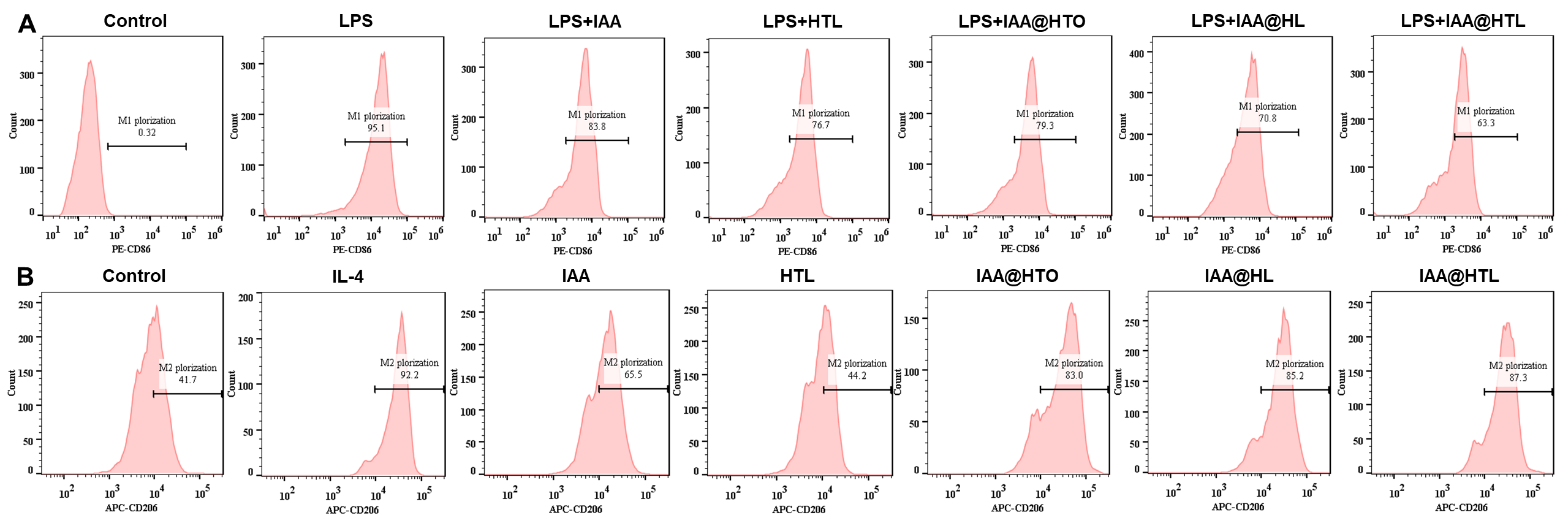


**Fig. S21** Flow cytometric analysis of M1-type (**A**, CD86) and M2-type (**B**, CD206) marker expression on RAW 264.7 cells treated with different nanoparticles.


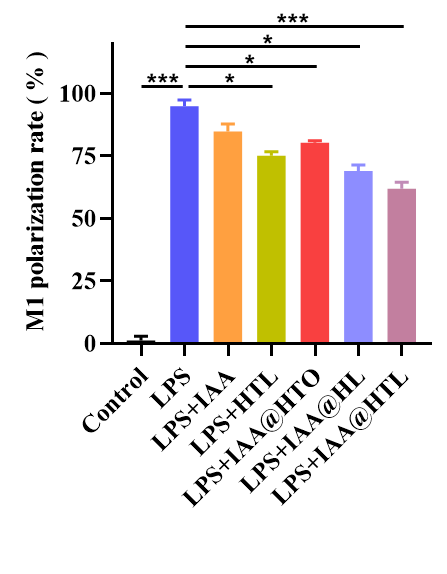


**Fig. S22** Quantitative flow cytometric analysis of drug-loaded nanoparticles’ effect on LPS-induced M1 polarization in RAW 264.7 cells. Data are presented as mean ± SD (n=3). ^*^*p* < 0.05, ^***^*p* < 0.001.


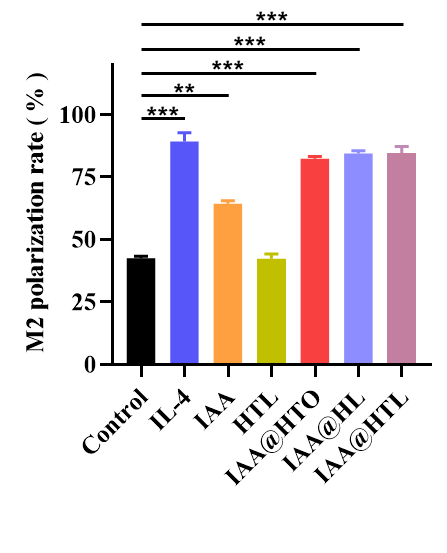


**Fig. S23** Flow cytometric quantification of drug-loaded nanoparticle-induced M2 polarization in RAW 264.7 cells. Data represent mean ± SD (n=3). ^**^*p* < 0.001, ^***^*p* < 0.001.


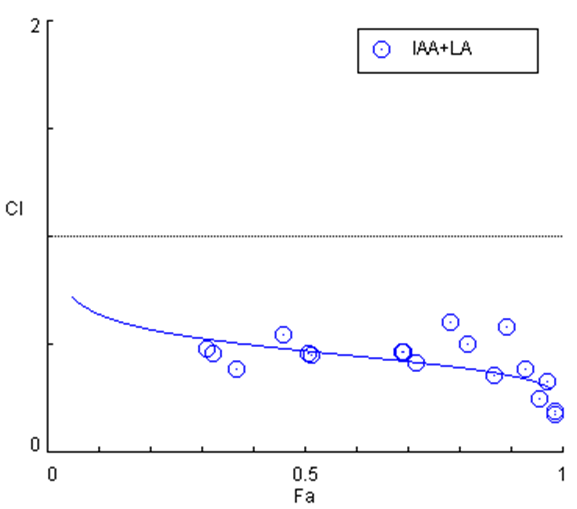


**Fig. S24** Combination index (CI)-fraction affected (Fa) plots for the combined administration of IAA and LA on inhibiting TNF-α secretion in LPS-stimulated Raw 264.7 cells

.


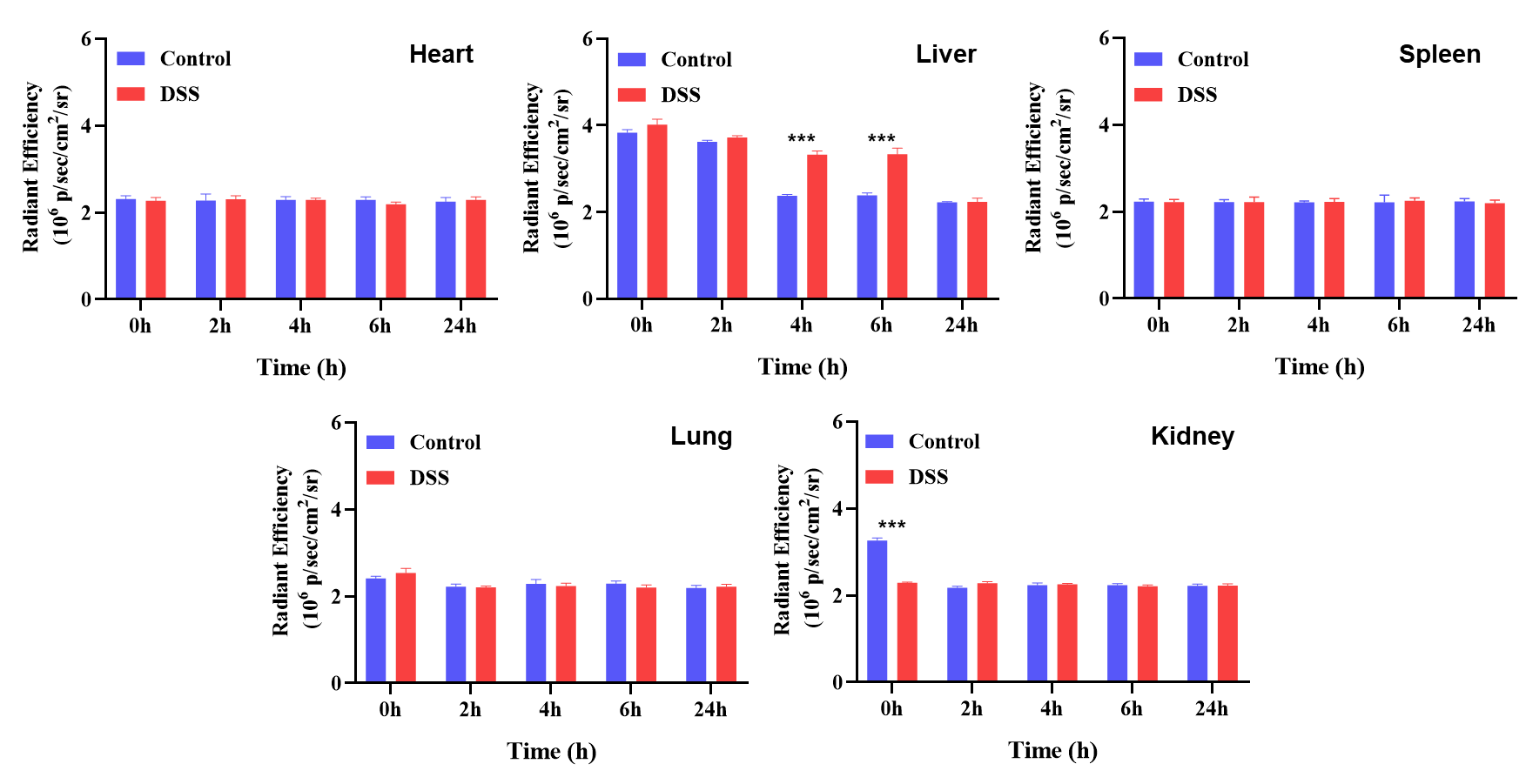


**Fig. S25** Targeted quantitative analysis: Temporal changes in R123 fluorescence intensity in heart, liver, spleen, lung, and kidney tissues. Data are expressed as mean ± SD (n=3). ^***^*p* < 0.001.


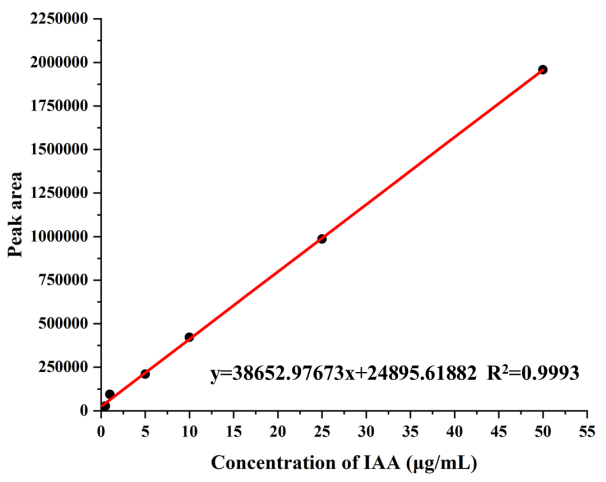


**Fig. S26** Linear relationship between peak area and concentration of IAA in plasma.


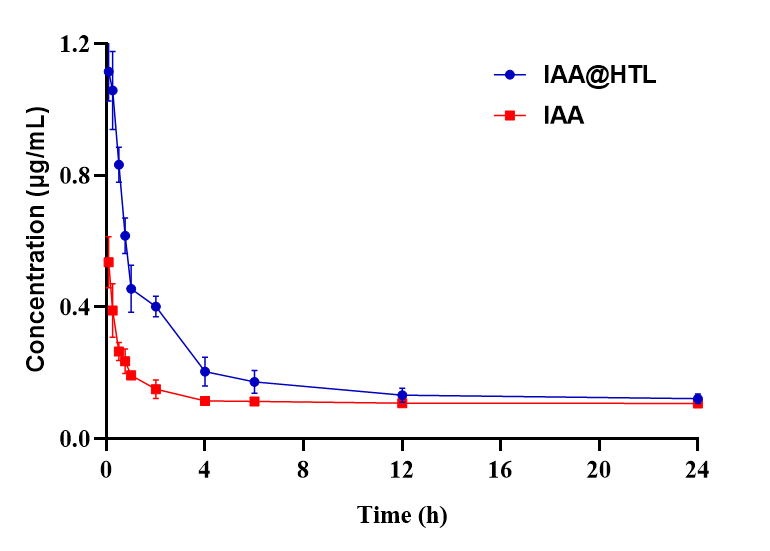


**Fig. S27** Plasma concentration curves of IAA (i.v. administration of 0.5 mg/kg) and IAA@HTL (i.v. administration of NPs containing 0.5 mg/kg IAA) in rats. Mean ± SD, n = 6.


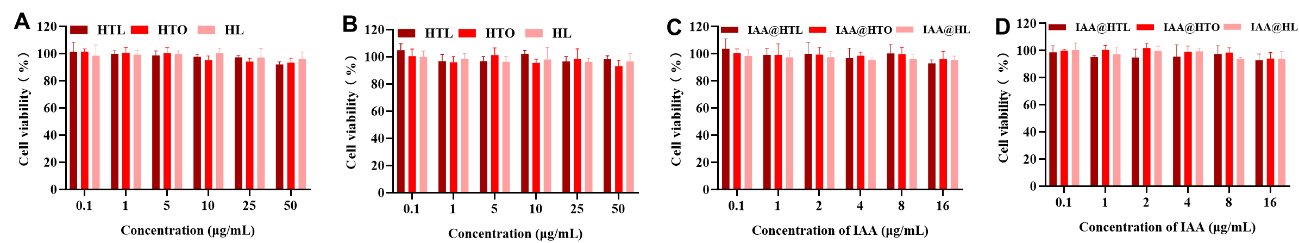


**Fig. S28 (A, B)** Carrier toxicity - Effects of HTL, HTO, and HL polymers at various concentrations (0.1-50 μg/mL, based on HA) on proliferation of HT-29 cells **(A)** and RAW 264.7 cells **(B)** (MTT assay); **(C, D)** Drug-loaded nanoparticles toxicity - Viability of HT-29 cells **(C)** and RAW 264.7 cells **(D)** treated with IAA@HTL, IAA@HTO, and IAA@HL (0.1-16 μg/mL, based on IAA). Data are presented as mean ± SD (n=3).


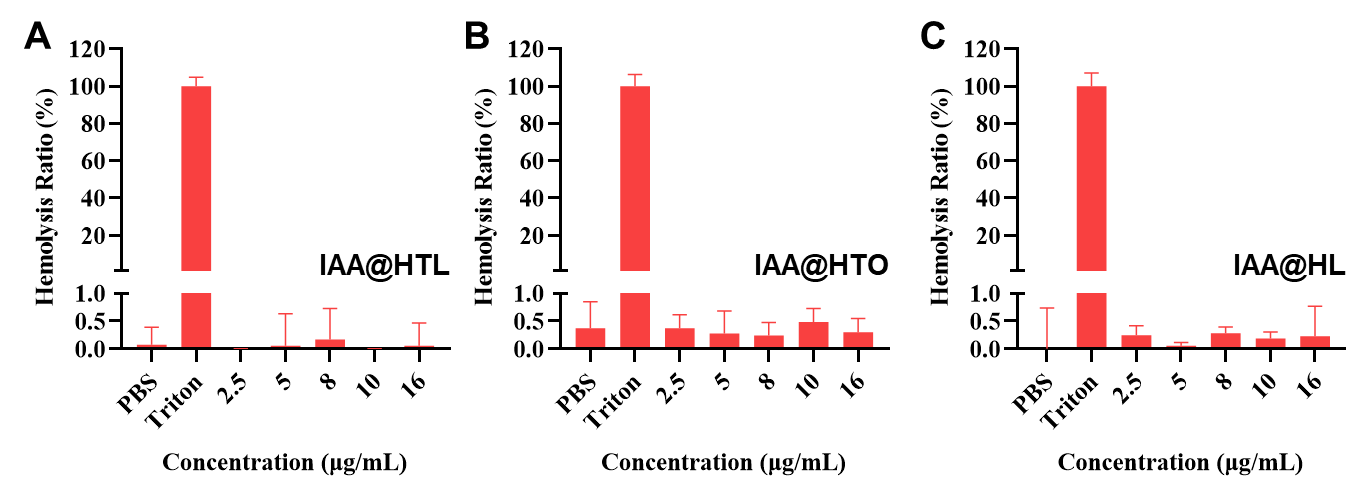


**Fig. S29** Hemocompatibility tests: Hemolysis rates of IAA@HTL **(A)**, IAA@HTO **(B)**, and IAA@HL **(C)** at different concentrations (0-500 μg/mL).


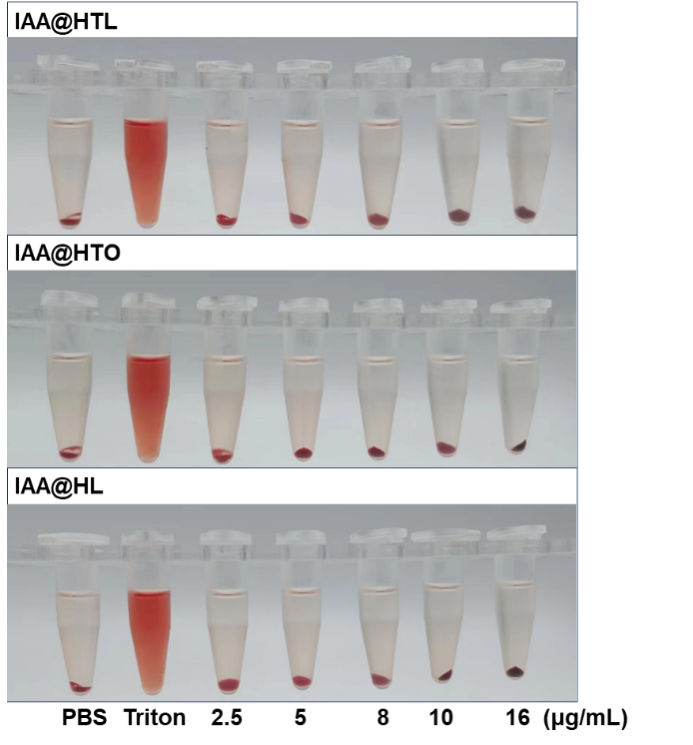


**Fig. S30** Photographic documentation of *in vitro* hemolysis assay for drug-loaded nanoparticles.


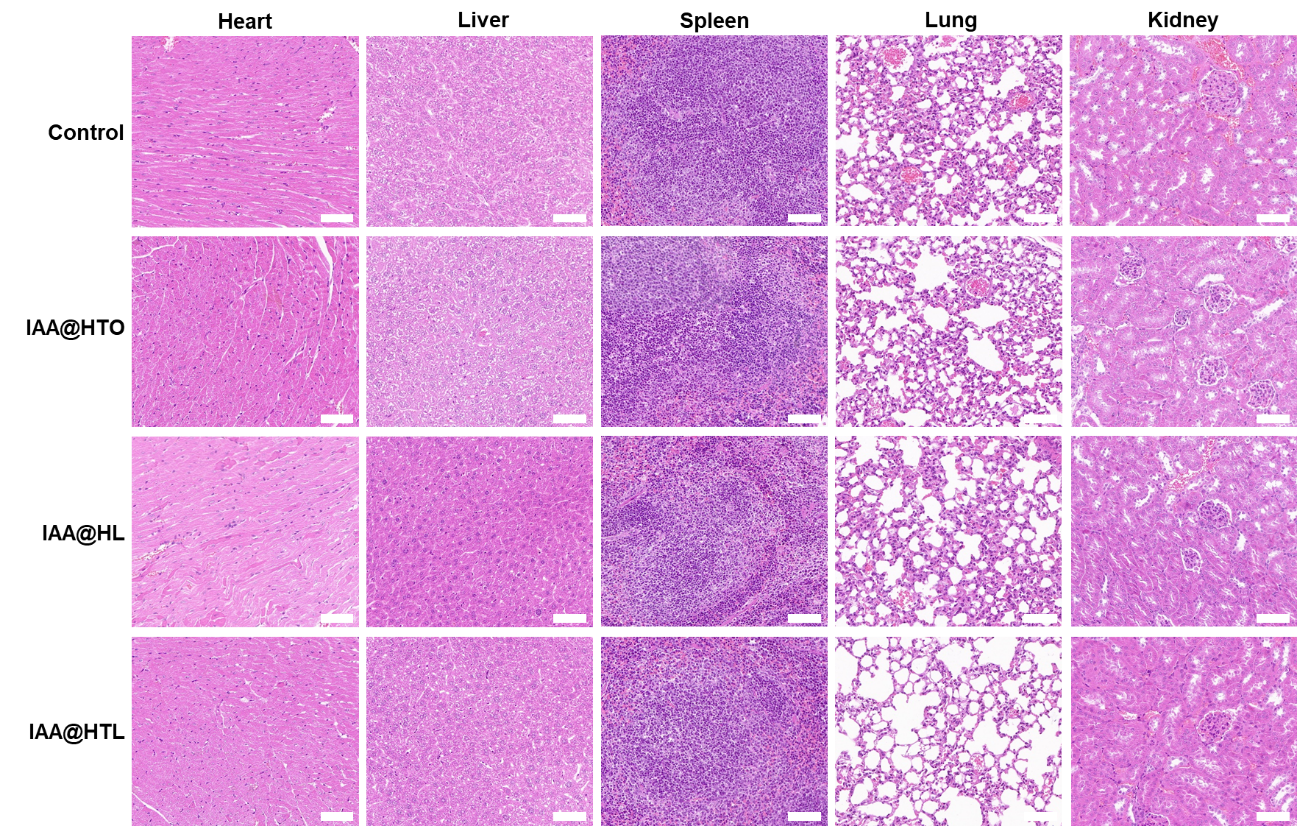


**Fig. S31** H&E-stained images of major organs from healthy mice after intravenous injection of drug-loaded nanoparticles. Scale bar: 50 μm.


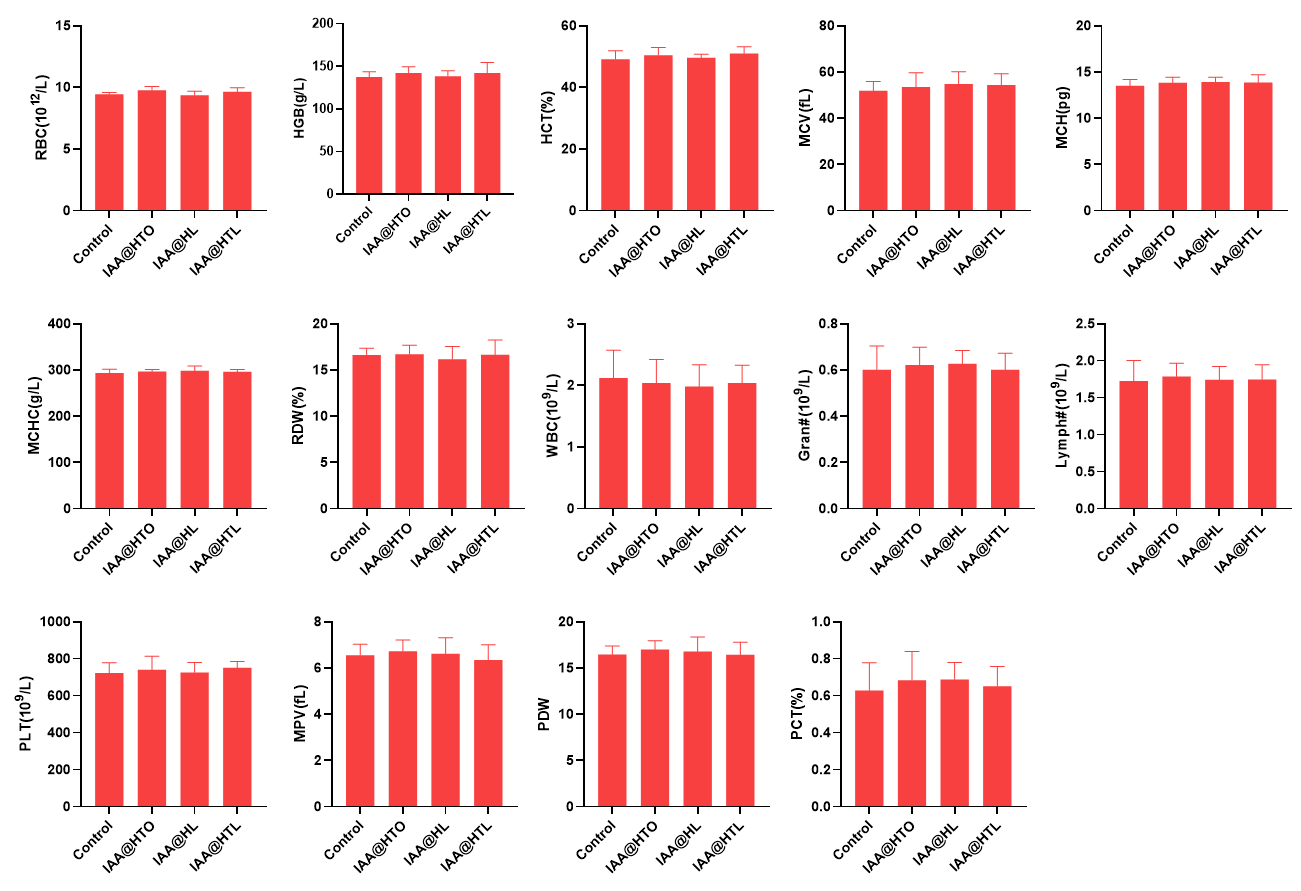


**Fig. S32** Major hematological parameters of healthy mice after intravenous injection of drug-loaded nanoparticles. Data are presented as mean ± SD (n=5).


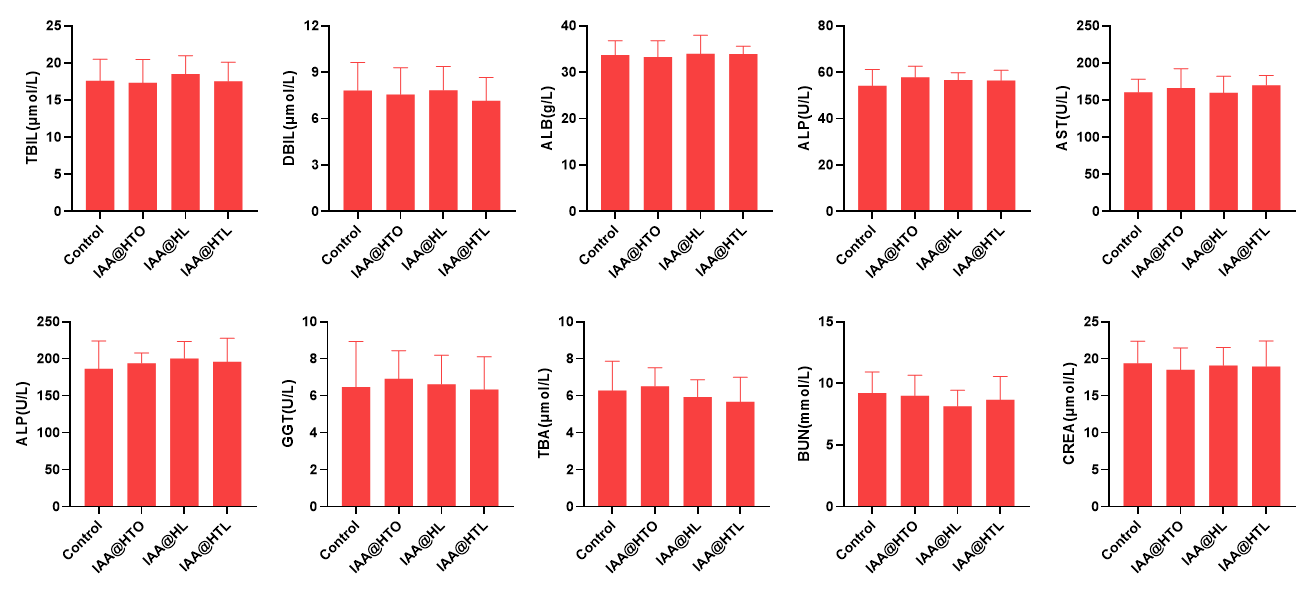


**Fig. S33** Major hepatic and renal function parameters in healthy mice following intravenous injection of drug-loaded nanoparticles. Data are expressed as mean ± SD (n=5).


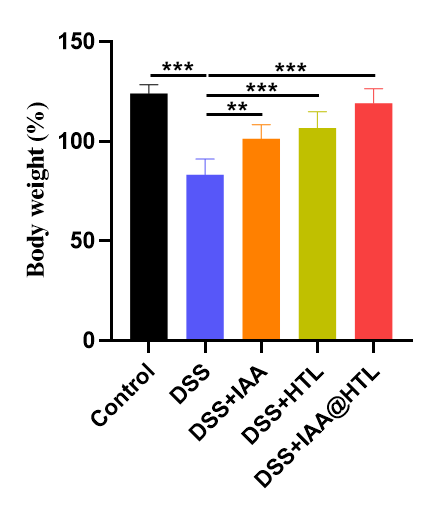


**Fig. S34** Body weights of mice in each group on the final day of treatment. Data are presented as mean ± SD (n=6). ^**^*p* < 0.001, ^***^*p* < 0.001.


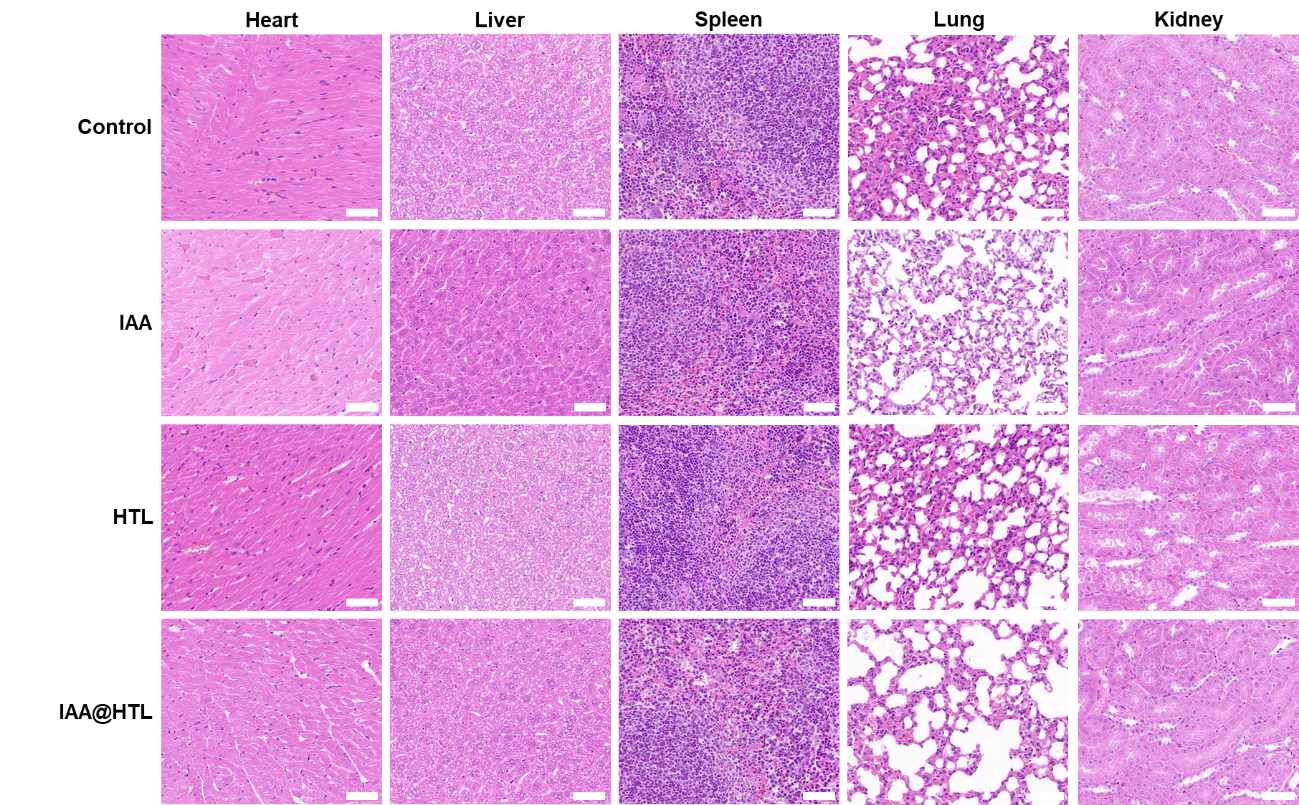


**Fig. S35** Representative H&E-stained images of major organs from different treatment groups. Scale bar: 50 μm.


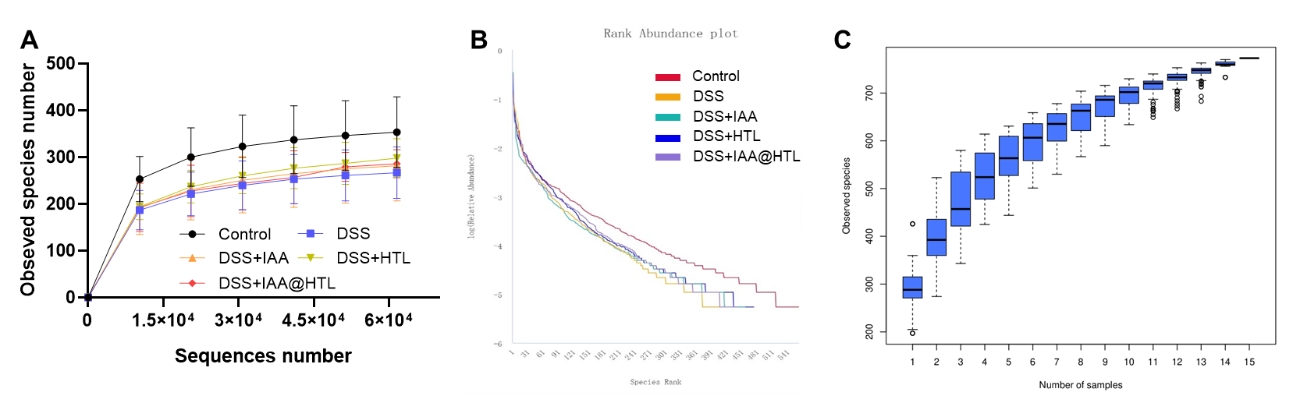


**Fig. S36** Analysis of microbial community diversity across treatment groups: **(A)** Rarefaction curves, **(B)** Rank-abundance curves, and **(C)** Species accumulation boxplots. Data are presented as mean ± SD (n=5).


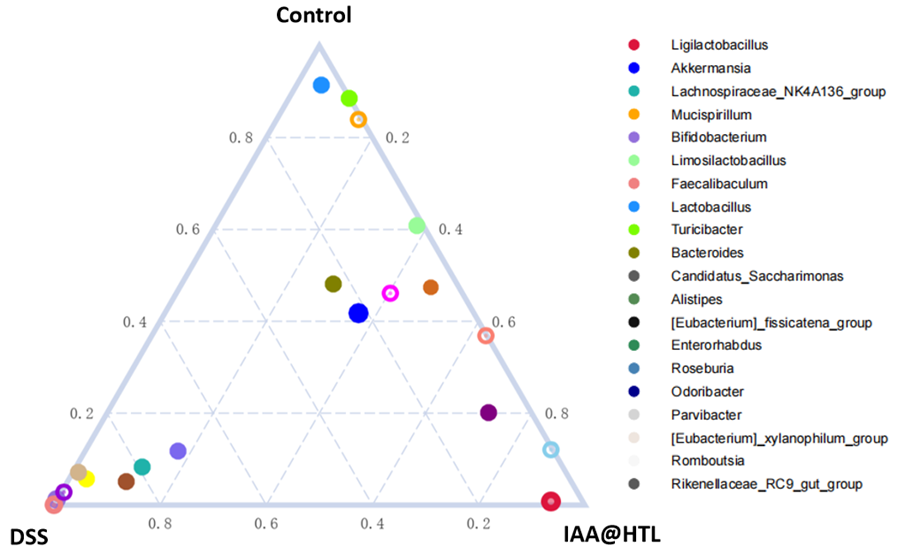


**Fig. S37** Ternary plot analysis of IAA@HTL-induced alterations in gut microbiota composition at the species level.

**Table S1** Chromatographic conditions for IAA concentration detection by HPLC

| Wavelength | Column Temperature | Flow Rate | Elution Mode | Mobile Phase | HPLC Column |
| --- | --- | --- | --- | --- | --- |
| 280 nm | 25 ℃ | 1.0 mL/min | Isocratic elution | 20:80 (*v:v*) methanol/water with 2% glacial acetic acid | BDS Hypersil C18 |

**Table S2** Precision for IAA determination by HPLC (n = 5)

| Concentration(µg/mL) | Intra-day precision RSD (%) | Inter-day precision RSD (%) |
| --- | --- | --- |
| 1 | 2.28 | 2.30 |
| 10 | 2.06 | 2.98 |
| 50 | 2.57 | 2.80 |

**Table S3** Stability for IAA determination by HPLC (n = 3)

| Sample | No. | Concentration (µg/mL) | | | | | | RSD |
| --- | --- | --- | --- | --- | --- | --- | --- | --- |
|  |  | 0 h | 2 h | 4 h | 8 h | 12 h | 24 h | (%) |
| IAA  50 µg/mL | 1 | 50.13 | 50.46 | 49.99 | 50.74 | 50.28 | 50.97 | 0.74 |
|  | 2 | 49.89 | 50.51 | 49.79 | 50.23 | 50.33 | 50.49 | 0.60 |
|  | 3 | 49.79 | 50.46 | 50.05 | 50.21 | 49.73 | 50.77 | 0.80 |

**Table S4** Spiked recovery for Dsp and Dex determination by HPLC (Mean ± SD, n = 3)

| Concentration | Adding sample | Mean ± SD | Recovery_s_ | RSD |
| --- | --- | --- | --- | --- |
| (µg/mL) | (%) | (µg/mL) | (%) | (%) |
| 1 | 80 | 1.81±0.02 | 101.04±2.97 | 2.94 |
| 1 | 100 | 2.00±0.03 | 100.27±2.88 | 2.87 |
| 1 | 120 | 2.21±0.01 | 100.78±1.39 | 1.38 |
| 5 | 80 | 9.16±0.08 | 103.10±1.59 | 1.55 |
| 5 | 100 | 10.16±0.13 | 103.13±0.03 | 2.52 |
| 5 | 120 | 10.99±0.04 | 99.76±0.82 | 0.82 |
| 10 | 80 | 17.98±0.02 | 99.79±0.28 | 0.28 |
| 10 | 100 | 20.19±0.19 | 101.84±1.93 | 1.90 |
| 10 | 120 | 22.01±0.07 | 100.04±1.93 | 0.62 |

**Table S5** Particle size, PDI, zeta potential, drug loading capacity, and encapsulation efficiency data of IAA@HTO and IAA@HL nanoparticles (Mean ± SD, n = 3)

|  | IAA@HTO | IAA@HL |
| --- | --- | --- |
| Particle size (nm) | 130.14±5.67 | 141.21±7.83 |
| PDI | 0.11±0.03 | 0.12±0.05 |
| Zeta potential (mV) | -25.36±3.21 | -27.33±5.43 |
| drug loading capacity (DL%) | 40.18%±2.46% | 34.46%±1.15% |
| encapsulation efficiency (EE%) | 80.36%±4.67% | 68.92±3.52% |

**Table S6** Precision for IAA in plasma detected by HPLC (n = 5)

| Concentration(µg/mL) | Intra-day precision RSD (%) | Inter-day precision RSD (%) |
| --- | --- | --- |
| 1 | 2.36 | 2.54 |
| 10 | 2.14 | 2.69 |
| 50 | 2.41 | 2.85 |

**Table S7** The stability of IAA mixed in plasma analyzed by HPLC (n = 5)

| Sample | No. | Concentration (µg/mL) | | | | | | RSD |
| --- | --- | --- | --- | --- | --- | --- | --- | --- |
|  |  | 0 h | 2 h | 4 h | 8 h | 12 h | 24 h | (%) |
| IAA  50 µg/mL | 1 | 50.22 | 50.36 | 49.95 | 50.52 | 50.31 | 50.66 | 0.49 |
|  | 2 | 50.12 | 49.87 | 49.98 | 50.22 | 50.37 | 50.47 | 0.46 |
|  | 3 | 49.82 | 50.38 | 50.22 | 50.05 | 49.84 | 50.27 | 0.46 |

**Table S8** Standard Addition Recovery of IAA in Plasma by HPLC (Mean ± SD，n = 3)

| Concentration | Adding sample | Mean ± SD | Recovery_s_ | RSD |
| --- | --- | --- | --- | --- |
| (µg/mL) | (%) | (µg/mL) | (%) | (%) |
| 1 | 80 | 1.81±0.03 | 100.83±0.04 | 1.78 |
| 1 | 100 | 2.03±0.06 | 102.67±0.06 | 2.89 |
| 1 | 120 | 2.23±0.04 | 102.22±0.01 | 1.82 |
| 5 | 80 | 8.99±0.05 | 99.83±0.01 | 0.55 |
| 5 | 100 | 9.99±0.14 | 99.93±0.03 | 1.34 |
| 5 | 120 | 10.95±0.10 | 99.16±0.02 | 0.91 |
| 10 | 80 | 18.10±0.13 | 101.29±0.02 | 0.70 |
| 10 | 100 | 20.12±0.15 | 101.17±0.02 | 0.76 |
| 10 | 120 | 22.09±0.31 | 100.72±0.03 | 1.40 |

**Table S9** Pharmacokinetic parameter of IAA (i.v. administration of 0.5 mg/kg) and IAA@HTL NPs (i.v. administration of NPs containing 0.5 mg/kg IAA) in rats (Mean ± SD, n = 6).

| Parameter | IAA | IAA@HTL |
| --- | --- | --- |
| C_max_ (μg/mL) | 0.54±0.08 | 1.12±0.09^**^ |
| t_1/2_ (min) | 260.35±63.96 | 549.12±86.06^**^ |
| AUC_0~t_(min·μg/mL) | 175.80±5.48 | 282.03±33.52^**^ |
| AUC_0~∞_(min·μg/mL) | 481.30±117.90 | 600.72±188.05^**^ |
| CL (mL/min) | 0.46±0.02 | 0.22±0.06^**^ |
| MRT_0~t_(min) | 510.11±26.45 | 643.27±17.39^**^ |
| MRT_0~∞_(min) | 1574.89±266.94 | 2048.30±368.04^**^ |

Compared with IAA, ^**^*P* < 0.01
